# Synthesis, Biological Evaluation and Molecular Docking Study of 2-Substituted-4,6-Diarylpyrimidines as α-Glucosidase Inhibitors

**DOI:** 10.3390/molecules22111865

**Published:** 2017-10-30

**Authors:** Zipeng Gong, Zhenzhen Xie, Jie Qiu, Guangcheng Wang

**Affiliations:** 1 Provincial Key Laboratory of Pharmaceutics in Guizhou Province, Guizhou Medical University, Beijing Road, Guiyang 550004, China; gzp4012607@126.com; 2 School of Pharmacy, Guizhou Medical University, 4 Beijing Road, Guiyang 550004, China; 3 National Engineering Research Center of Miao’s Medicines, 4 Beijing Road, Guiyang 550004, China; 4 College of Chemistry and Chemical Engineering, Jishou University, Jishou 416000, China; xiezz1993@126.com (Z.X.); qiujie_jsu@126.com (J.Q.)

**Keywords:** α-glucosidase, molecular docking, pyrimidine, chalcone

## Abstract

A novel series of 2-substituted-4,6-diarylpyrimidines **6a**–**6t** has been synthesized, characterized by ^1^H-NMR, ^13^C-NMR and HRMS, and screened for in vitro *α*-glucosidase inhibitory activity. The majority of the screened compounds possessed significant α-glucosidase inhibitory activity with IC_50_ values ranging from 19.6 ± 0.21 to 38.9 ± 0.35 μM, which is more potent than the positive control α-glucosidase inhibitor acarbose (IC_50_ = 817.38 ± 6.27 μM). Among them, **6j** was found to be the most active compound against α-glucosidase with an IC_50_ of 19.6 ± 0.21 μM. In addition, molecular docking studies were carried out to explore the binding interactions of 2-substituted-4,6-diarylpyrimidine derivatives with α-glucosidase.

## 1. Introduction

Diabetes mellitus is a group of metabolic diseases characterized by hyperglycemia (high blood sugar) that result from insulin deficiency (type I) and/or insensitivity of insulin receptors (type II) [1]. Long-term chronic hyperglycemia can cause serious complications, including damage to the nerves, the vessels, and several organs, including the eyes, heart and kidneys [2]. α-Glucosidase is a membrane-bound enzyme located at the epithelium of small intestine and catalyzes the final step in the digestion of carbohydrates. Thus, α-glucosidase inhibitors have been used as a first-line drug in type II diabetes treatment, because they can delay the carbohydrate digestion process in the small intestine and control postprandial blood glucose level [3]. Currently, several *α*-glucosidase inhibitors containing sugar moieties, including acarbose, voglibose, and miglitol, are used clinically in the treatment of diabetes [4]. 

**Figure 1:**
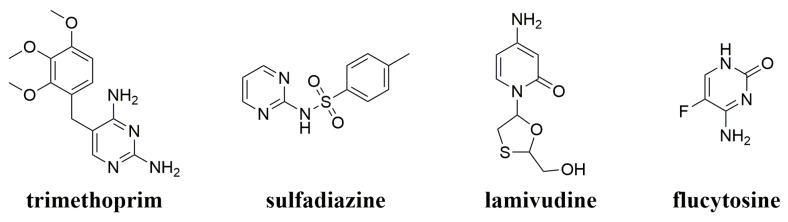
The structures of some commercial drugs containing pyrimidine pharmacophore.

Pyrimidines are very important pharmacophores in medicinal chemistry, and exhibit a broad spectrum of biological activities, including antibacterial [5], antifungal [6], anti-inflammatory [7], antihypertensive [8], antiviral [9], antidiabetic [10], anticonvulsant [11] and anticancer activities [12]. In addition to this, pyrimidine skeleton is also present in many natural products such as vitamin B1 (thiamine) and many clinically used drugs, such as trimethoprim, sulfadiazine, lamivudine, flucytosine (Figure 1) [13]. In particular, some pyrimidine and fused pyrimidine derivatives have been reported as potent α-glucosidase inhibitors [14,15,16,17,18]. Yar et al. reported the synthesis of dihydropyrimidines by an inexpensive and non-toxic *N*-acetyl glycine (NAG) catalysed reaction of aromatic aldehydes with ethylacetoacetate and urea/thiourea, and the majority of compounds synthesized displayed modest inhibitory activity against *α*-glucosidase at low micro-molar concentrations [14]. Suresh et al. developed a simple and facile method for the synthesis of fused tetrazolo[1,5-*a*]pyrimidine derivatives based on the multicomponent reaction of acetophenone, dimethylformamidedimethylacetal and 5-aminotetrazole, and some of compounds were found to be active, showing inhibitory potency comparable to acarbose [18].

Prompted by these observations, and in continuation of our interest in the synthesis of chemically and biologically important heterocycles [19,20,21,22], we herein report the synthesis of a novel series of 2-substituted-4,6-diarylpyrimidine derivatives. All the synthesized compounds were tested for their in vitro α-glucosidase inhibitory activity. Molecular docking was also performed on the most active compound to investigate the existence of interactions between the most active inhibitor and α-glucosidase.

## 2. Results and Discussion

### 2.1. Chemistry

**Scheme 1:**
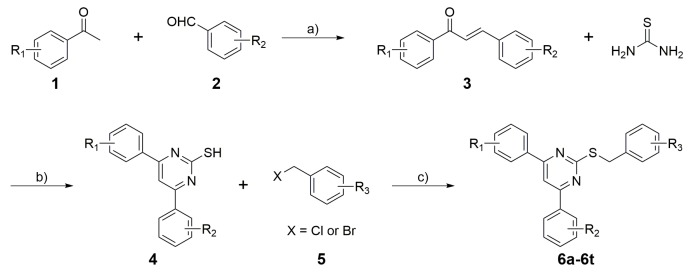
(a) KOH, MeOH, room temperature, 48 h, yield = 53–75%; (b) KOH, EtOH, reflux, 4 h, yield = 68–81%; (c) K_2_CO_3_, DMF, room temperature, 12 h, yield = 45–75% (**6a**–**6j**, **6l**–**6q**, **6s**, **6t**: X = Cl; **6k**, **6r**: X = Br).

A general synthesis of 2-substituted-4,6-diarylpyrimidines **6a**–**6t** is shown in Scheme 1. The reaction of commercially available acetophenones **1** with various aromatic aldehydes **2** in the presence of KOH in methanol provided corresponding substituted chalcone **3**. Treatment of substituted chalcone **3** with thiourea in the presence of KOH in reflux EtOH afforded intermediate **4**, which reacted with various benzyl chlorides or benzyl bromides **5** to provide the products **6a**–**6t**. None of the compounds have yet been reported in the literature. Structures of the compounds were elucidated by ^1^H-NMR, ^13^C-NMR and HRMS (Supplementary Materials). For instance, the ^1^H-NMR spectrum of **6a** showed a singlet at *δ* 4.68 ppm due to methylene protons of SCH_2_. Four 2-chlorobenzyl protons appeared as two multiplets for three protons between *δ* 7.27–7.30 ppm and *δ* 7.49–7.51 ppm, and a doublet of one proton at *δ* 7.63 ppm with a *J* value of 7.2 Hz. The protons of the 4-bromophenyl groups appeared as two doublets for four protons each at *δ* 7.76 and *δ* 8.27 ppm. The single peak of the CH proton of pyrimidine was observed at *δ* 8.37 ppm.

The ^13^C-NMR spectrum of **6a** showed a characteristic peak for methylene carbon at *δ* 32.9 ppm. The number of remaining signals equalled the number of different carbons in the compound **6a**. Therefore, the data for ^1^H-NMR and ^13^C-NMR are in agreement with the structure of compound **6a**.

### 2.2. α-Glucosidase Inhibition Assay

All of the newly synthesized 2-substituted-4,6-diarylpyrimidines were tested for their in vitro α-glucosidase inhibitory activity. Results are summarized in Table 1. The majority of the screened compounds possessed significant α-glucosidase inhibitory activity, with IC_50_ values ranging from 19.6 ± 0.21 to 38.9 ± 0.35 μM, more potent than the positive control α-glucosidase inhibitor acarbose (IC_50_ = 817.38 ± 6.27 μM, the value of IC_50_ was similar to that reported previously in the literature [23,24]), which is currently clinically used as an anti-diabetic agent to control blood glucose level of patients. Compounds **6a**, **6b**, **6d**, **6j**, **6k**, **6s** and **6t** displayed potent inhibitory activities, with IC_50_ values of 20.4 ± 0.23, 23.7 ± 0.27, 29.0 ± 0.28, 19.6 ± 0.21, 21.2 ± 0.25, 26.2 ± 0.27 and 25.3 ± 0.28 μM, respectively. Among them, **6j** was found to be the most active compound against α-glucosidase, with an IC_50_ of 19.6 ± 0.21 μM. The remaining compounds displayed moderate to low α-glucosidase inhibitory activity.

### 2.3. Molecular Docking

**Figure 2:**
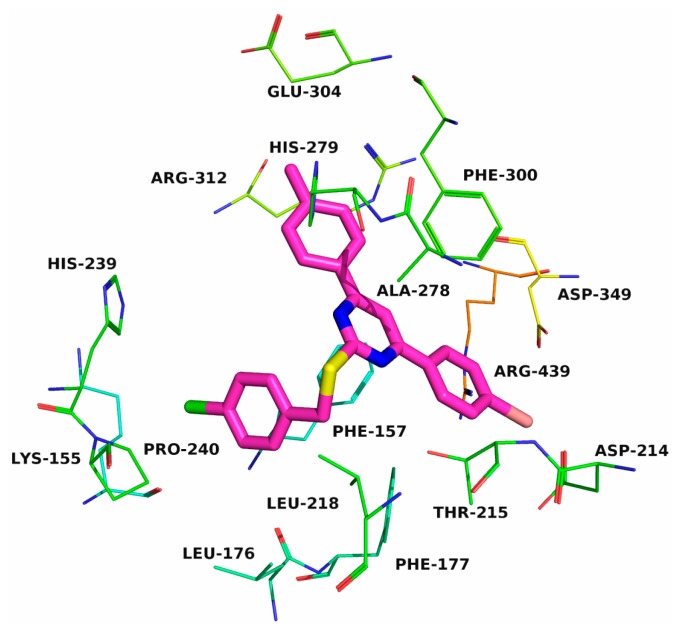
Compound **6j** was docked to the binding pocket of the *Saccharomyces cerevisiae* α-glucosidase.

A molecular docking study was performed to analyze the binding modes of this class of compound against α-glucosidase enzyme. The theoretical binding mode between **6j** and *Saccharomyces cerevisiae* α-glucosidase is shown in Figure 2. Compound **6j** adopted a “Y-shaped” conformation in the pocket of the α-glucosidase. The 4-chlorophenyl group of **6j** was located at the hydrophobic pocket, surrounded by the residues Phe-157, Leu-176, Phe-177, Leu-218 and Pro-240, forming a stable hydrophobic binding. Detailed analysis showed that the pyrimidine group in the middle of **6j** formed CH-π interactions with the residues Phe-157 and Phe-300. In addition, cation-π interactions were observed between **6j** and the residues Arg-312 and Arg-439. Also, **6j** formed anion-π interactions with the residues Asp-214, Glu-304 and Asp-349, respectively. All these interactions helped **6j** to anchor in the binding site of the α-glucosidase.

**Figure 3:**
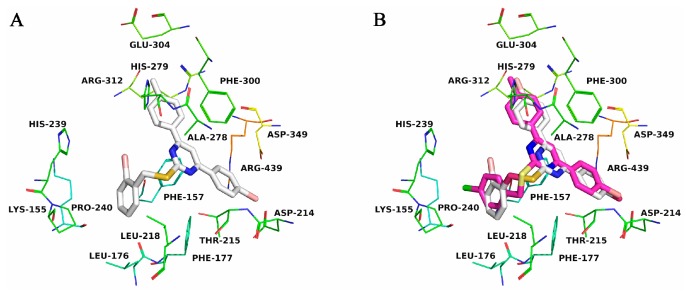
(**A**) Compound **6k** was docked to the binding pocket of the *Saccharomyces cerevisiae* α-glucosidase; (**B**) Compounds **6j** and **6k** were docked to the binding pocket of the *Saccharomyces cerevisiae* α-glucosidase (overlapped).

To explain the activity order of **6j** and **6k** against α-glucosidase, **6k** was then docked to the binding site of α-glucosidase; the theoretical binding mode between **6k** and α-glucosidase was shown in Figure 3A. The interaction between **6k** and α-glucosidase was almost the same as for the precursor **6j** (Figure 3B). The only difference was that **6j** formed the Cl-π interaction with the residue His-239, while **6k** didn’t, making **6j** a little more active than **6k** against α-glucosidase (Figure 3B). In addition, the estimated binding energies were −8.7 kcal·mol^−1^ for **6j** and −8.5 kcal·mol^−1^ for **6k**, which was consistent with the results of the in vitro anti-α-glucosidase assay. In summary, the above molecular simulations give us a rational explanation of the interactions between **6j**, **6k** and α-glucosidase, which provides valuable information for the further development of α-glucosidase inhibitors.

## 3. Materials and Methods

### 3.1. General

All starting materials and reagents were purchased from commercial suppliers. Nuclear magnetic resonance spectra (NMR) were recorded on a Bruker spectrometer (400 MHz, Bruker, Bremen, Germany) with TMS as an external reference and reported in parts per million.

### 3.2. General Procedure for the Synthesis of 2-Substituted-4,6-Diarylpyrimidines *(**6a**–**6t**)*

A mixture of **4** (1 mmol), different substituted benzyl chlorides or benzyl bromides **5** (1 mmol) and K_2_CO_3_ (1 mmol) in DMF (10 mL) was stirred at room temperature for 12 h. After the completion of the reaction, the mixture was poured into ice-cold water and the precipitated solid was collected by filtration, washed with water and dried in vacuo. The crude products were purified by column chromatography (silica gel) using EtOAc/petroleum ether as eluent.

*4,6-Bis(4-bromophenyl)-2-((2-chlorobenzyl)thio)pyrimidine* (**6a**). Eluent: EtOAc/petroleum ether (1:24). Colorless solid, 303 mg, yield 56%, m.p. 147–148 °C. ^1^H-NMR (*d*_6_-DMSO, 400 MHz) *δ*: 4.68 (s, 2H, SCH_2_), 7.27–7.30 (m, 2H, ArH), 7.49–7.51 (m, 1H, ArH), 7.63 (dd, 1H, *J* = 7.2 Hz, 2.0 Hz, ArH), 7.76 (d, 4H, *J* = 8.4 Hz, ArH), 8.27 (d, 4H, *J* = 8.4 Hz, ArH), 8.37 (s, 1H, CH-pyrimidine); ^13^C-NMR (*d*_6_-DMSO, 100 MHz) *δ*: 32.9, 108.9, 125.8, 127.9, 129.7, 129.9, 130.0, 131.4, 132.4, 133.8, 135.4, 135.6, 163.9, 171.1; HRMS (ESI) calcd for [M + H]^+^ C_23_H_16_Br_2_ClN_2_S^+^: 544.9084, found: 544.9071.

*4,6-Bis(4-bromophenyl)-2-((4-chlorobenzyl)thio)pyrimidine* (**6b**). Eluent: EtOAc/petroleum ether (1:24). Yellow solid, 362 mg, yield 66%, m.p. 176–178 °C. ^1^H-NMR (*d*_6_-DMSO, 400 MHz) *δ*: 4.55 (s, 2H, SCH_2_), 7.35 (d, 2H, *J* = 8.4 Hz, ArH), 7.50 (d, 2H, *J* = 8.4 Hz, ArH), 7.74 (d, 4H, *J* = 8.4 Hz, ArH), 8.25 (d, 4H, *J* = 8.4 Hz, ArH), 8.34 (s, 1H, CH-pyrimidine); ^13^C-NMR (*d*_6_-DMSO, 100 MHz) *δ*: 34.1, 108.8, 125.8, 128.8, 129.9, 131.0, 132.1, 132.4, 135.4, 137.7, 163.8, 171.2; HRMS (ESI) calcd for [M + H]^+^ C_23_H_16_Br_2_ClN_2_S^+^: 544.9084, found: 544.9064.

*4,6-Bis(4-bromophenyl)-2-((3-fluorobenzyl)thio)pyrimidine* (**6c**). Eluent: EtOAc/petroleum ether (1:24). Yellow solid, 325 mg, yield 61%, m.p. 111–113 °C. ^1^H-NMR (*d*_6_-DMSO, 400 MHz) *δ*: 4.57 (s, 2H, SCH_2_), 7.04–7.08 (m, 1H, ArH), 7.30–7.36 (m, 3H, ArH), 7.74 (d, 4H, *J* = 8.8 Hz, ArH), 8.25 (d, 4H, *J* = 8.8 Hz, ArH), 8.35 (s, 1H, CH-pyrimidine); ^13^C-NMR (*d*_6_-DMSO, 100 MHz) *δ*: 34.3, 108.8, 114.2 (d, 1C, *J* = 20.7 Hz), 115.8 (d, 1C, *J* = 21.6 Hz), 125.3 (d, 1C, *J* = 2.7 Hz), 125.8, 129.9, 130.8 (d, 1C, *J* = 8.4 Hz), 132.4, 135.4, 141.6 (d, 1C, *J* = 7.5 Hz), 161.3 (d, 1C, *J* = 242.1 Hz), 163.8, 171.2; HRMS (ESI) calcd for [M + H]^+^ C_23_H_16_Br_2_FN_2_S^+^: 528.9379, found: 528.9382.

*4,6-Bis(4-bromophenyl)-2-((2-fluorobenzyl)thio)pyrimidine* (**6d**). Eluent: EtOAc/petroleum ether (1:24). Yellow solid, 407 mg, yield 77%, m.p. 149–150 °C. ^1^H-NMR (*d*_6_-DMSO, 400 MHz) *δ*: 4.59 (s, 2H, SCH_2_), 7.11–7.15 (m, 1H, ArH), 7.20–7.24 (m, 1H, ArH), 7.28–7.33 (m, 1H, ArH), 7.55–7.59 (m, 1H, ArH), 7.75 (d, 4H, *J* = 8.8 Hz, ArH), 8.26 (d, 4H, *J* = 8.8 Hz, ArH), 8.35 (s, 1H, CH-pyrimidine); ^13^C-NMR (*d*_6_-DMSO, 100 MHz) *δ*: 28.4, 108.9, 115.7 (d, 1C, *J* = 21.2 Hz), 125.0 (d, 1C, *J* = 3.5 Hz), 125.2 (d, 1C, *J* = 14.6 Hz), 125.8, 129.8 (d, 1C, *J* = 7.2 Hz), 129.9, 131.4 (d, 1C, *J* = 3.8 Hz), 132.4, 135.4, 159.7 (d, 1C, *J* = 244.0 Hz), 163.9, 171.1; HRMS (ESI) calcd for [M + H]^+^ C_23_H_16_Br_2_FN_2_S^+^: 528.9379, found: 528.9377..

*4,6-Bis(4-bromophenyl)-2-((4-fluorobenzyl)thio)pyrimidine* (**6e**). Eluent: EtOAc/petroleum ether (1:24). Yellow solid, 240 mg, yield 45 %, m.p. 144–146 °C. ^1^H-NMR (*d*_6_-DMSO, 400 MHz) *δ*: 4.55 (s, 2H, SCH_2_), 7.14 (t, 2H, *J* = 8.8 Hz, ArH), 7.50 (dd, 2H, *J* = 8.8 Hz, 2.0 Hz, ArH), 7.75 (d, 4H, *J* = 8.4 Hz, ArH), 8.26 (d, 4H, *J* = 8.8 Hz, ArH), 8.35 (s, 1H, CH-pyrimidine); ^13^C-NMR (*d*_6_-DMSO, 100 MHz) *δ*: 34.1, 108.8, 115.6 (d, 2C, *J* = 21.3 Hz), 125.8, 129.9, 131.1 (d, 2C, *J* = 7.2 Hz), 132.4, 134.7 (d, 1C, *J* = 3.0 Hz), 135.4, 160.5 (d, 1C, *J* = 241.6 Hz), 163.8, 171.4; HRMS (ESI) calcd for [M + H]^+^ C_23_H_16_Br_2_FN_2_S^+^: 528.9379, found: 528.9390.

*4-(3-Bromophenyl)-2-((2-chlorobenzyl)thio)-6-(p-tolyl)pyrimidine* (**6f**). Eluent: EtOAc/petroleum ether (1:24). Yellow solid, 334 mg, yield 69%, m.p. 107–110 °C. ^1^H-NMR (*d*_6_-DMSO, 400 MHz) *δ*: 2.39 (s, 3H, CH_3_), 4.67 (s, 2H, SCH_2_), 7.26–7.30 (m, 2H, ArH), 7.35 (d, 2H, *J* = 8.8 Hz, ArH), 7.49–7.53 (m, 2H, ArH), 7.63–7.66 (m, 1H, ArH), 7.74 (dd, 1H, *J* = 8.0 Hz, 1.2 Hz, ArH), 8.24 (d, 2H, *J* = 8.0 Hz, ArH), 8.31–8.33 (m, 2H, ArH), 8.48 (s, 1H, CH-pyrimidine); ^13^C-NMR (*d*_6_-DMSO, 100 MHz) *δ*: 21.5, 32.9, 122.9, 126.8, 127.8, 127.9, 129.6, 129.9, 130.0, 130.3, 131.2, 131.5, 133.4, 133.8, 134.4, 135.8, 138.7, 142.1, 163.1, 165.0, 170.8; HRMS (ESI) calcd for [M + H]^+^ C_24_H_19_BrClN_2_S^+^: 481.0135, found: 481.0135.

*4-(3-Bromophenyl)-2-((3-fluorobenzyl)thio)-6-(p-tolyl)pyrimidine* (**6g**). Eluent: EtOAc/petroleum ether (1:24). Yellow solid, 278 mg, yield 60%, m.p. 86–88 °C. ^1^H-NMR (*d*_6_-DMSO, 400 MHz) *δ*: 2.39 (s, 3H, CH_3_), 4.57 (s, 2H, SCH_2_), 7.03–7.12 (m, 1H, ArH), 7.32–7.37 (m, 5H, ArH), 7.50 (t, 1H, *J* = 8.0 Hz, ArH), 7.74 (dd, 1H, *J* = 8.0 Hz, 1.2 Hz, ArH), 8.23 (d, 2H, *J* = 8.4 Hz, ArH), 8.30–8.32 (m, 2H, ArH), 8.48 (s, 1H, CH-pyrimidine); ^13^C-NMR (*d*_6_-DMSO, 100 MHz) *δ*: 21.5, 34.3, 108.8, 114.2 (d, 1C, *J* = 20.8 Hz), 115.8 (d, 1C, *J* = 21.6 Hz), 122.9, 125.2 (d, 1C, *J* = 2.7 Hz), 126.8, 127.9, 130.0, 130.3, 130.7 (d, 1C, *J* = 8.4 Hz), 131.4, 133.4, 134.4, 138.7, 141.7 (d, 1C, *J* = 7.4 Hz), 142.1, 161.3 (d, 1C, *J* = 242.3 Hz), 163.0, 165.0, 171.0; HRMS (ESI) calcd for [M + H]^+^ C_24_H_19_BrFN_2_S^+^: 465.0431, found: 465.0430.

*4-(3-Bromophenyl)-2-((4-chlorobenzyl)thio)-6-(p-tolyl)pyrimidine* (**6h**). Eluent: EtOAc/petroleum ether (1:24). Yellow solid, 298 mg, yield 62%, m.p. 127–129 °C. ^1^H-NMR (*d*_6_-DMSO, 400 MHz) *δ*: 2.39 (s, 3H, CH_3_), 4.55 (s, 2H, SCH_2_), 7.35–7.37 (m, 4H, ArH), 7.51–7.53 (m, 3H, ArH), 7.74 (d, 1H, *J* = 8.0 Hz, ArH), 8.23 (d, 2H, *J* = 8.0 Hz, ArH), 8.30–8.32 (m, 2H, ArH), 8.47 (s, 1H, CH-pyrimidine); ^13^C-NMR (*d*_6_-DMSO, 100 MHz) *δ*: 21.5, 34.1, 108.8, 122.9, 126.8, 127.9, 128.8, 130.0, 130.3, 131.0, 131.5, 132.1, 133.4, 134.4, 137.8, 138.8, 142.1, 163.0, 165.0, 171.0; HRMS (ESI) calcd for [M + H]^+^ C_24_H_19_BrClN_2_S^+^: 481.0135, found: 481.0137.

*4-(3-Bromophenyl)-2-((2-fluorobenzyl)thio)-6-(p-tolyl)pyrimidine* (**6i**). Eluent: EtOAc/petroleum ether (1:24). Yellow solid, 222 mg, yield 48%, m.p. 100–104 °C. ^1^H-NMR (*d*_6_-DMSO, 400 MHz) *δ*: 2.39 (s, 3H, CH_3_), 4.59 (s, 2H, SCH_2_), 7.13 (t, 1H, *J* = 8.0 Hz, ArH), 7.21 (t, 1H, *J* = 8.8 Hz, ArH), 7.28–7.31 (m, 1H, ArH), 7.35 (d, 2H, *J* = 8.0 Hz, ArH), 7.51 (t, 1H, *J* = 8.0 Hz, ArH), 7.58 (t, 1H, *J* = 8.0 Hz, ArH), 7.75 (d, 1H, *J* = 8.0 Hz, ArH), 8.24 (d, 2H, *J* = 8.0 Hz, ArH), 8.32–8.33 (m, 2H, ArH), 8.49 (s, 1H, CH-pyrimidine); ^13^C-NMR (*d*_6_-DMSO, 100 MHz) *δ*: 21.5, 28.3, 108.8, 115.7 (d, 1C, *J* = 21.2 Hz), 122.9, 124.9 (d, 1C, *J* = 3.4 Hz), 125.3 (d, 1C, *J* = 14.6 Hz), 126.8, 127.9, 129.7 (d, 1C, *J* = 8.1 Hz), 130.0, 130.3, 131.3 (d, 1C, *J* = 3.9 Hz), 131.4, 133.4, 134.4, 138.7, 142.1, 159.7 (d, 1C, *J* = 243.8 Hz), 163.1, 165.0, 170.9; HRMS (ESI) calcd for [M + H]^+^ C_24_H_19_BrFN_2_S^+^: 465.0431, found: 465.0421.

*4-(4-Bromophenyl)-2-((4-chlorobenzyl)thio)-6-(p-tolyl)pyrimidine* (**6j**). Eluent: EtOAc/petroleum ether (1:24). Colorless solid, 355 mg, yield 74%, m.p. 135–137 °C. ^1^H-NMR (*d*_6_-DMSO, 400 MHz) *δ*: 2.39 (s, 3H, CH_3_), 4.55 (s, 2H, SCH_2_), 7.35 (d, 4H, *J* = 8.0 Hz, ArH), 7.50 (d, 2H, *J* = 8.0 Hz, ArH), 7.74 (d, 2H, *J* = 8.0 Hz, ArH), 8.21 (d, 2H, *J* = 8.0 Hz, ArH), 8.25 (d, 2H, *J* = 8.0 Hz, ArH), 8.28 (s, 1H, CH-pyrimidine); ^13^C-NMR (*d*_6_-DMSO, 100 MHz) *δ*: 21.5, 34.1, 108.4, 125.6, 127.8, 128.8, 129.8, 130.0, 131.0, 132.1, 132.4, 133.4, 135.6, 137.9, 142.0, 163.5, 164.9, 171.0; HRMS (ESI) calcd for [M + H]^+^ C_24_H_19_BrClN_2_S^+^: 481.0135, found: 481.0146.

*2-((2-Bromobenzyl)thio)-4-(4-bromophenyl)-6-(p-tolyl)pyrimidine* (**6k**). Eluent: EtOAc/petroleum ether (1:24). Yellow solid, 324 mg, yield 62%, m.p. 123–126 °C. ^1^H-NMR (*d*_6_-DMSO, 400 MHz) *δ*: 2.39 (s, 3H, CH_3_), 4.67 (s, 2H, SCH_2_), 7.21–7.22 (m, 1H, ArH), 7.29–7.37 (m, 4H, ArH), 7.64–7.67 (m, 2H, ArH), 7.75 (d, 2H, *J* = 8.0 Hz, ArH), 8.22–8.29 (m, 5H, ArH + CH-pyrimidine); ^13^C-NMR (*d*_6_-DMSO, 100 MHz) *δ*: 21.5, 35.5, 108.5, 124.6, 125.6, 127.8, 128.4, 129.8, 130.0, 131.4, 132.4, 133.2, 133.4, 135.6, 137.4, 142.1, 163.5, 164.9, 170.8; HRMS (ESI) calcd for [M + H]^+^ C_24_H_19_Br_2_N_2_S^+^: 524.9630, found: 524.9639.

*4-(4-Bromophenyl)-2-((4-fluorobenzyl)thio)-6-(p-tolyl)pyrimidine* (**6l**). Eluent: EtOAc/petroleum ether (1:24). Yellow solid, 330 mg, yield 71%, m.p. 115–124 °C. ^1^H-NMR (*d*_6_-DMSO, 400 MHz) *δ*: 2.38 (s, 3H, CH_3_), 4.56 (s, 2H, SCH_2_), 7.13 (t, 2H, *J* = 8.0 Hz, ArH), 7.35 (d, 2H, *J* = 8.0 Hz, ArH), 7.52 (s, 2H, ArH), 7.74 (d, 2H, *J* = 8.0 Hz, ArH), 8.22–8.28 (m, 5H, ArH + CH-pyrimidine); ^13^C-NMR (*d*_6_-DMSO, 100 MHz) *δ*: 21.5, 34.0, 108.4, 115.5 (d, 2C, *J* = 21.3 Hz), 125.6, 127.8, 129.8, 130.0, 131.1 (d, 2C, *J* = 8.2 Hz), 132.4, 133.5, 134.9 (d, 1C, *J* = 3.1 Hz), 135.6, 142.0, 160.5 (d, 1C, *J* = 241.5 Hz), 163.5, 164.9, 171.1; HRMS (ESI) calcd for [M + H]^+^ C_24_H_19_BrFN_2_S^+^: 465.0431, found: 465.0434.

*4-(4-Bromophenyl)-2-((2-fluorobenzyl)thio)-6-(p-tolyl)pyrimidine* (**6m**). Eluent: EtOAc/petroleum ether (1:24). Yellow solid, 289 mg, yield 62%, m.p. 124–127 °C. ^1^H-NMR (*d*_6_-DMSO, 400 MHz) *δ*: 2.39 (s, 3H, CH_3_), 4.59 (s, 2H, SCH_2_), 7.12 (t, 1H, *J* = 8.0 Hz, ArH), 7.21 (t, 1H, *J* = 8.0 Hz, ArH), 7.28–7.33 (m, 1H, ArH), 7.35 (d, 2H, *J* = 8.0 Hz, ArH), 7.57 (t, 1H, *J* = 8.0 Hz, ArH), 7.74 (d, 2H, *J* = 8.8 Hz, ArH), 8.21–8.28 (m, 5H, ArH + CH-pyrimidine); ^13^C-NMR (*d*_6_-DMSO, 100 MHz) *δ*: 21.5, 28.3, 108.4, 115.7 (d, 1C, *J* = 21.2 Hz), 124.9 (d, 1C, *J* = 3.4 Hz), 125.3 (d, 1C, *J* = 14.6 Hz), 125.6, 127.8, 129.8, 129.8, 130.0, 131.4 (d, 1C, *J* = 3.9 Hz), 132.3, 133.4, 135.6, 142.1, 159.7 (d, 1C, *J* = 243.9 Hz), 163.5, 164.9, 170.8; HRMS (ESI) calcd for [M + H]^+^ C_24_H_19_BrFN_2_S^+^: 465.0431, found: 465.0439.

*4-(4-Bromophenyl)-2-((3-fluorobenzyl)thio)-6-(p-tolyl)pyrimidine* (**6n**). Eluent: EtOAc/petroleum ether (1:24). Yellow solid, 342 mg, yield 73%, m.p. 106–109 °C. ^1^H-NMR (*d*_6_-DMSO, 400 MHz) *δ*: 2.38 (s, 3H, CH_3_), 4.58 (s, 2H, SCH_2_), 6.99–7.16 (m, 1H, ArH), 7.25–7.44 (m, 5H, ArH), 7.69–7.82 (m, 2H, ArH), 8.23–8.28 (m, 5H, ArH + CH-pyrimidine); ^13^C-NMR (*d*_6_-DMSO, 100 MHz) *δ*: 21.5, 34.3, 108.4, 114.2 (d, 1C, *J* = 20.7 Hz), 115.8 (d, 1C, *J* = 21.6 Hz), 125.3 (d, 1C, *J* = 2.5 Hz), 125.6, 127.8, 129.8, 130.0, 130.7 (d, 1C, *J* = 8.4 Hz), 132.3, 133.4, 135.6, 141.7 (d, 1C, *J* = 7.6 Hz), 142.1, 161.3 (d, 1C, *J* = 242.3 Hz), 163.5, 164.9, 171.0; HRMS (ESI) calcd for [M + H]^+^ C_24_H_19_BrFN_2_S^+^: 465.0431, found: 465.0436.

*2-(Benzylthio)-4-(4-bromophenyl)-6-(p-tolyl)pyrimidine* (**6o**). Eluent: EtOAc/petroleum ether (1:24). Yellow solid, 216 mg, yield 48%, m.p. 129–130 °C. ^1^H-NMR (*d*_6_-DMSO, 400 MHz) *δ*: 2.38 (s, 3H, CH_3_), 4.56 (s, 2H, SCH_2_), 7.21–7.37 (m, 6H, ArH), 7.48 (d, 2H, *J* = 7.2 Hz, ArH), 7.74 (d, 2H, *J* = 8.4 Hz, ArH), 8.22–8.28 (m, 5H, ArH + CH-pyrimidine); ^13^C-NMR (*d*_6_-DMSO, 100 MHz) *δ*: 21.5, 34.9, 108.3, 125.6, 127.5, 127.8, 128.9, 129.2, 129.8, 130.0, 132.3, 133.5, 135.6, 142.0, 163.4, 164.8, 171.3; HRMS (ESI) calcd for [M + H]^+^ C_24_H_20_BrN_2_S^+^: 447.0525, found: 447.0519.

*4-(4-Bromophenyl)-2-((2,4-dichlorobenzyl)thio)-6-(p-tolyl)pyrimidine* (**6p**). Eluent: EtOAc/petroleum ether (1:24). Yellow solid, 345 mg, yield 67%, m.p. 156–157 °C. ^1^H-NMR (*d*_6_-DMSO, 400 MHz) *δ*: 2.39 (s, 3H, CH_3_), 4.65 (s, 2H, SCH_2_), 7.35–7.37 (m, 3H, ArH), 7.64 (m, 2H, ArH), 7.75 (d, 2H, *J* = 8.0 Hz, ArH), 8.21–8.30 (m, 5H, ArH + CH-pyrimidine); ^13^C-NMR (*d*_6_-DMSO, 100 MHz) *δ*: 21.5, 32.3, 108.6, 125.7, 127.8, 128.0, 129.4, 129.8, 130.0, 132.4, 132.5, 133.2, 134.7, 135.1, 135.5, 142.2, 163.6, 164.9, 170.6; HRMS (ESI) calcd for [M + H]^+^ C_24_H_18_BrCl_2_N_2_S^+^: 514.9746, found: 514.9756.

*4-(3-Bromophenyl)-2-((4-fluorobenzyl)thio)-6-(p-tolyl)pyrimidine* (**6q**). Eluent: EtOAc/petroleum ether (1:24). Yellow solid, 208 mg, yield 45%, m.p. 115–117 °C. ^1^H-NMR (*d*_6_-DMSO, 400 MHz) *δ*: 2.39 (s, 3H, CH_3_), 4.55 (s, 2H, SCH_2_), 7.13 (t, 2H, *J* = 8.8 Hz, ArH), 7.35 (d, 2H, *J* = 8.0 Hz, ArH), 7.49–7.55 (m, 3H, ArH), 7.75 (d, 1H, *J* = 7.2 Hz, ArH), 8.24 (d, 2H, *J* = 8.0 Hz, ArH), 8.32 (s, 2H, ArH), 8.49 (s, 1H, CH-pyrimidine); ^13^C-NMR (*d*_6_-DMSO, 100 MHz) *δ*: 21.5, 34.1, 108.7, 115.6 (d, 2C, *J* = 21.2 Hz), 122.9, 126.8, 127.9, 130.0, 130.3, 131.0 (d, 2C, *J* = 8.1 Hz), 131.5, 133.4, 134.4, 134.8 (d, 1C, *J* = 3.0 Hz), 138.8, 142.1, 160.5 (d, 1C, *J* = 241.5 Hz), 163.0, 165.0, 171.2; HRMS (ESI) calcd for [M + H]^+^ C_24_H_19_BrFN_2_S^+^: 465.0431, found: 465.0443.

*2-((4-Bromobenzyl)thio)-4-(3-bromophenyl)-6-(p-tolyl)pyrimidine* (**6r**). Eluent: EtOAc/petroleum ether (1:24). Yellow solid, 280 mg, yield 53%, m.p. 135–138 °C. ^1^H-NMR (*d*_6_-DMSO, 400 MHz) *δ*: 2.39 (s, 3H, CH_3_), 4.53 (s, 2H, SCH_2_), 7.35 (d, 2H, *J* = 8.0 Hz, ArH), 7.45–7.53 (m, 5H, ArH), 7.74 (d, 1H, *J* = 7.6 Hz, ArH), 8.23 (d, 2H, *J* = 8.0 Hz, ArH), 8.29–8.31 (m, 2H, ArH), 8.47 (s, 1H, CH-pyrimidine); ^13^C-NMR (*d*_6_-DMSO, 100 MHz) *δ*: 21.5, 34.2, 108.8, 120.5, 122.9, 126.8, 127.9, 130.0, 130.3, 130.6, 131.3, 131.5, 131.7, 131.8, 133.4, 134.4, 138.3, 138.7, 142.1, 163.0, 165.0, 171.0; HRMS (ESI) calcd for [M + H]^+^ C_24_H_19_Br_2_N_2_S^+^: 524.9630, found: 524.9644.

*4-(3-Bromophenyl)-6-(4-bromophenyl)-2-((2-chlorobenzyl)thio)pyrimidine* (**6s**). Eluent: EtOAc/petroleum ether (1:24). Colorless solid, 381 mg, yield 70%, m.p. 129–132 °C. ^1^H-NMR (*d*_6_-DMSO, 400 MHz) *δ*: 4.66 (s, 2H, SCH_2_), 7.28–7.37 (m, 2H, ArH), 7.49–7.53 (m, 2H, ArH), 7.63–7.65 (m, 1H, ArH), 7.75 (d, 3H, *J* = 8.0 Hz, ArH), 8.28–8.38 (m, 4H, ArH), 8.48 (s, 1H, CH-pyrimidine); ^13^C-NMR (*d*_6_-DMSO, 100 MHz) *δ*: 32.9, 109.2, 122.9, 125.9, 126.9, 127.8, 129.6, 129.9, 130.0, 130.4, 131.3, 131.5, 132.4, 133.8, 134.6, 135.3, 135.6, 138.5, 163.4, 164.0, 171.1; HRMS (ESI) calcd for [M + H]^+^ C_23_H_16_Br_2_ClN_2_S^+^: 544.9084, found: 544.9114.

*4-(3-Bromophenyl)-6-(4-bromophenyl)-2-((3-fluorobenzyl)thio)pyrimidine* (**6t**). Eluent: EtOAc/petroleum ether (1:24). Yellow solid, 397 mg, yield 75%, m.p. 94–96 °C. ^1^H-NMR (*d*_6_-DMSO, 400 MHz) *δ*: 4.57 (s, 2H, SCH_2_), 7.05–7.08 (m, 1H, ArH), 7.31–7.36 (m, 3H, ArH), 7.51 (t, 1H, *J* = 8.0 Hz, ArH), 7.74 (d, 3H, *J* = 8.4 Hz, ArH), 8.27–8.37 (m, 4H, ArH), 8.48 (s, 1H, CH-pyrimidine); ^13^C-NMR (*d*_6_-DMSO, 100 MHz) *δ*: 34.4, 109.1, 114.2 (d, 1C, *J* = 20.7 Hz), 115.8 (d, 1C, *J* = 21.6 Hz), 122.9, 125.3 (d, 1C, *J* = 3.6 Hz), 125.9, 126.9, 129.9, 130.4, 130.8 (d, 1C, *J* = 8.4 Hz), 131.5, 132.4, 134.6, 135.3, 138.5, 141.5 (d, 1C, *J* = 7.4 Hz), 161.3 (d, 1C, *J* = 242.1 Hz), 163.4, 163.9, 171.2; HRMS (ESI) calcd for [M + H]^+^ C_23_H_16_Br_2_FN_2_S^+^: 528.9379, found: 528.9393.

### 3.3. In Vitro Assay of α-Glucosidase Inhibitory Activity

α-Glucosidase inhibitory activity was assayed by using 0.1 M phosphate buffer (pH 6.8) at 37 °C. The enzyme (α-glucosidase from *Saccharomyces cerevisiae*, Sigma-Aldrich, St. Louis, MO, USA, 0.1 U/mL) in phosphate buffer saline was incubated with various concentrations of test compounds at 37 °C for 15 min. Then 1.25 mM *p*-nitrophenyl α-d-glucopyranoside was added to the mixture as a substrate. After further incubation at 37 °C for 30 min. The absorbance was measured spectrophotometrically at 405 nm. The sample solution was replaced by DMSO as a control. Acarbose was used as a positive control.

### 3.4. Molecular Docking

A molecular docking study was performed to investigate the binding mode between the compound **6j**,**k** and α-glucosidase using Autodock vina 1.1.2 (The Scripps Research Institute, La Jolla, CA., USA). The 3D structure of the compounds were obtained by ChemBioDraw Ultra 14.0 and ChemBio3D Ultra 14.0 softwares (CambridgeSoft, Cambridge, MA, USA). The 3D structure of α-glucosidase of *Saccharomyces cerevisiae* was predicted using homology modeling in our previous report [21]. The AutoDockTools 1.5.6 package was employed to generate the docking input files. The search grid of α-glucosidase was identified as center_x: −19.676, center_y: −7.243, and center_z: −21.469 with dimensions size_x: 15, size_y: 15, and size_z: 15. The value of exhaustiveness was set to 20. For Vina docking, the default parameters were used if it was not mentioned. The best-scoring pose as judged by the Vina docking score was chosen and visually analyzed using PyMoL 1.7.6 software (Schrödinger^®^, New York, NY, USA, http://www.pymol.org/).

## 4. Conclusions

In this paper, we have reported the synthesis of a novel series of 2-substituted-4,6-diarylpyrimidine derivatives, which were studied for their in vitro α-glucosidase inhibitory activity. The majority of the synthesized compounds possessed significant α-glucosidase inhibitory activity and therefore may potentially be developed as new α-glucosidase inhibitors for the treatment of type 2 diabetes.

## Figures and Tables

**Table 1:**
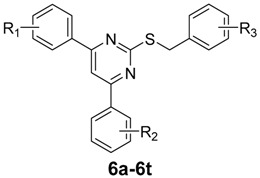
α-Glucosidase inhibitory activity of 2-substituted-4,6-diarylpyrimidines **6a**–**6t**.

Compound	R_1_	R_2_	R_3_	IC_50_ (μM)
**6a**	4-Br	4-Br	2-Cl	20.4 ± 0.23
**6b**	4-Br	4-Br	4-Cl	23.7 ± 0.27
**6c**	4-Br	4-Br	3-F	37.6 ± 0.34
**6d**	4-Br	4-Br	2-F	29.0 ± 0.28
**6e**	4-Br	4-Br	4-F	32.0 ± 0.26
**6f**	4-Me	3-Br	2-Cl	38.8 ± 0.37
**6g**	4-Me	3-Br	3-F	38.9 ± 0.35
**6h**	4-Me	3-Br	4-Cl	>50
**6i**	4-Me	3-Br	2-F	>50
**6j**	4-Me	4-Br	4-Cl	19.6 ± 0.21
**6k**	4-Me	4-Br	2-Br	21.2 ± 0.25
**6l**	4-Me	4-Br	4-F	>50
**6m**	4-Me	4-Br	2-F	>50
**6n**	4-Me	4-Br	3-F	>50
**6o**	4-Me	4-Br	H	>50
**6p**	4-Me	4-Br	2,4-Cl_2_	>50
**6q**	4-Me	3-Br	4-F	32.1 ± 0.34
**6r**	4-Me	3-Br	4-Br	>50
**6s**	3-Br	4-Br	2-Cl	26.2 ± 0.27
**6t**	3-Br	4-Br	3-F	25.3 ± 0.28
**Acarbose**				817.38 ± 6.27

## Supplementary Materials

The following are available online.
