# Supplementary material for: Synthesis, Biological Evaluation and Molecular Docking Study of 2-Substituted-4,6-Diarylpyrimidines as α-Glucosidase Inhibitors
Source: Molecules. 2017 Oct 30;22(11):1865. doi: 10.3390/molecules22111865 (PMC6150375; doi:10.3390/molecules22111865)
Supplement: Supplementary file 1 [file molecules-22-01865-s001.pdf]

Supplemental Material

Synthesis, biological evaluation and molecular docking study of 2-substituted-4,6-diarylpyrimidines as α-glucosidase inhibitors

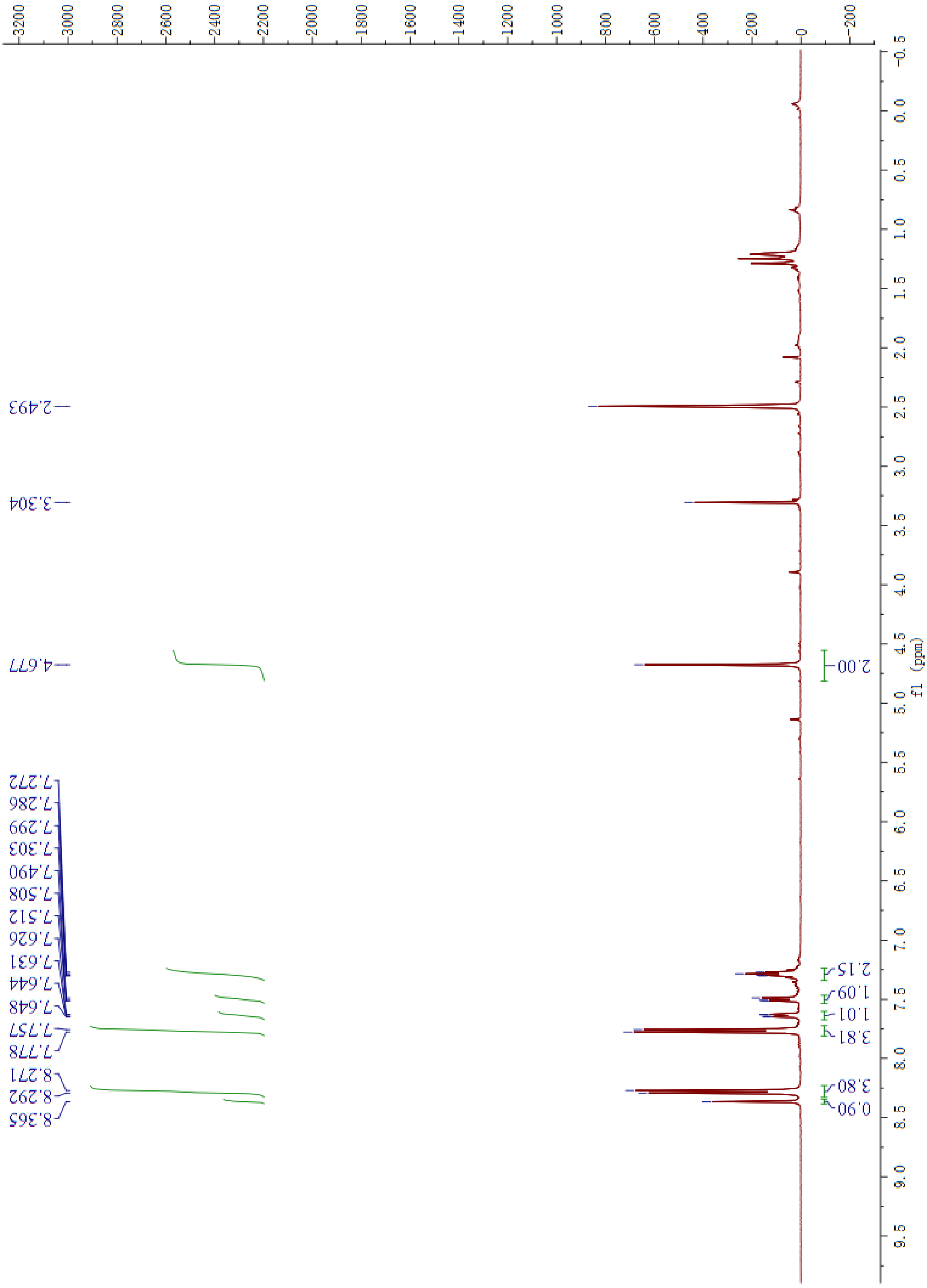

Table S1. ^{1}H NMR of compound 6a

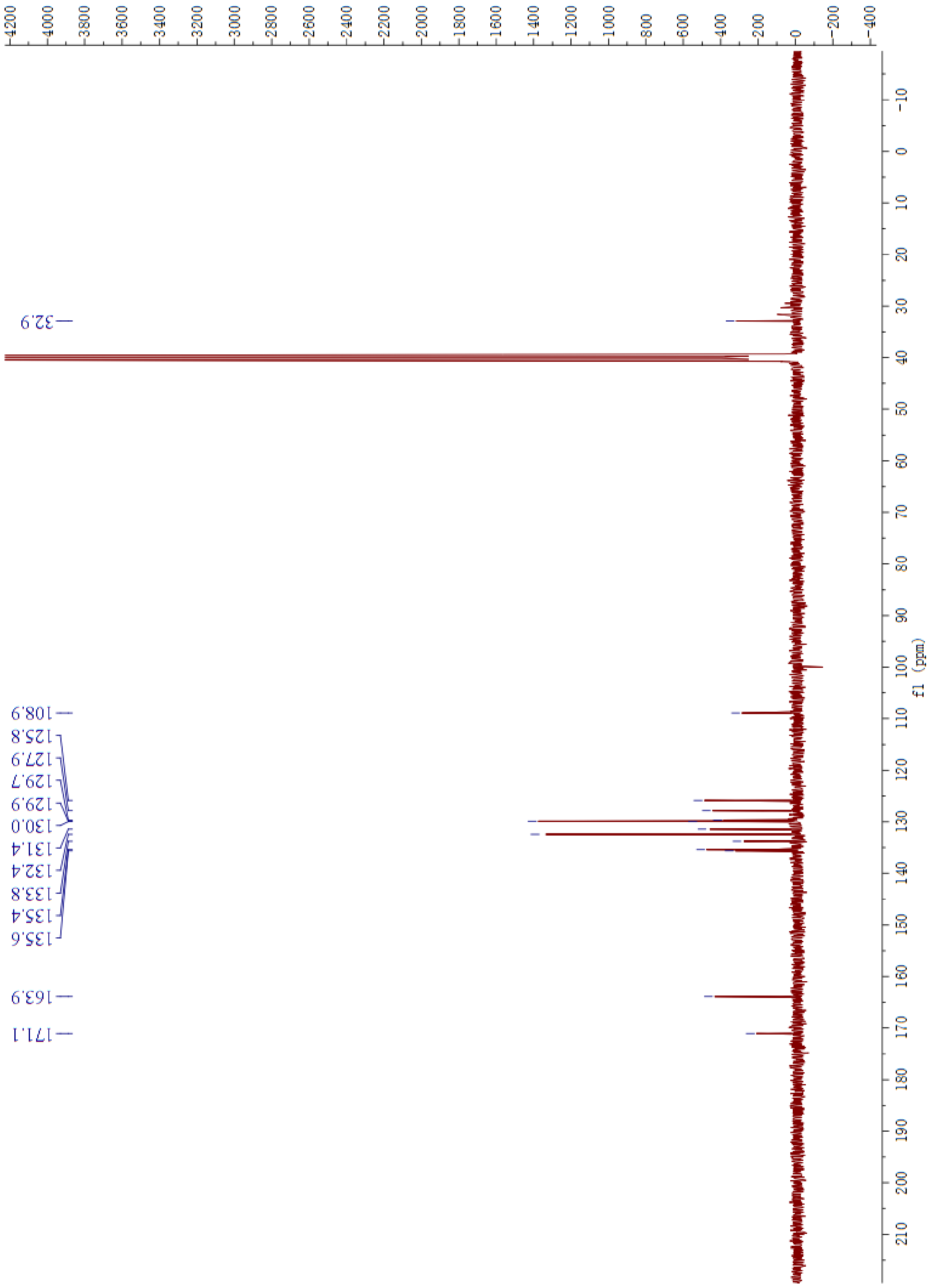

Table S2. ^{13}C NMR of compound 6a

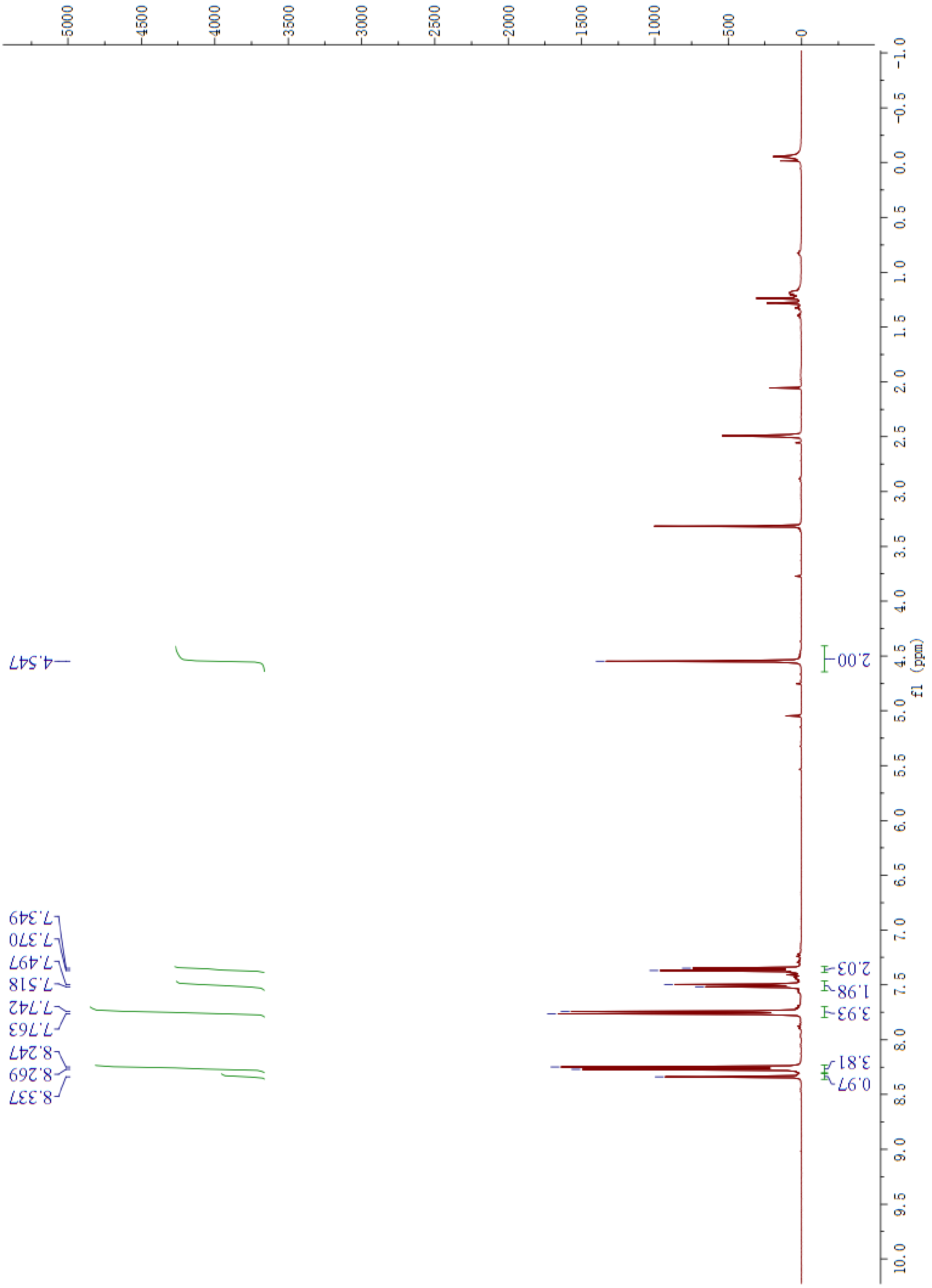

Table S3. ^{1}H NMR of compound 6b

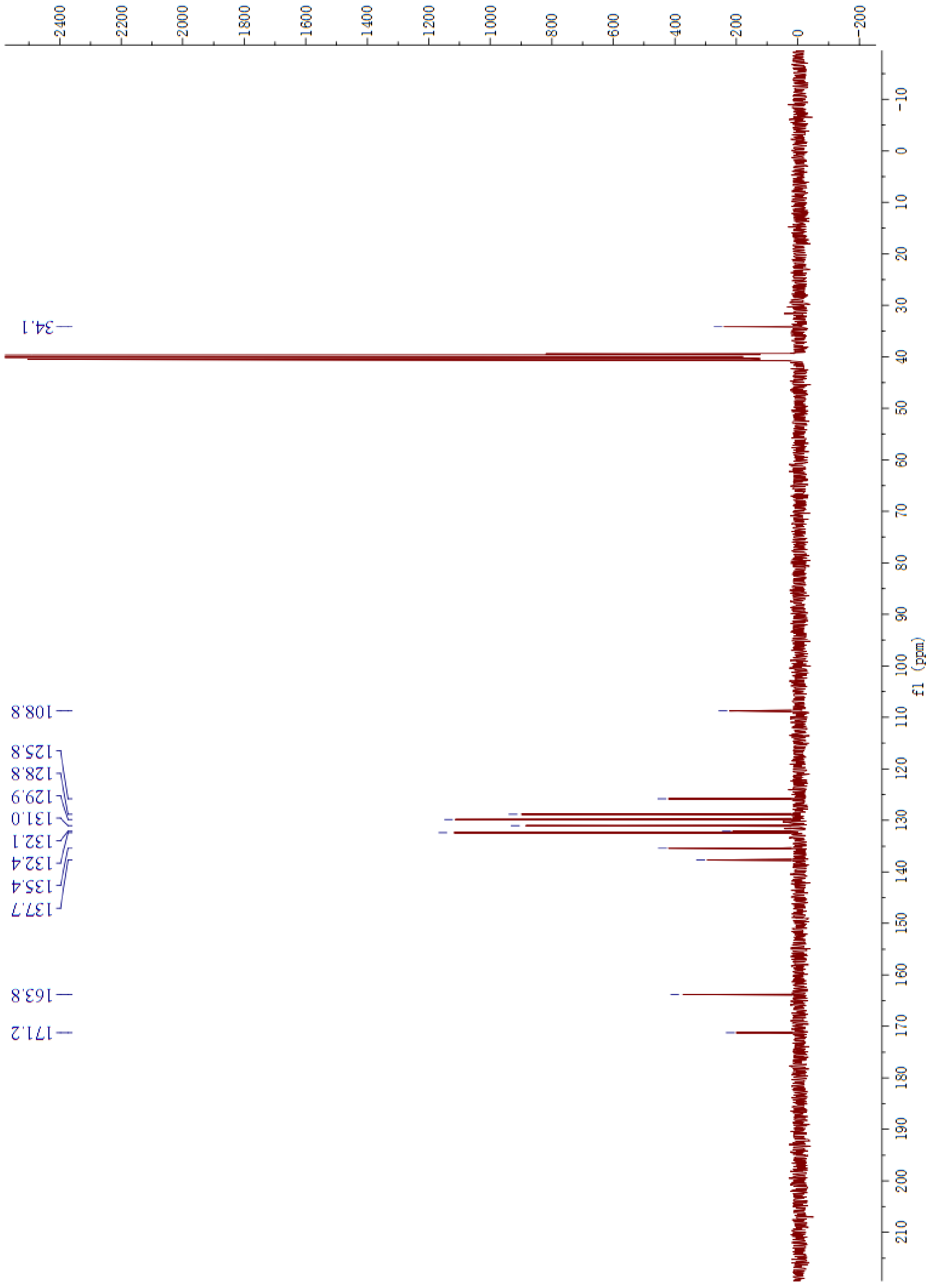

Table S4. ^{13}C NMR of compound 6b

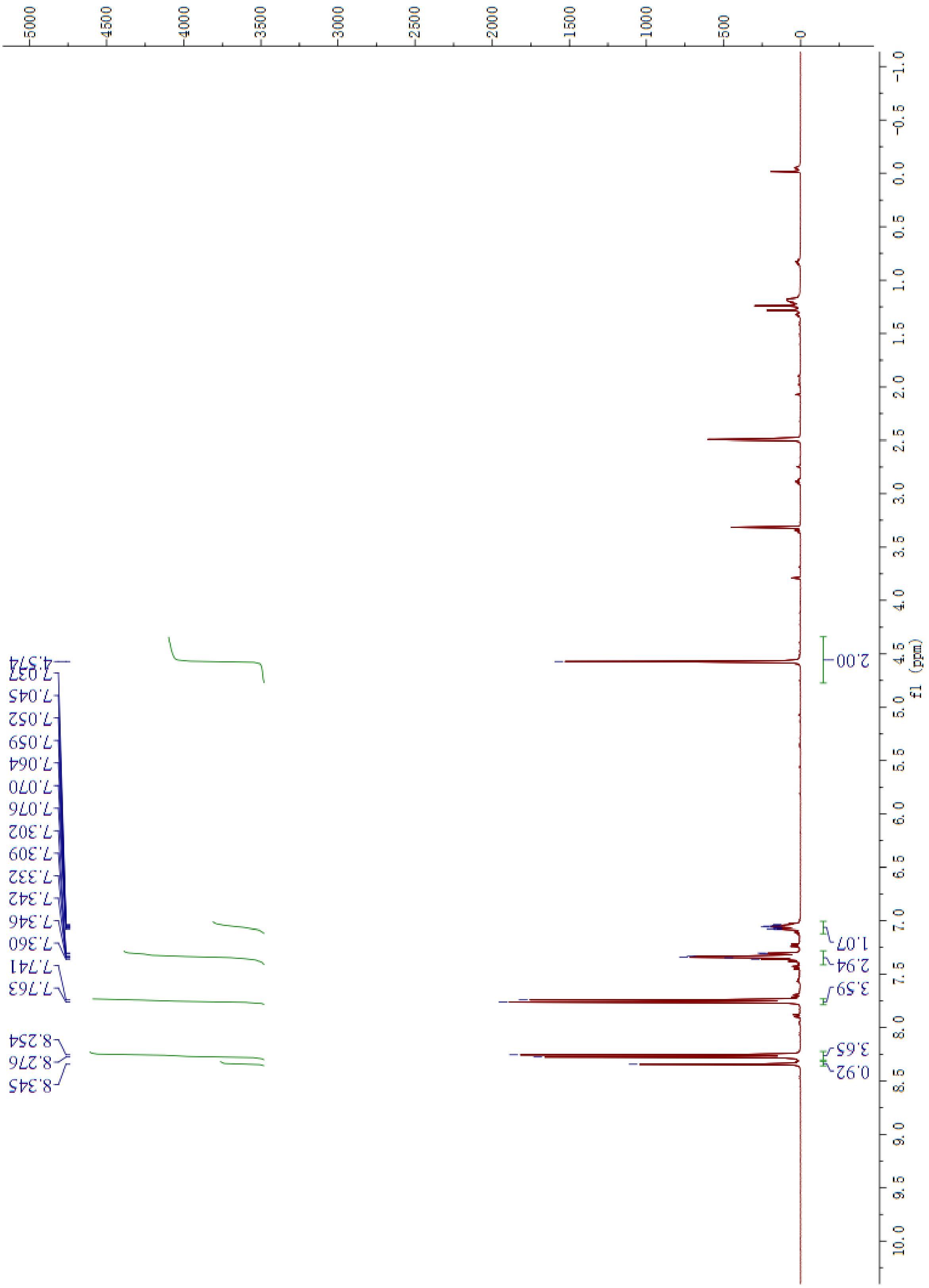

Table S5. ^{1}H NMR of compound 6c

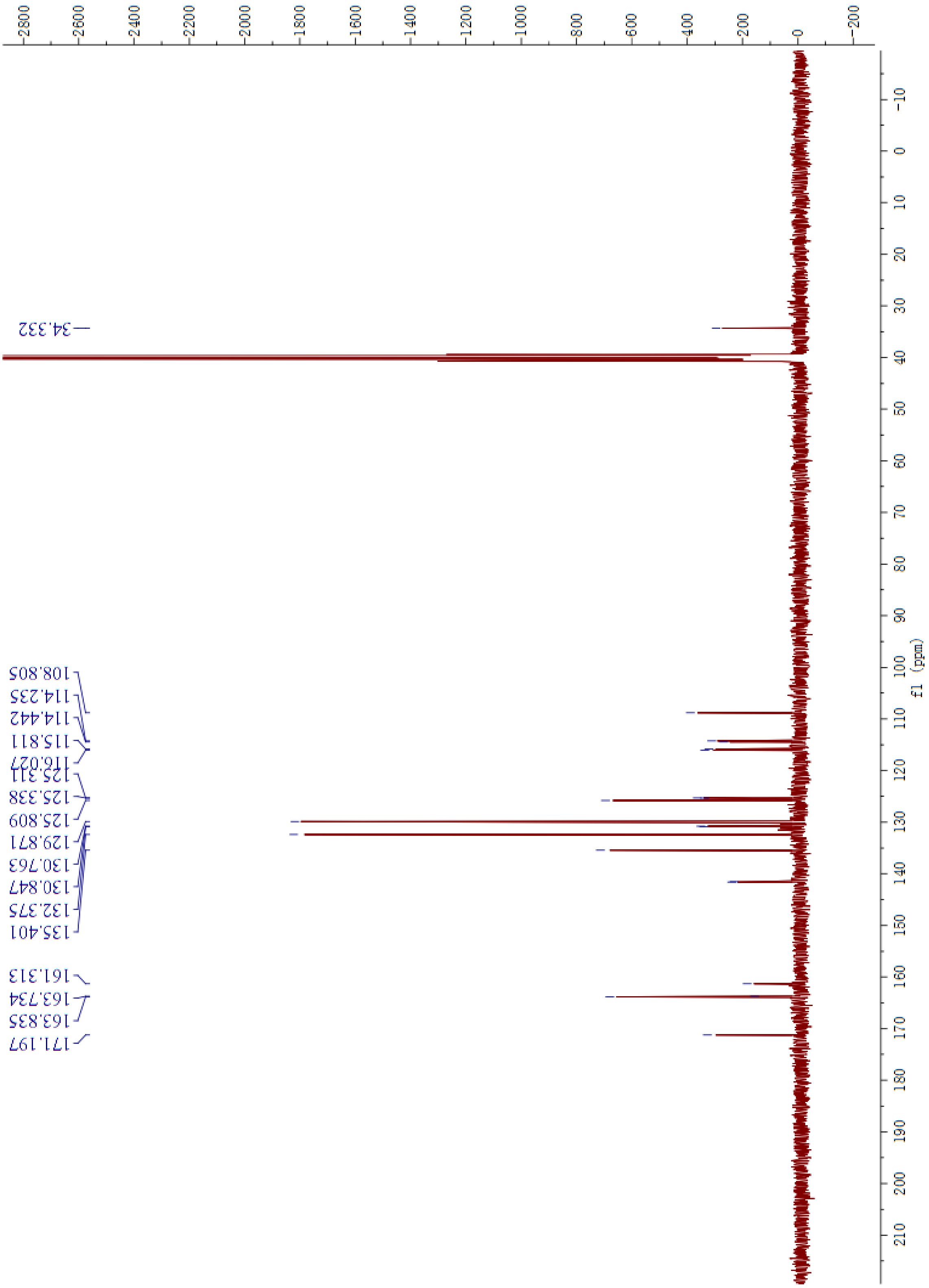

Table S6. ^{13}C NMR of compound 6c

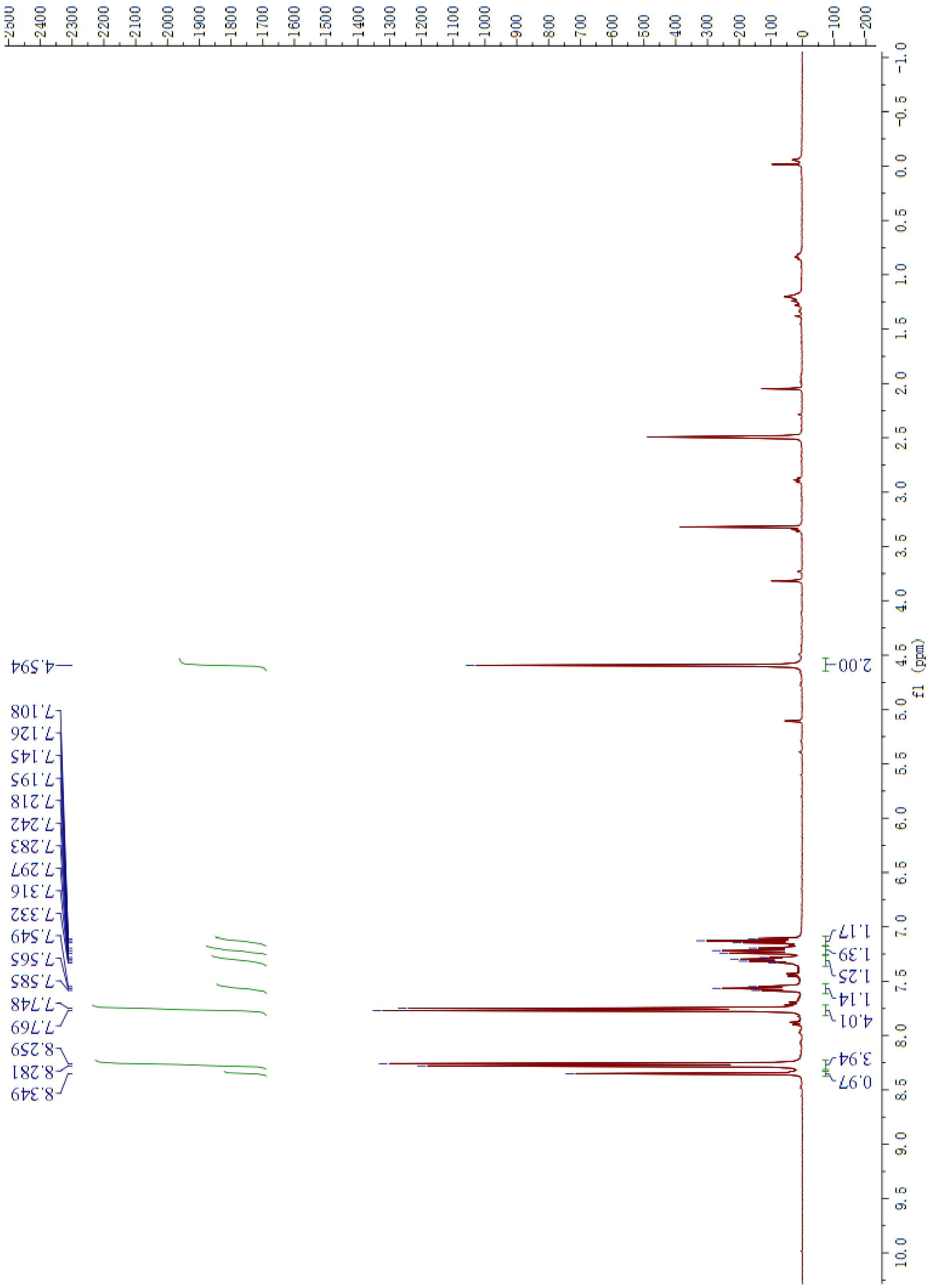

Table S7. ¹H NMR of compound 6d

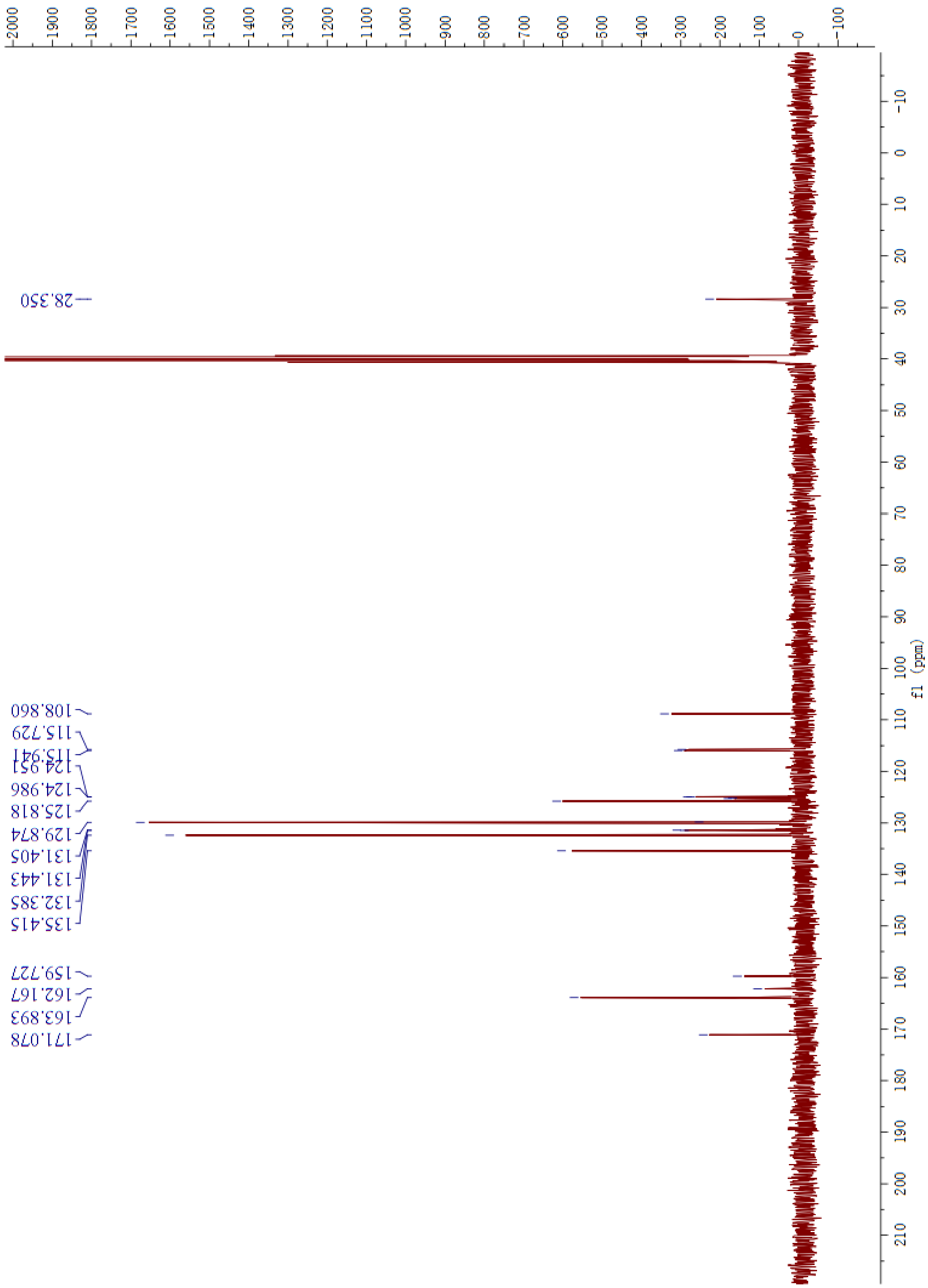

Table S8. ^{13}C NMR of compound 6d

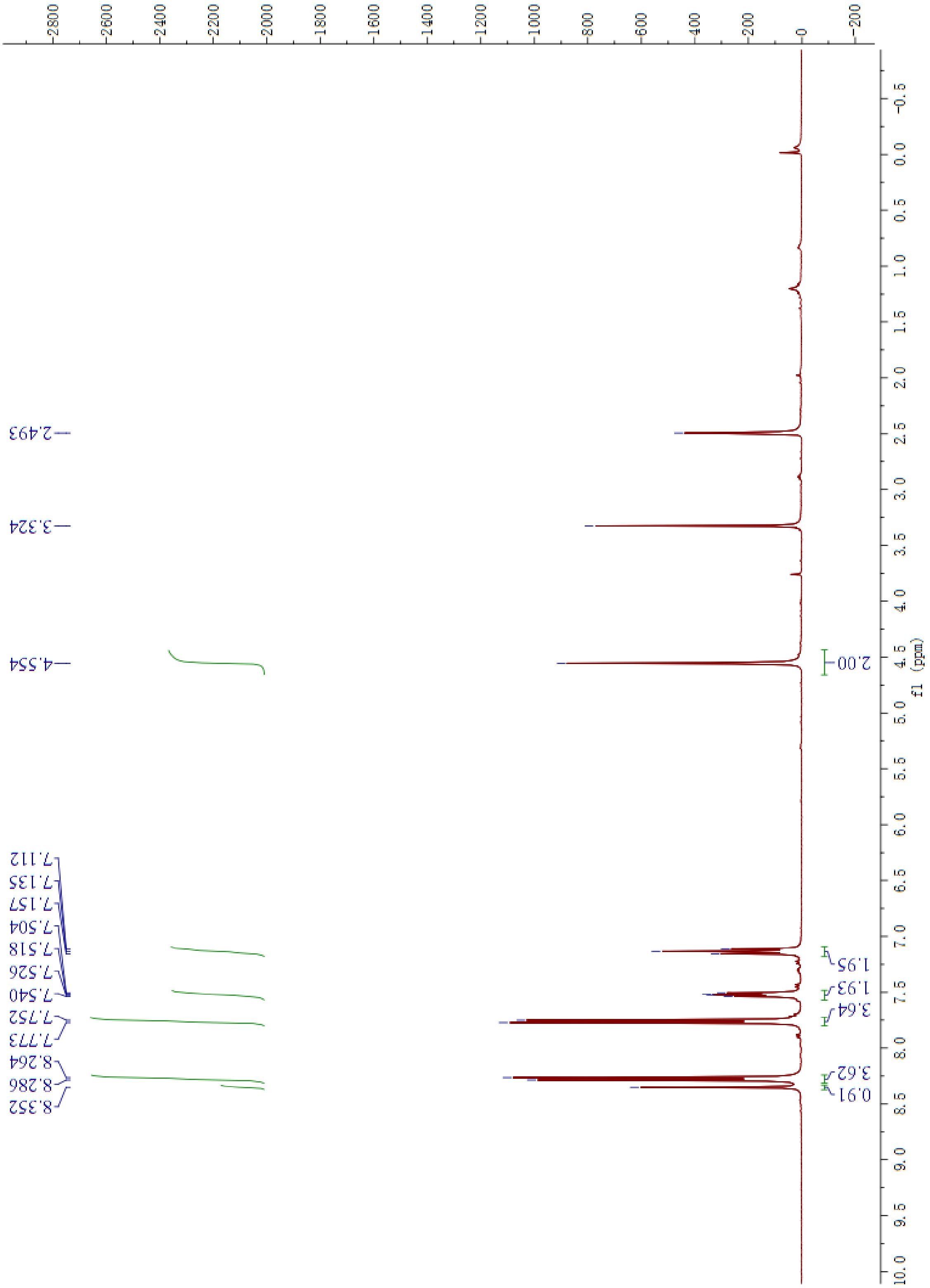

Table S9. ^{1}H NMR of compound 6e

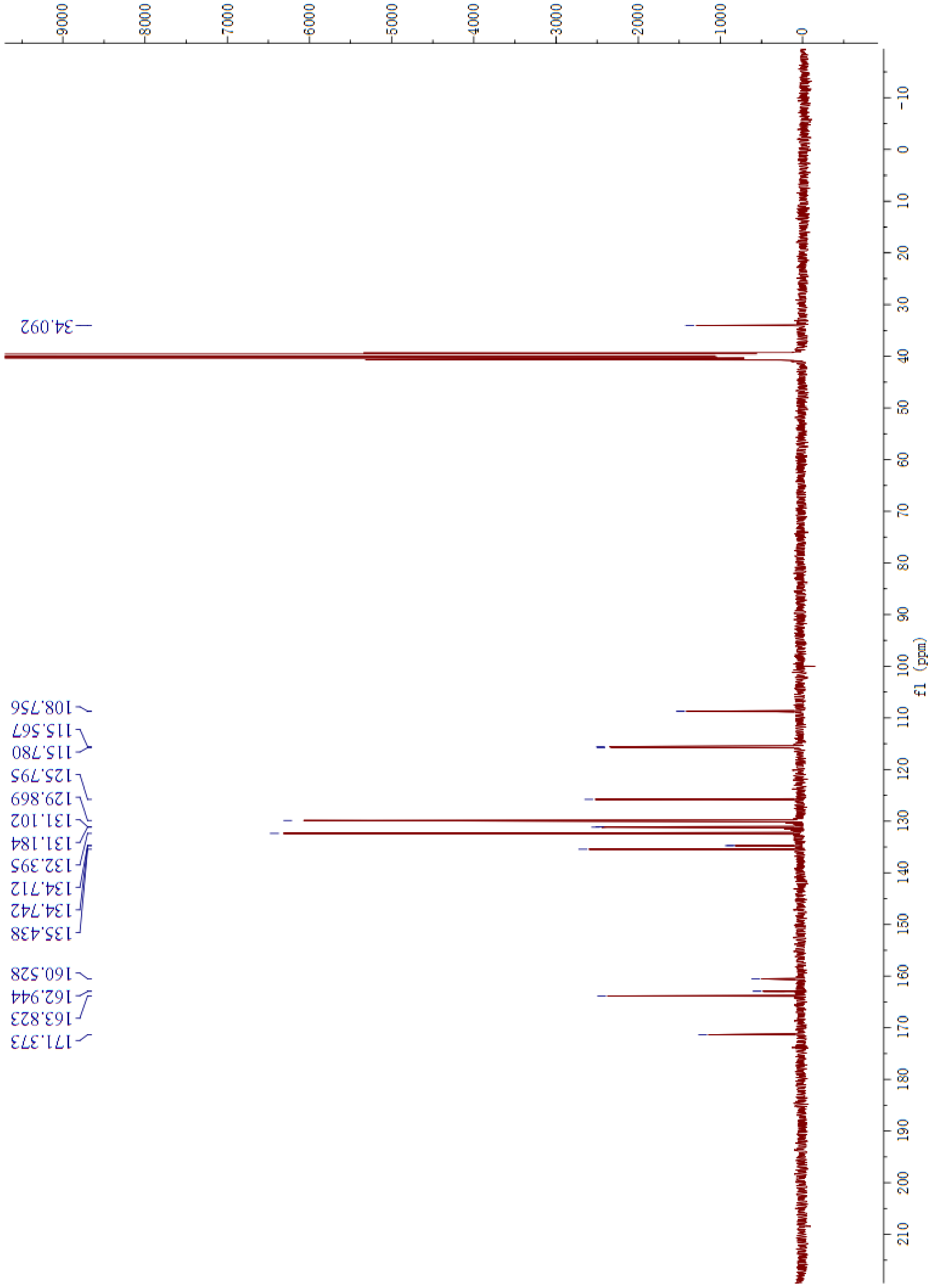

Table S10. ^{13}C NMR of compound 6e

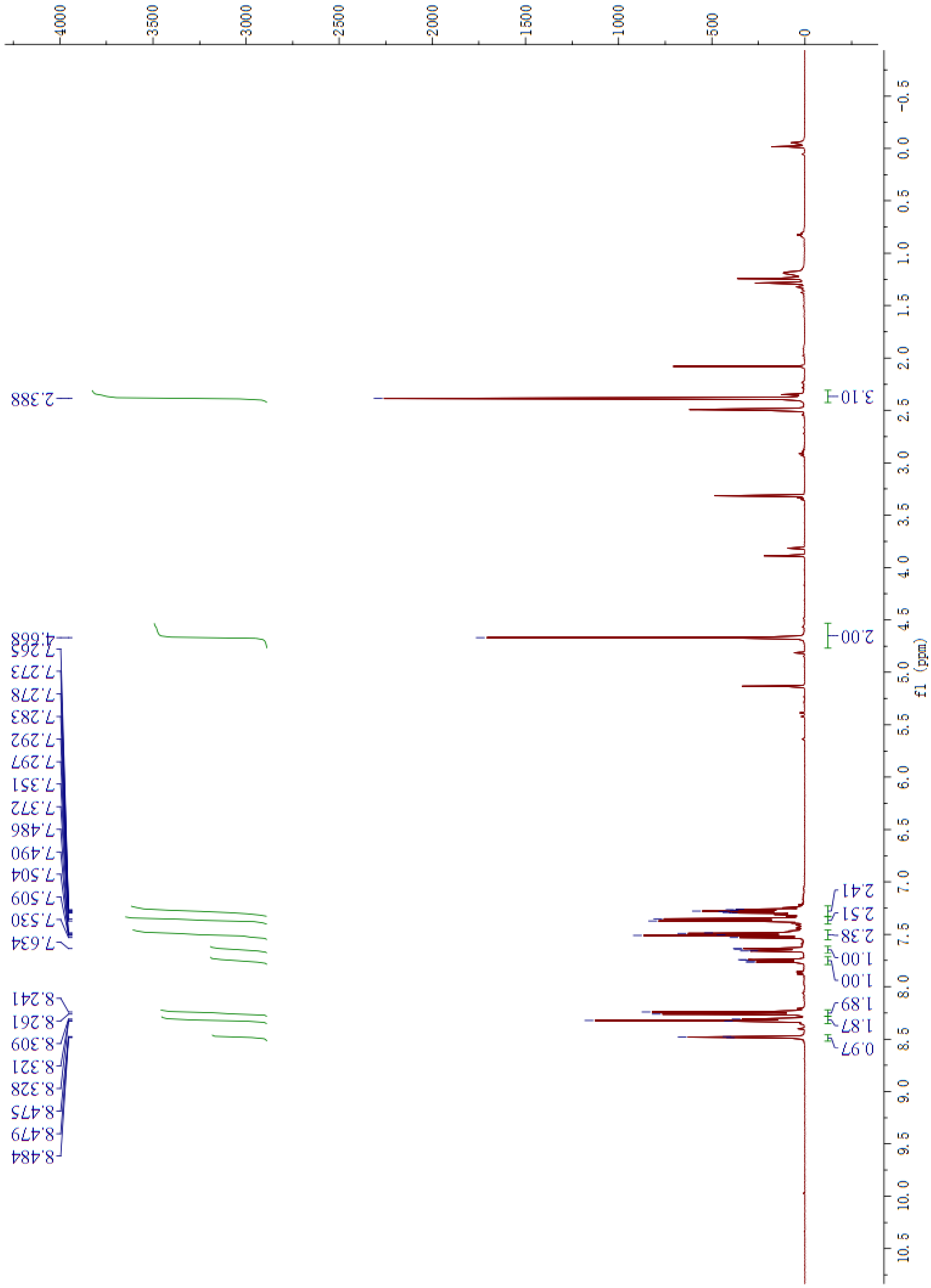

Table S11. ^{1}H NMR of compound 6f

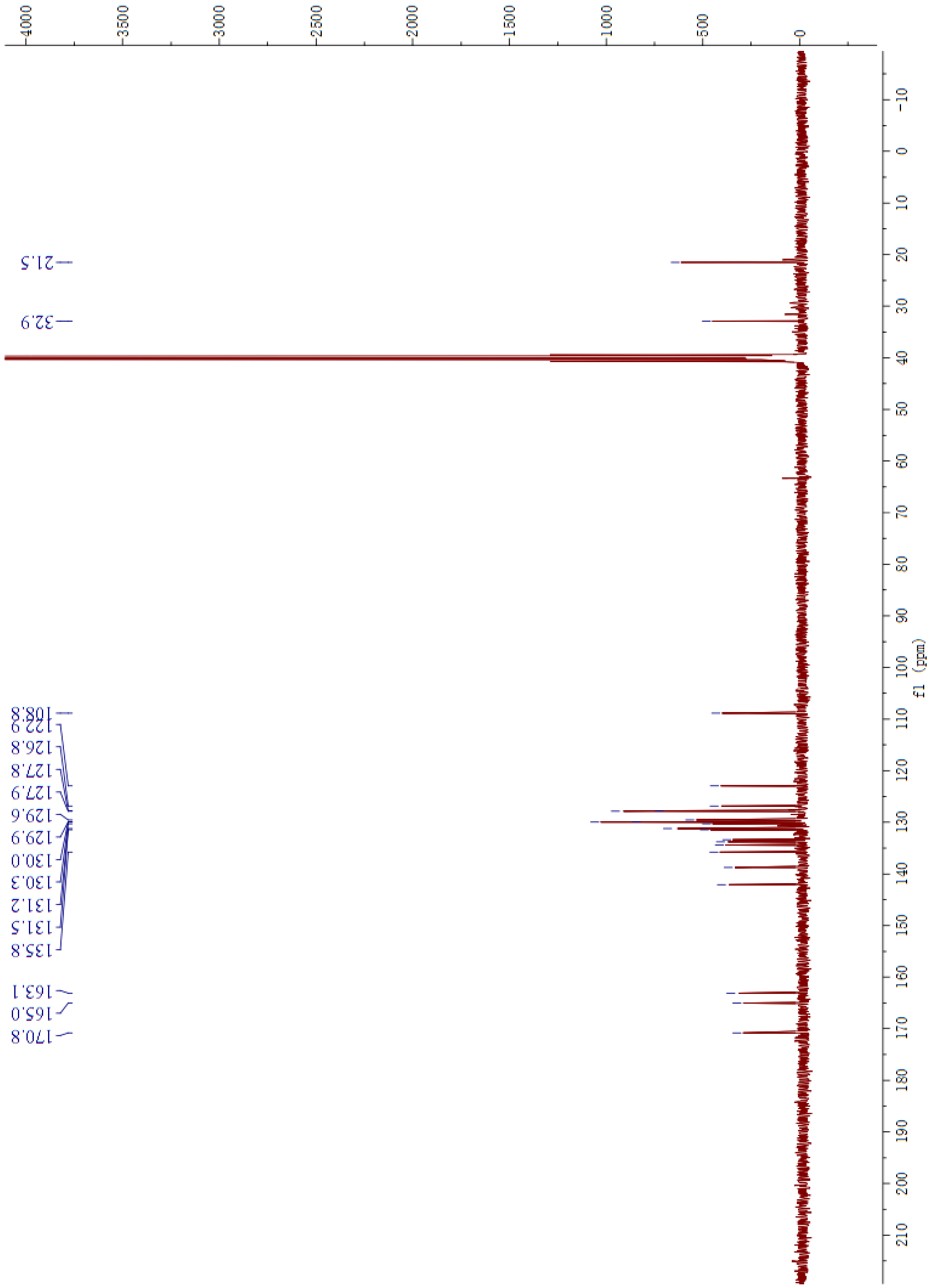

Table S12. ^{13}C NMR of compound 6f

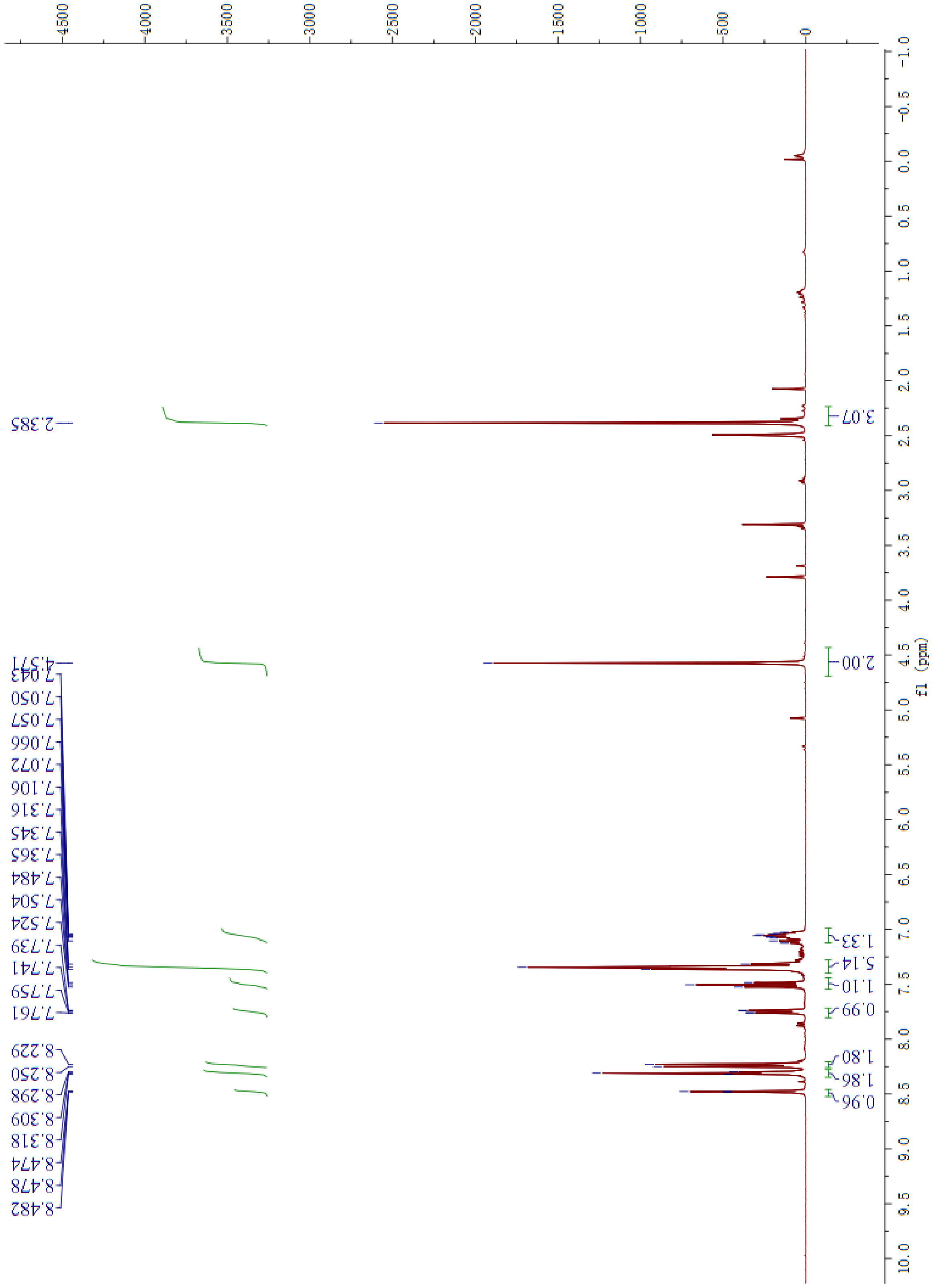

Table S13. ^{1}H NMR of compound 6g

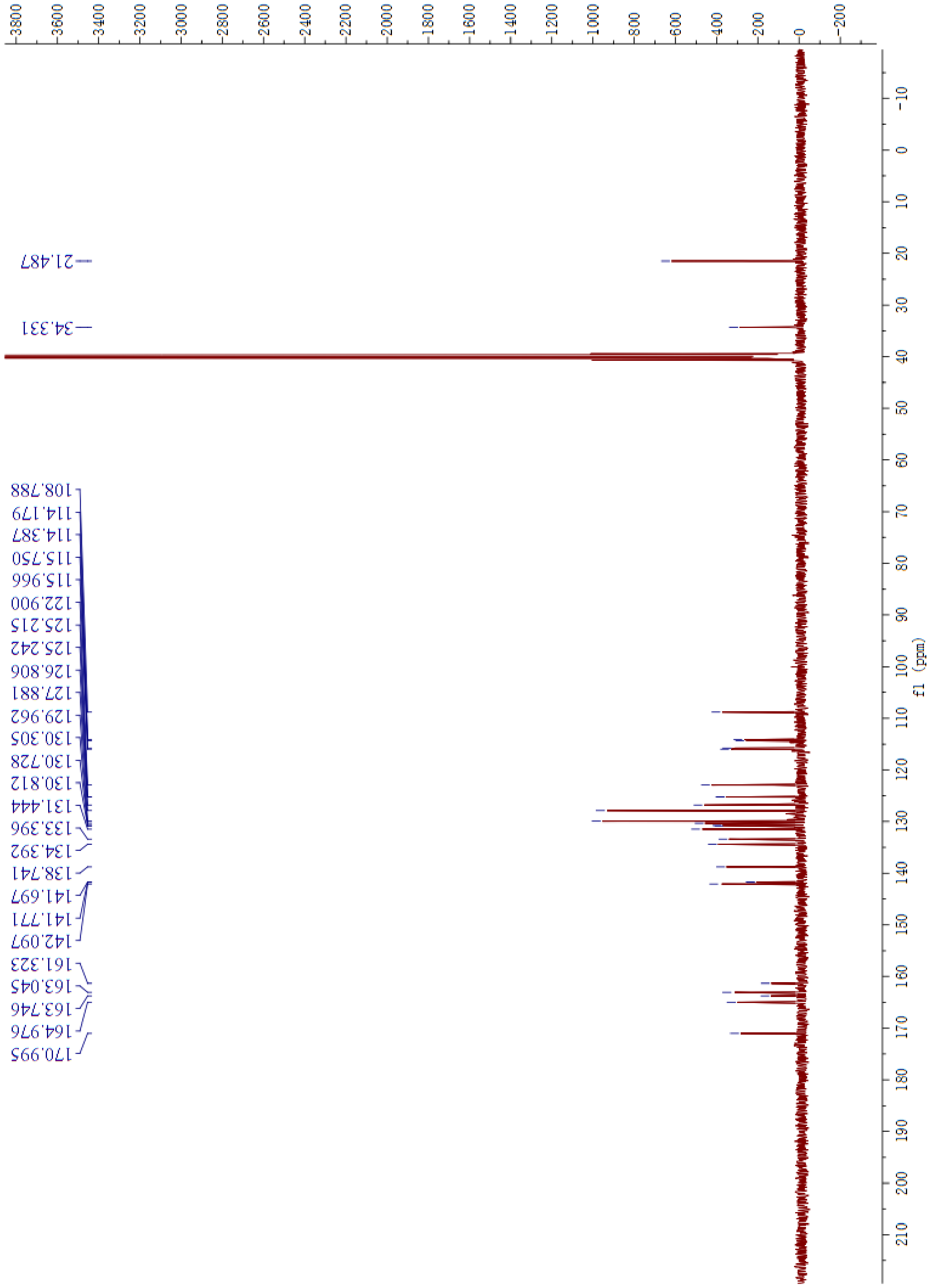

Table S14. ^{13}C NMR of compound 6g

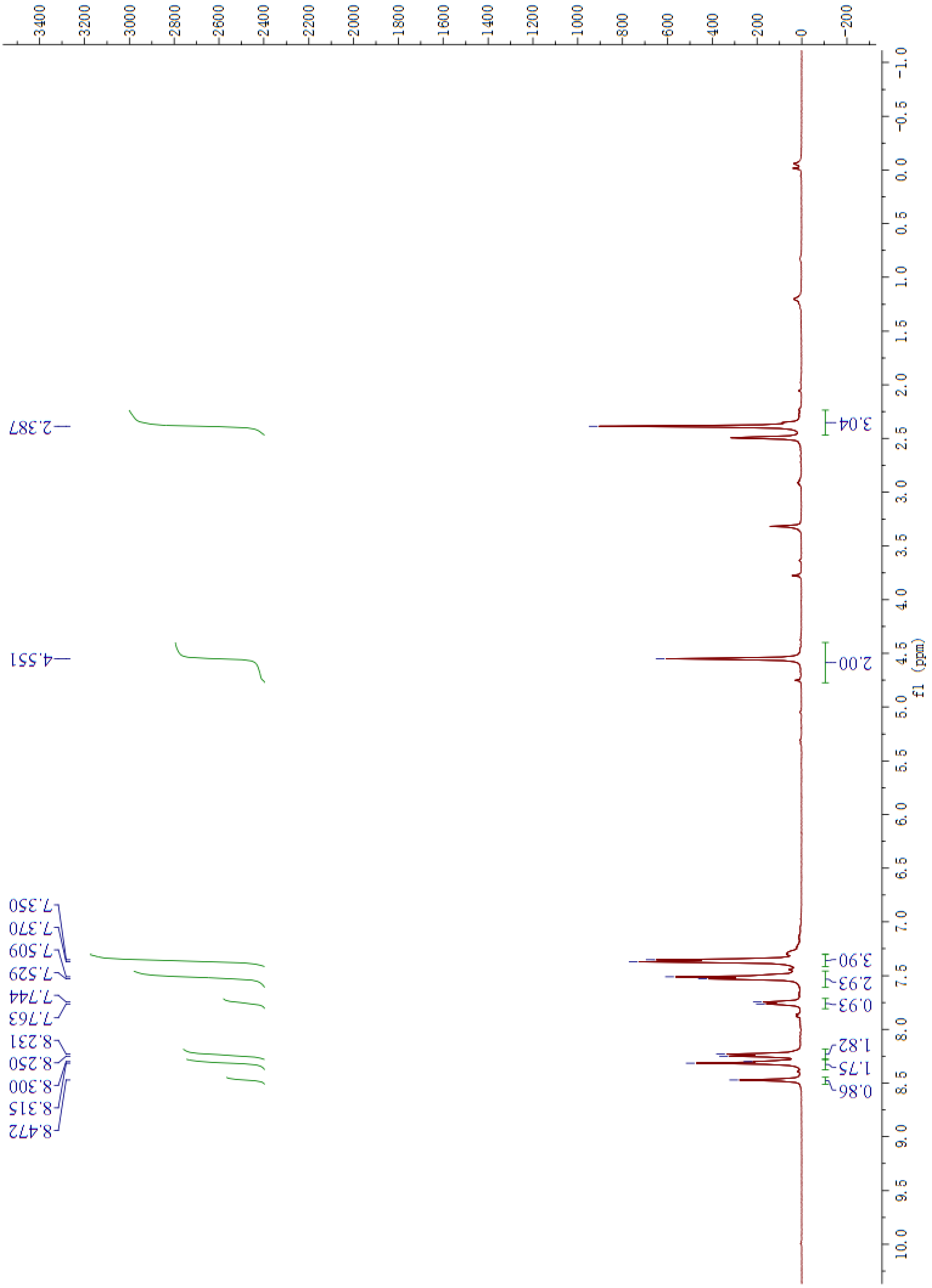

Table S15. ^{1}H NMR of compound 6h

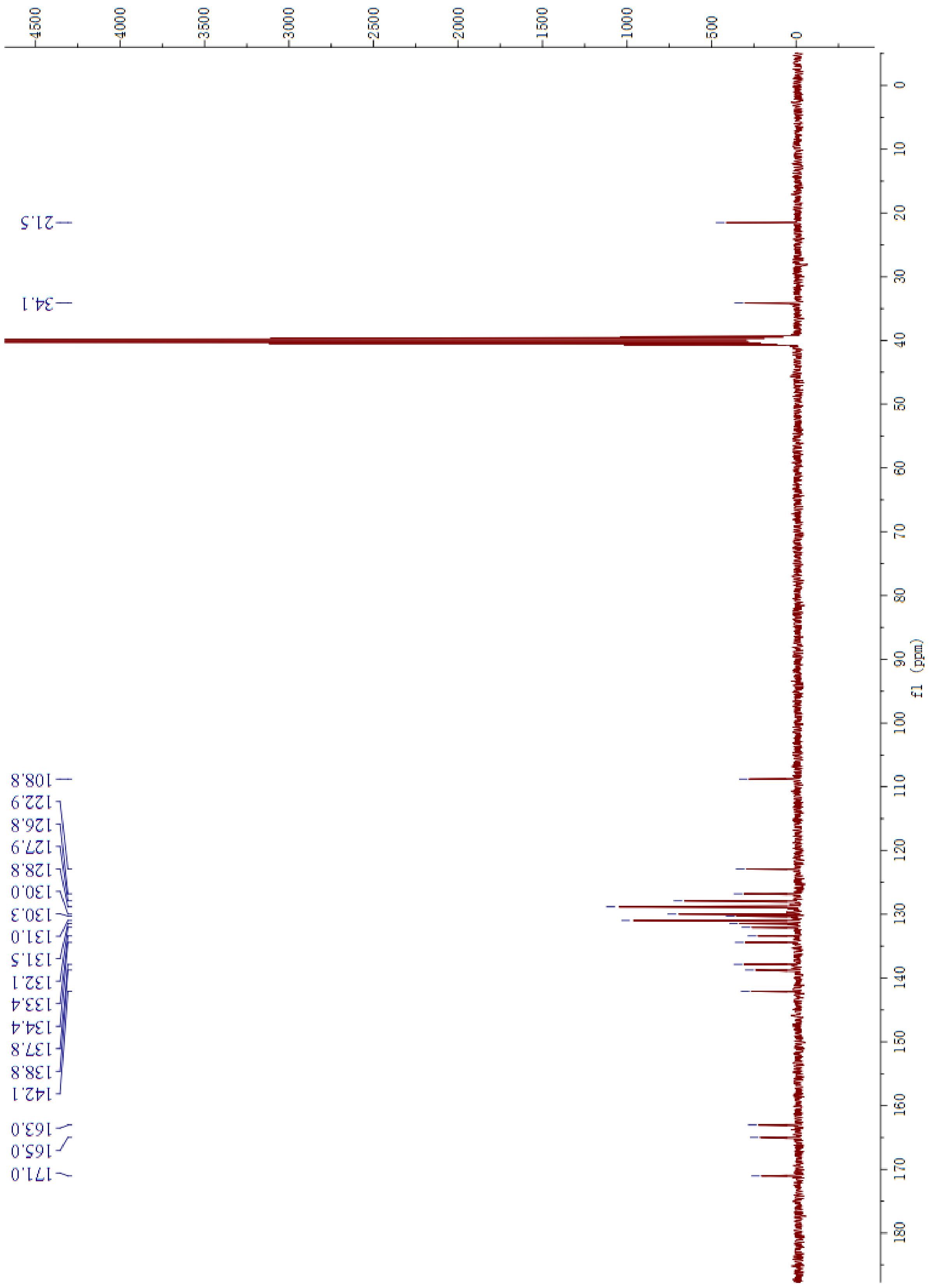

Table S16. ^{13}C NMR of compound 6h

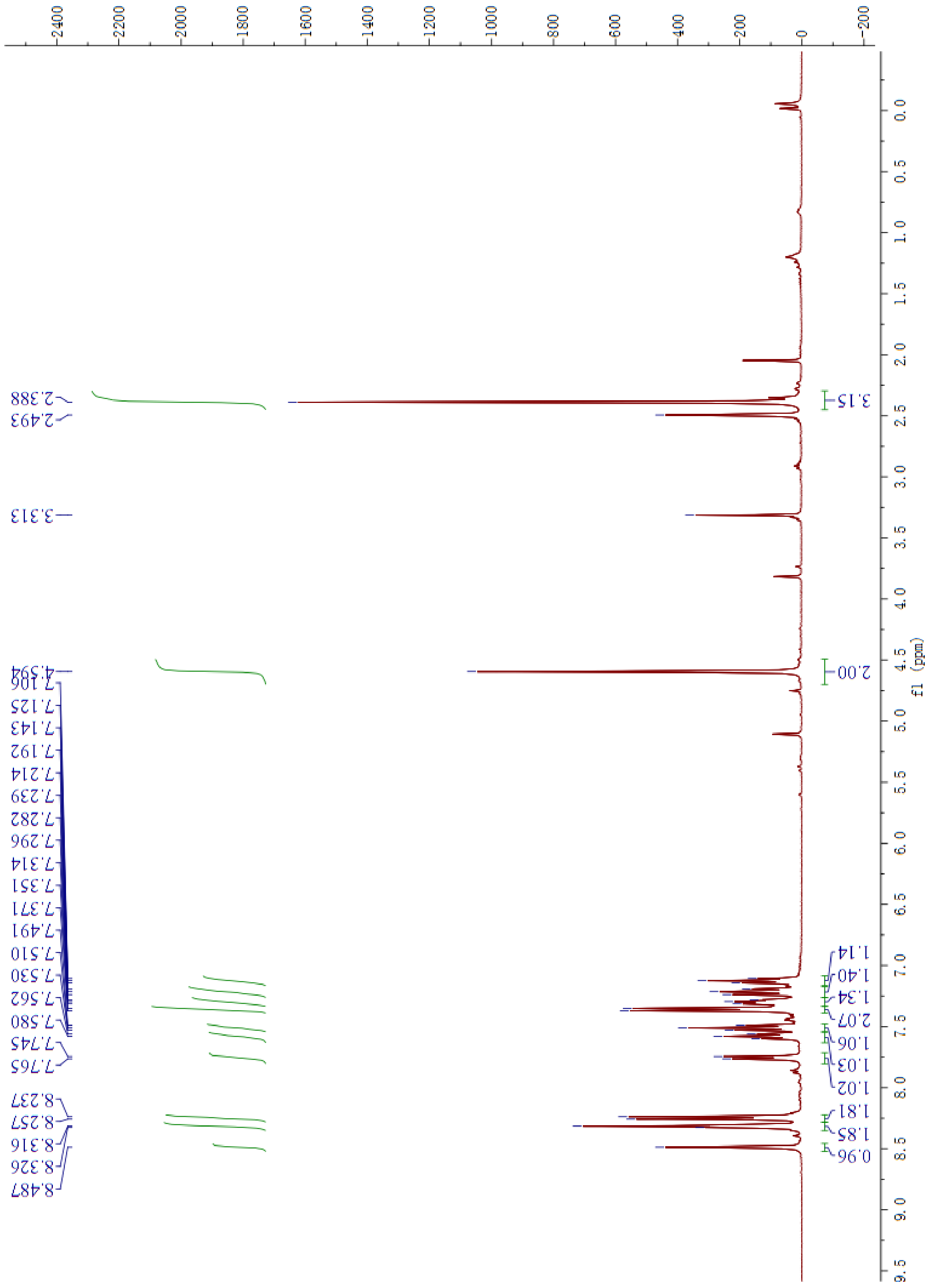

Table S17. ^{1}H NMR of compound 6i

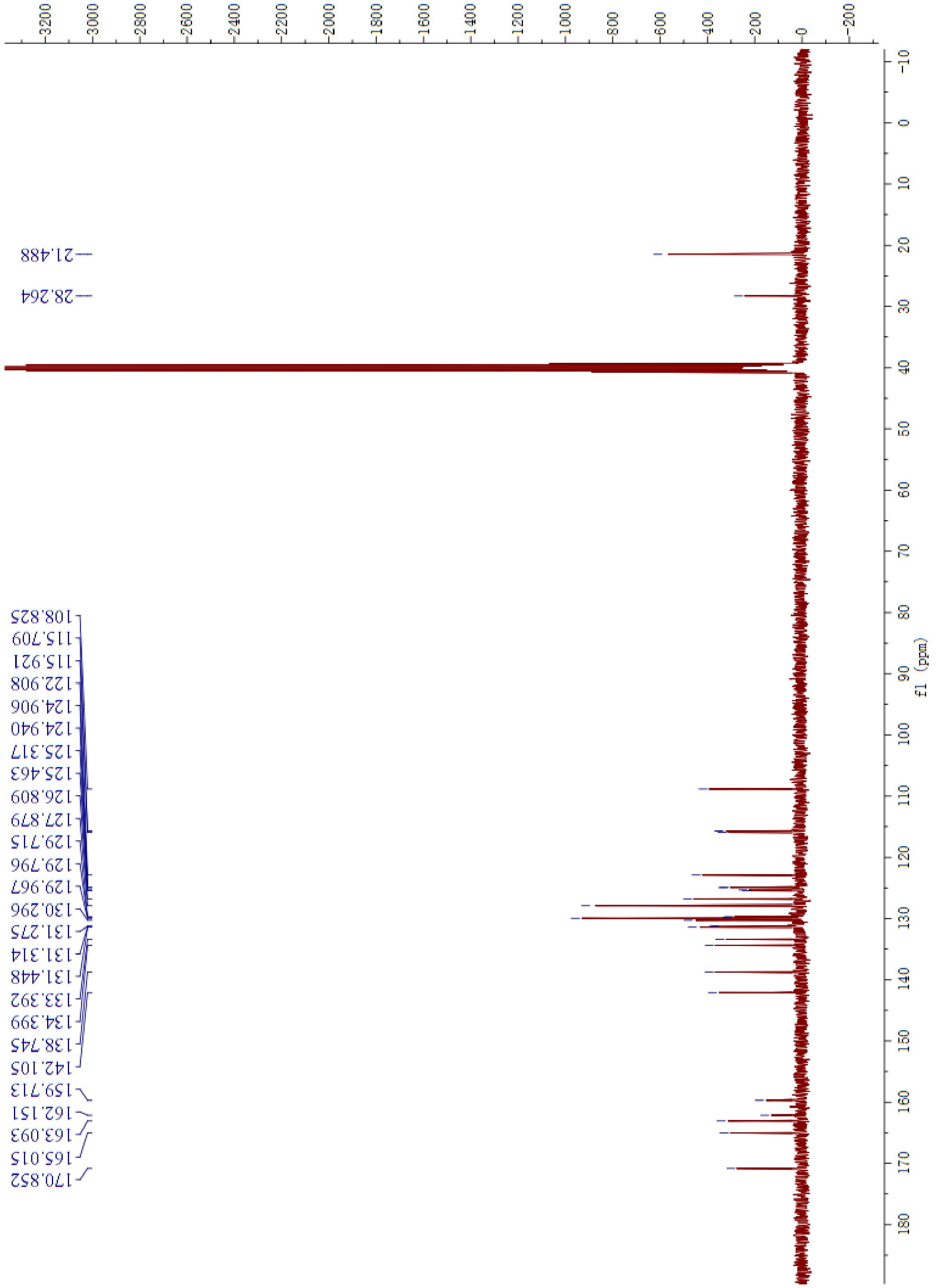

Table S18. ^{13}C NMR of compound 6i

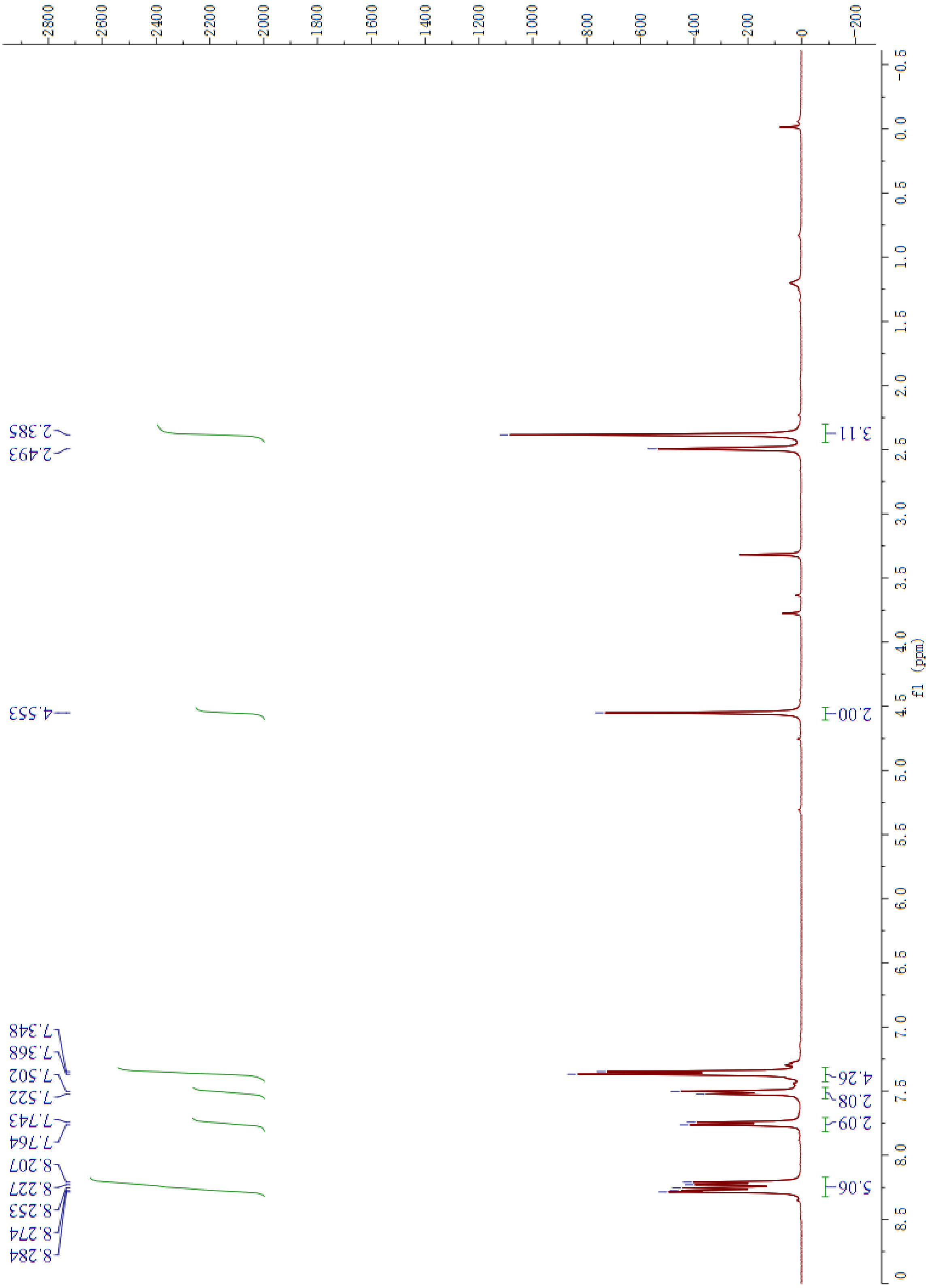

Table S19. ^{1}H NMR of compound 6j

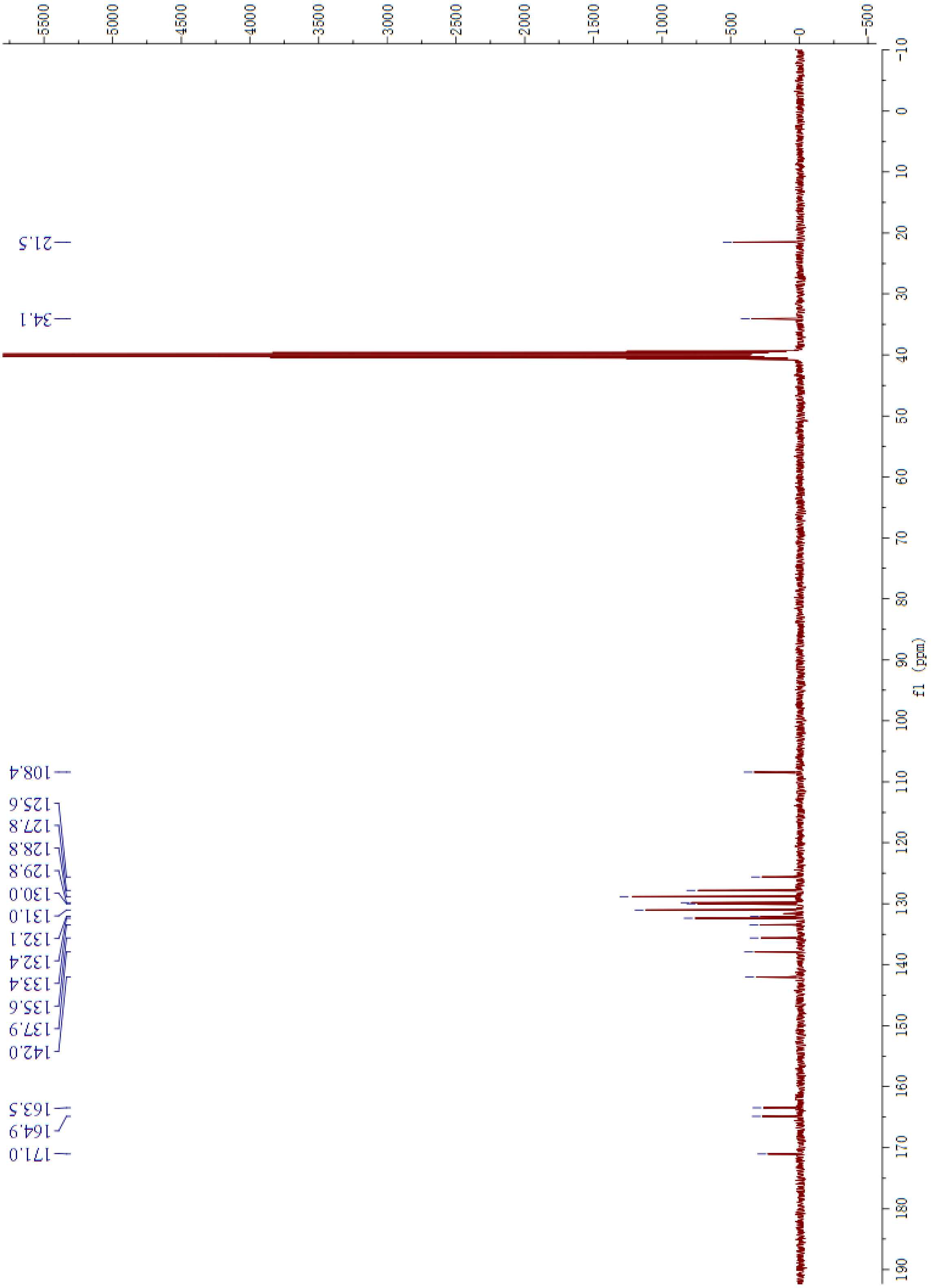

Table S20. ^{13}C NMR of compound 6j

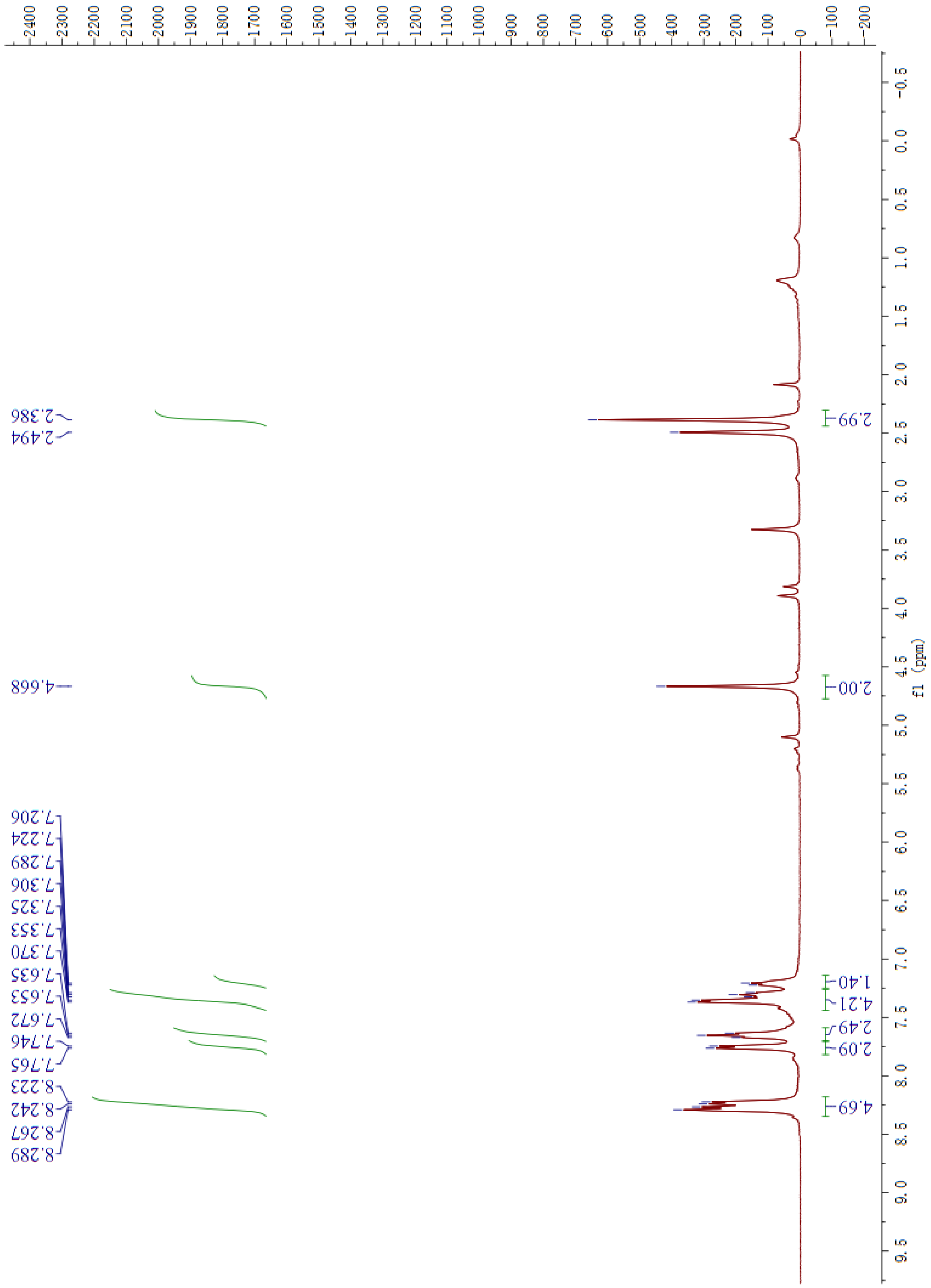

Table S21. ^{1}H NMR of compound 6k

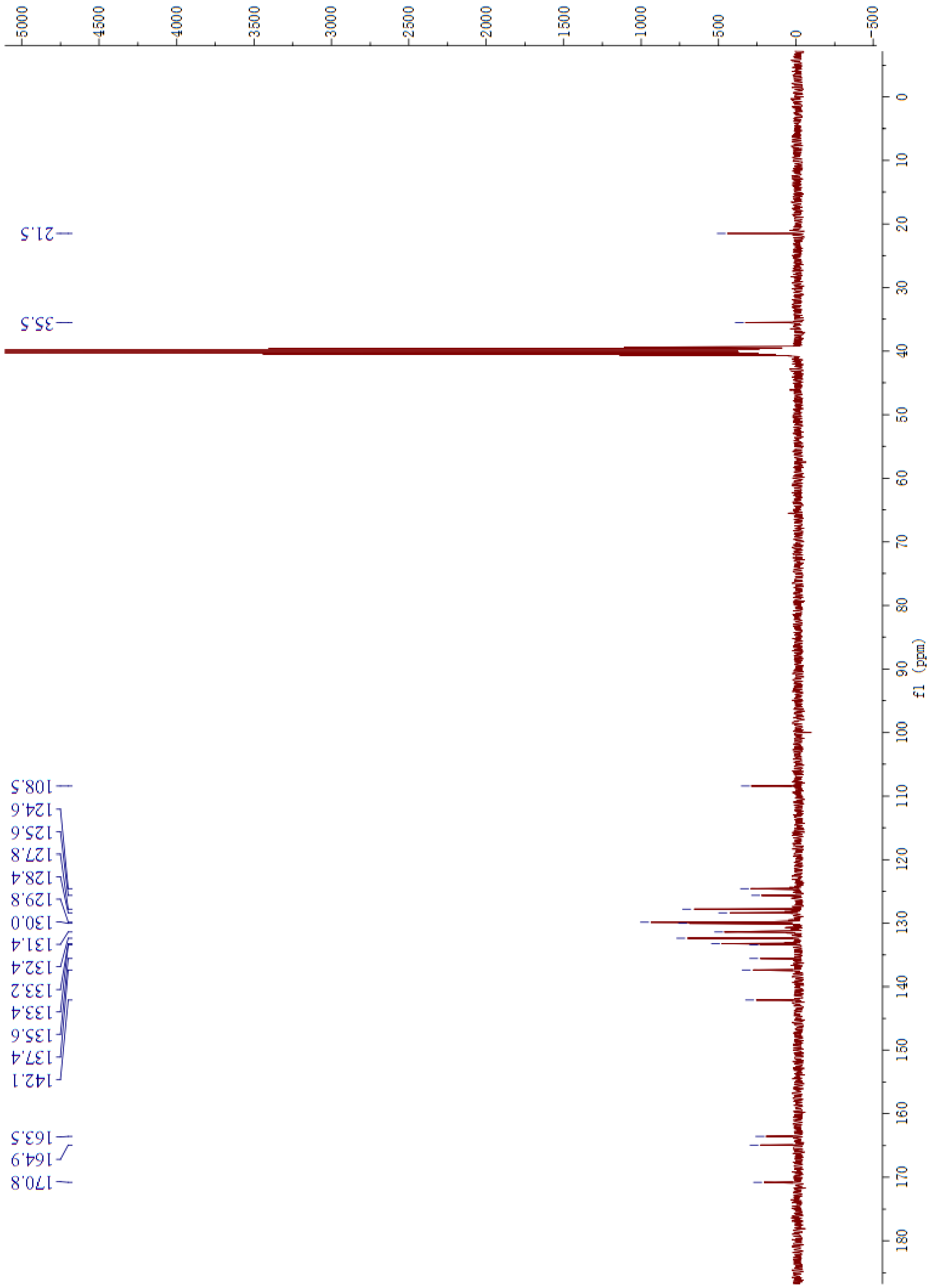

Table S22. ^{13}C NMR of compound 6k

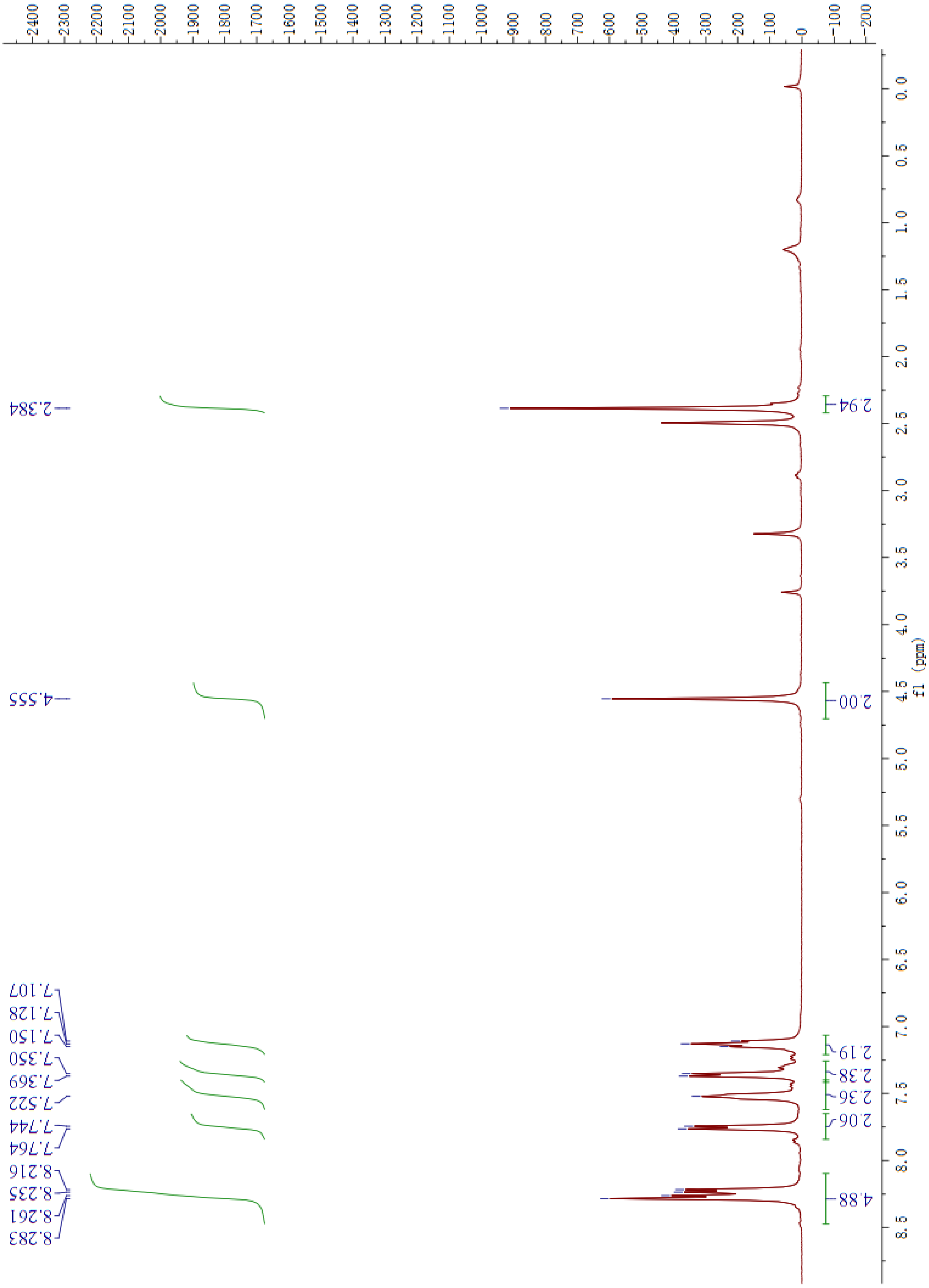

Table S23. ^{1}H NMR of compound 6l

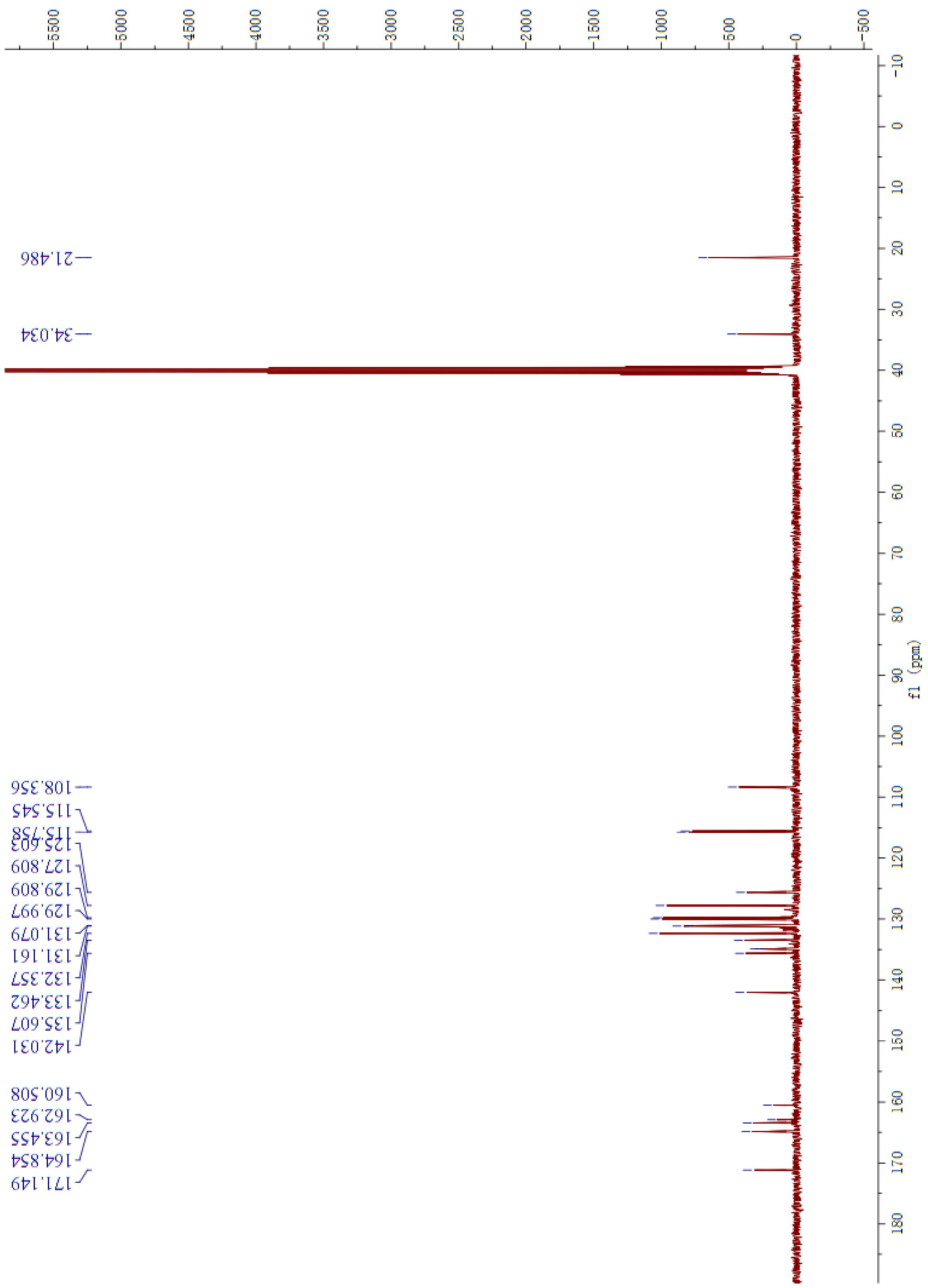

Table S24. ^{13}C NMR of compound 6l

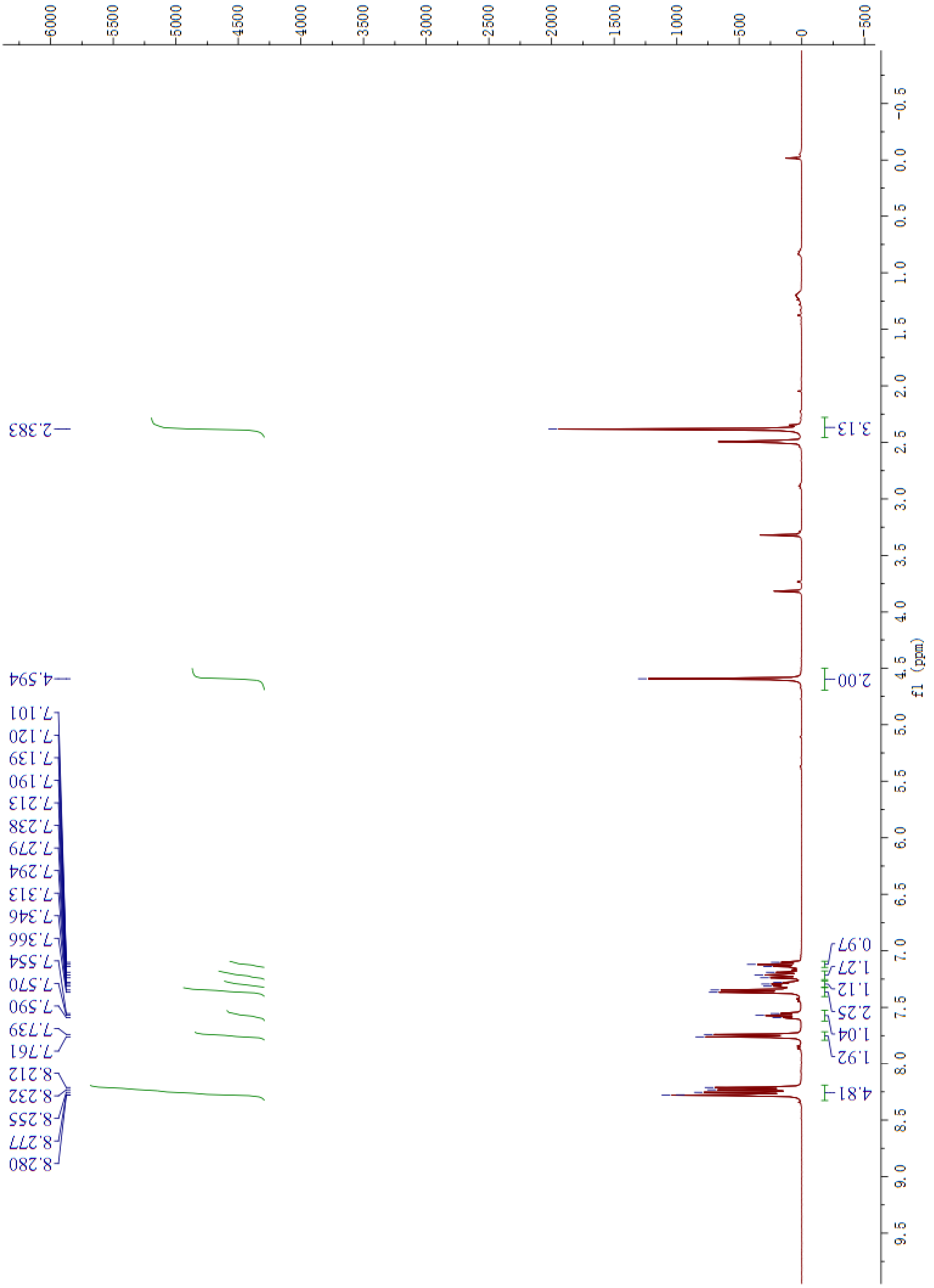

Table S25. ^{1}H NMR of compound 6m

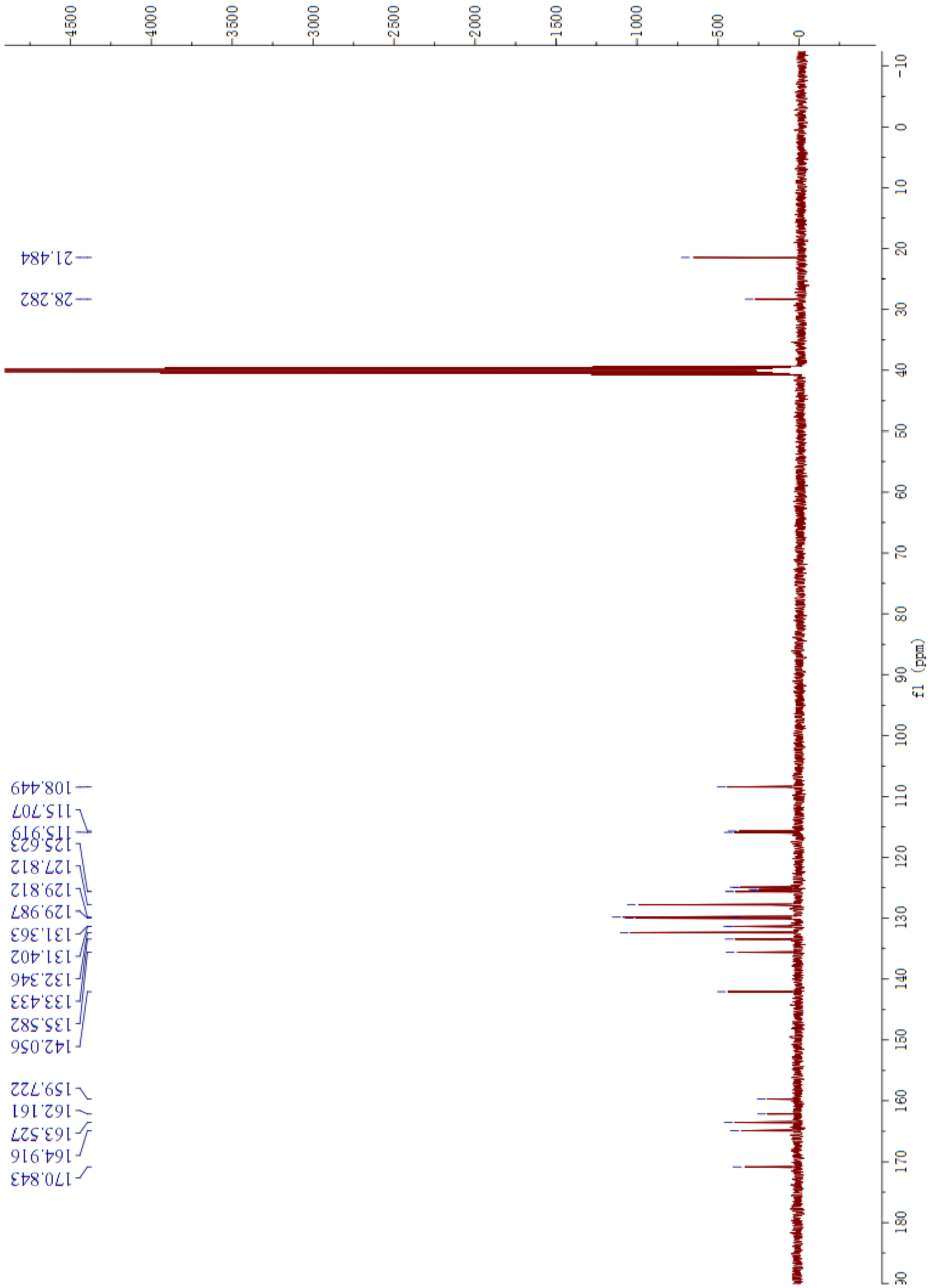

Table S26. ^{13}C NMR of compound 6m

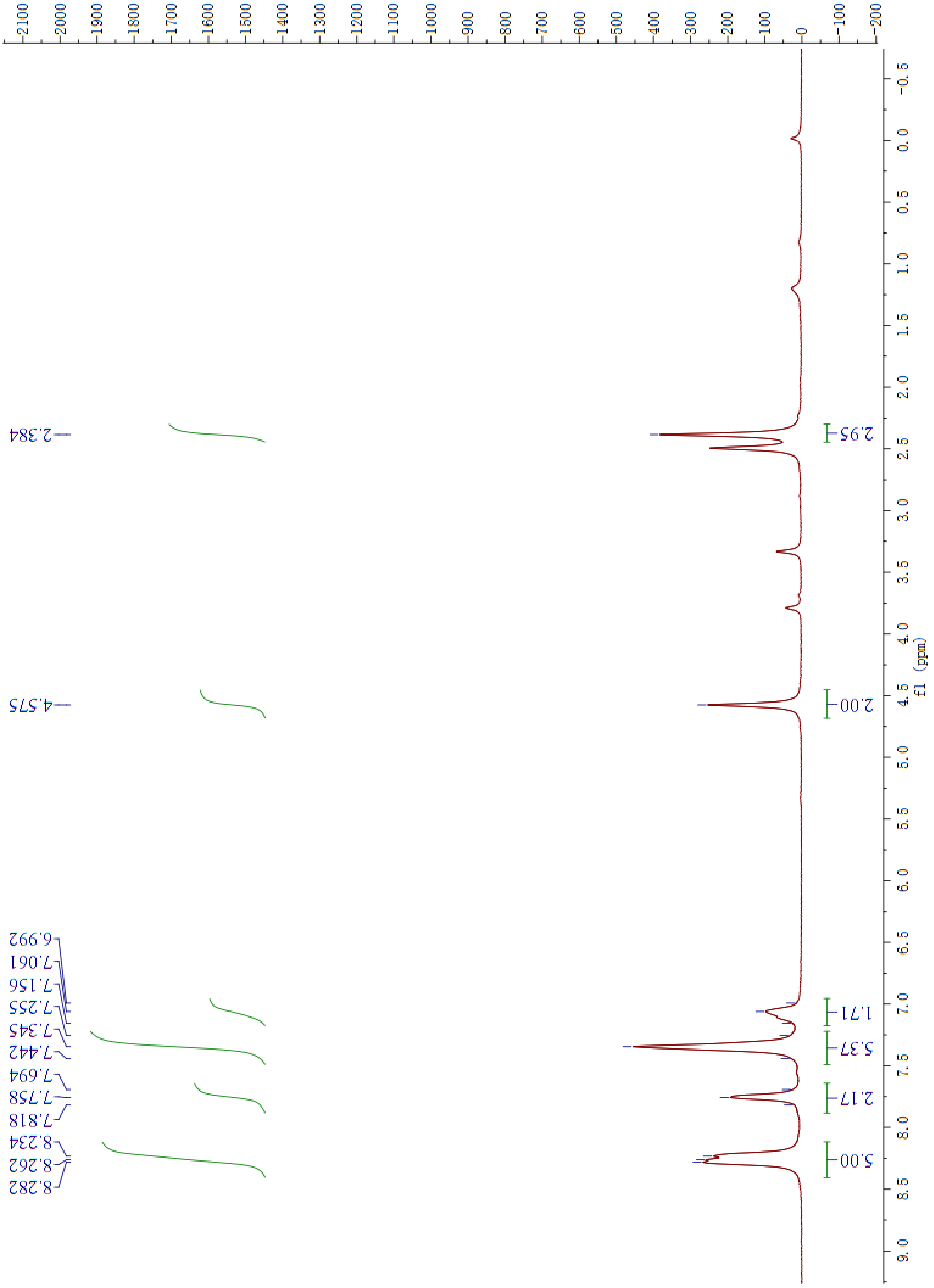

Table S27. ^{1}H NMR of compound 6n

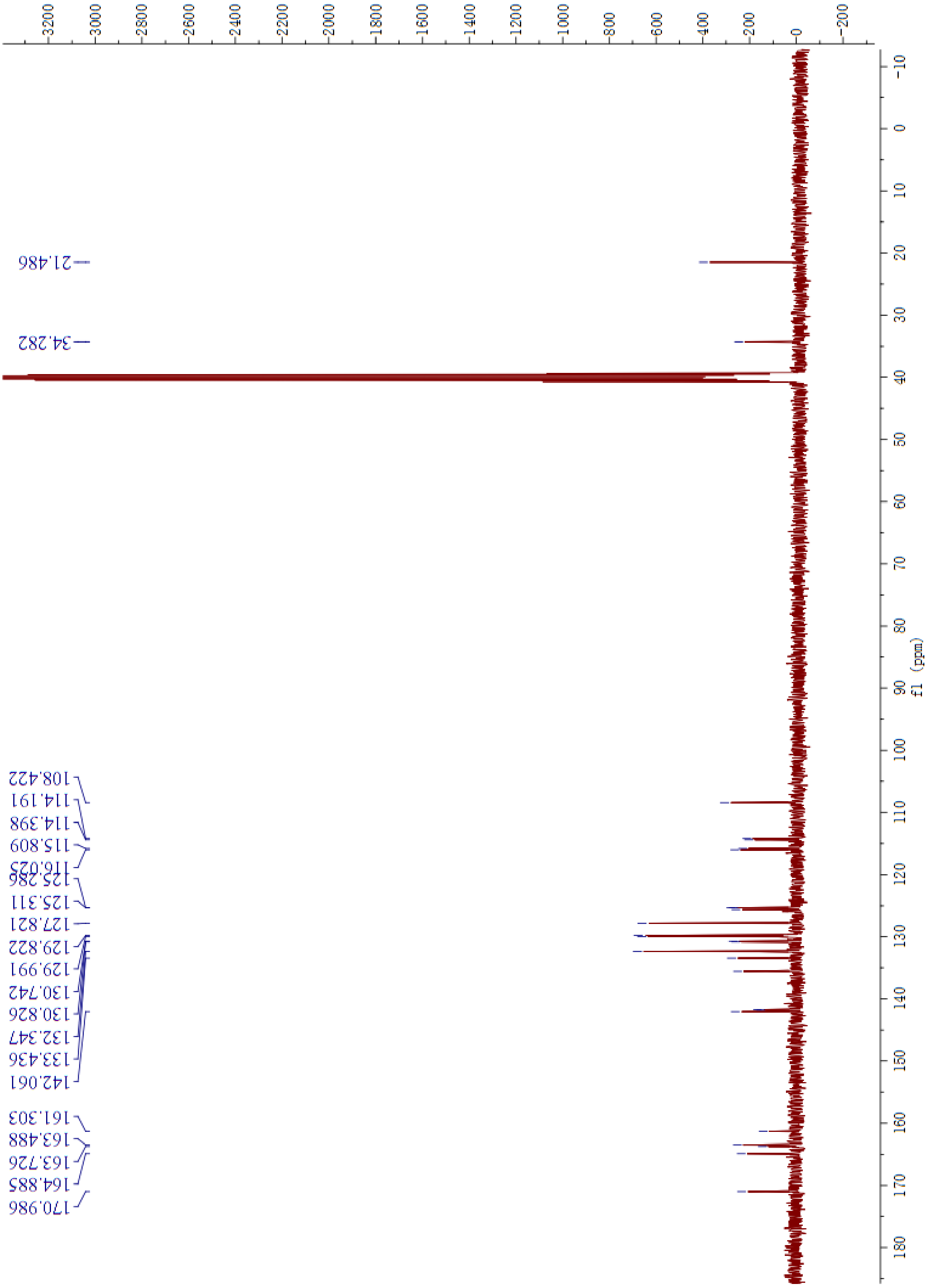

Table S28. ^{13}C NMR of compound 6n

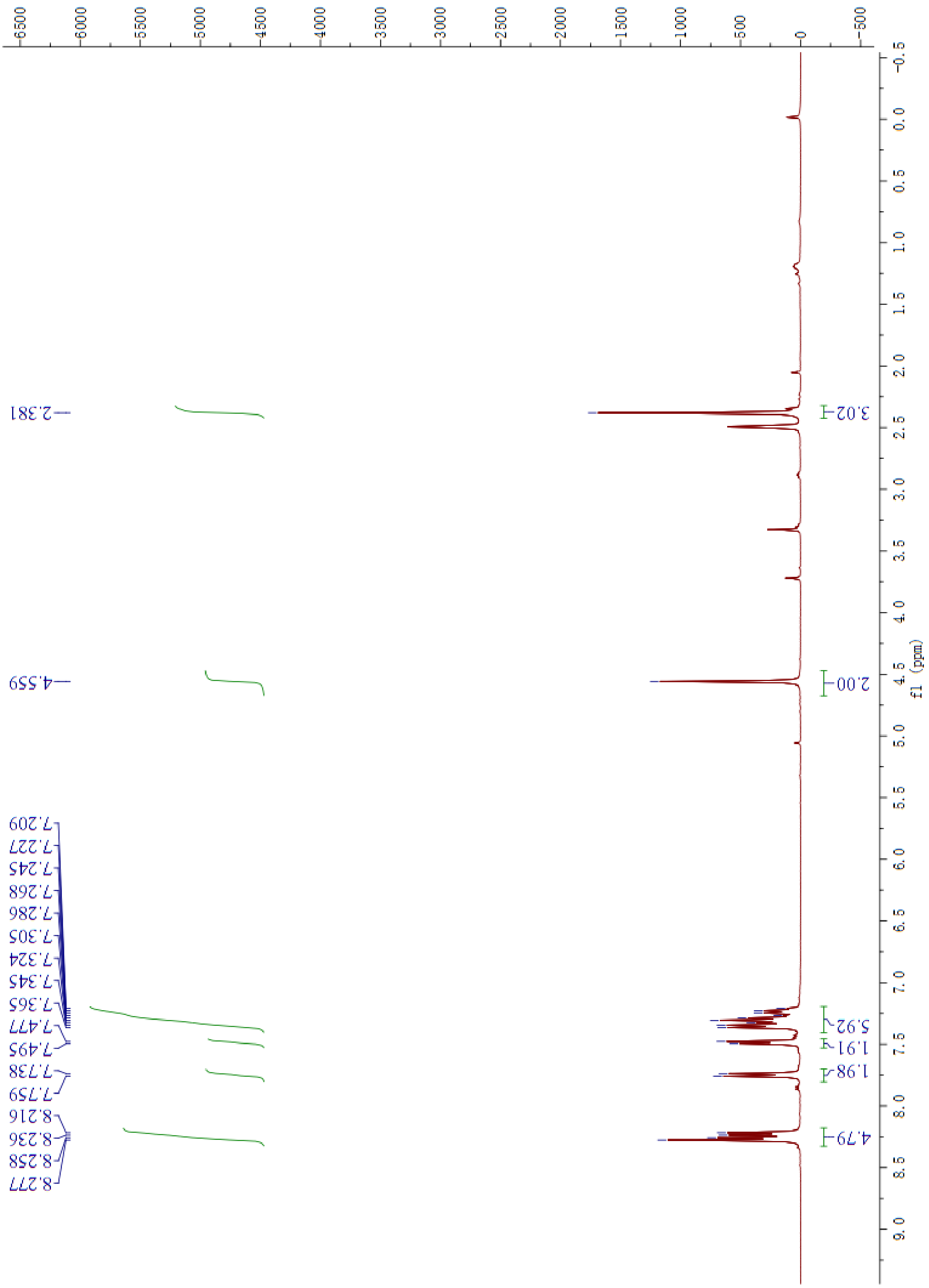

Table S29. ^{1}H NMR of compound 6o

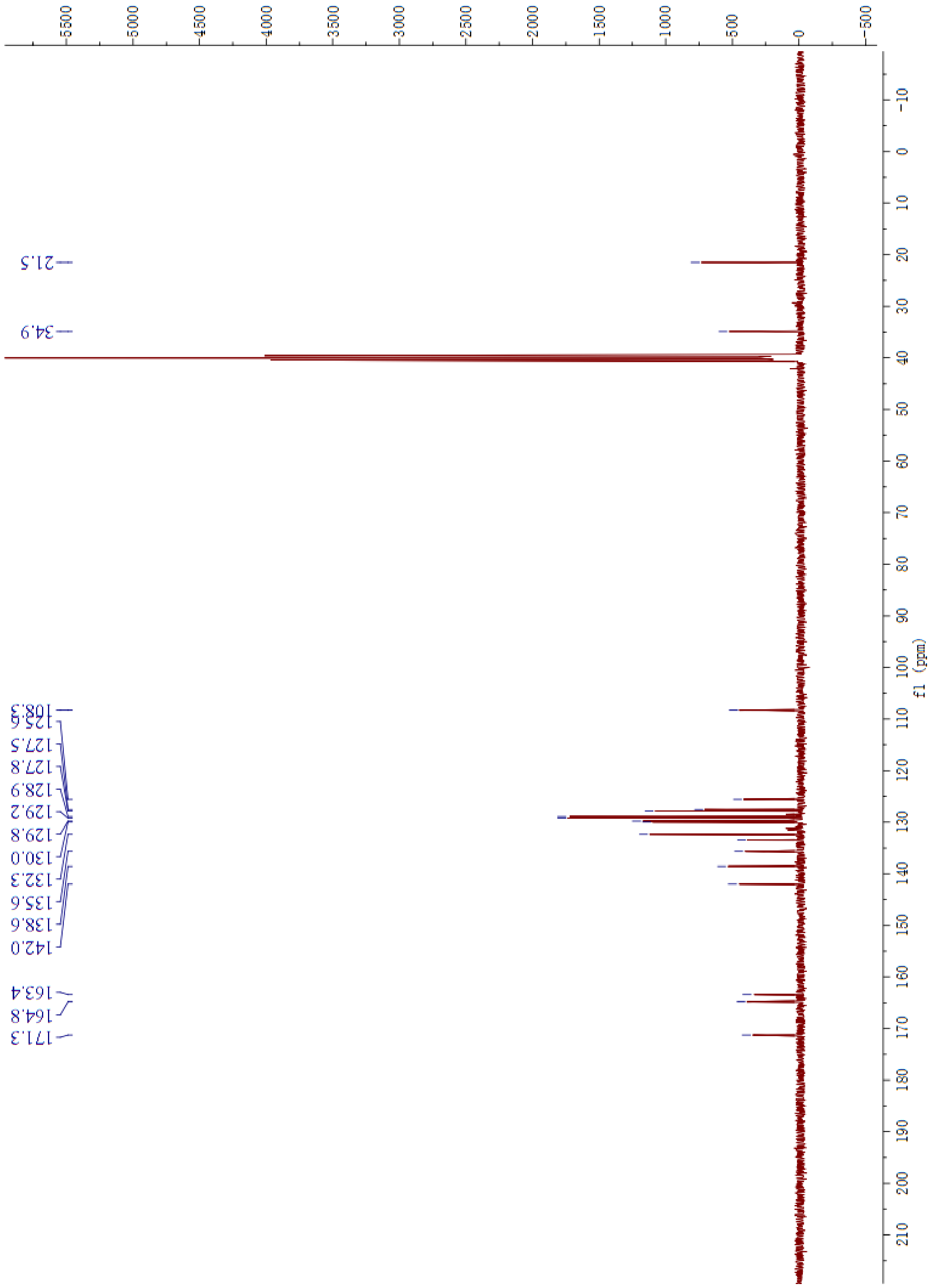

Table S30. ^{13}C NMR of compound 6o

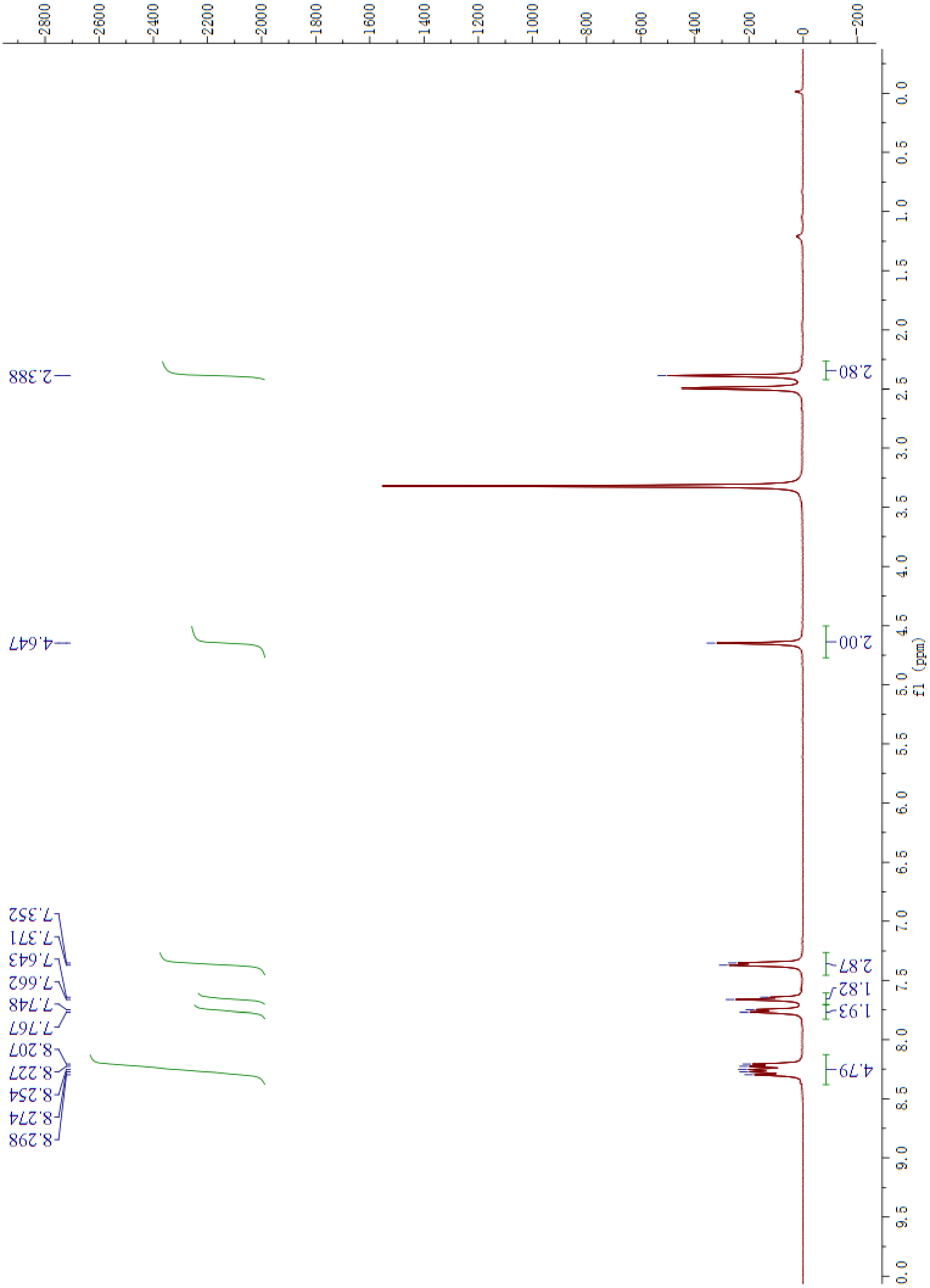

Table S31. ^{1}H NMR of compound 6p

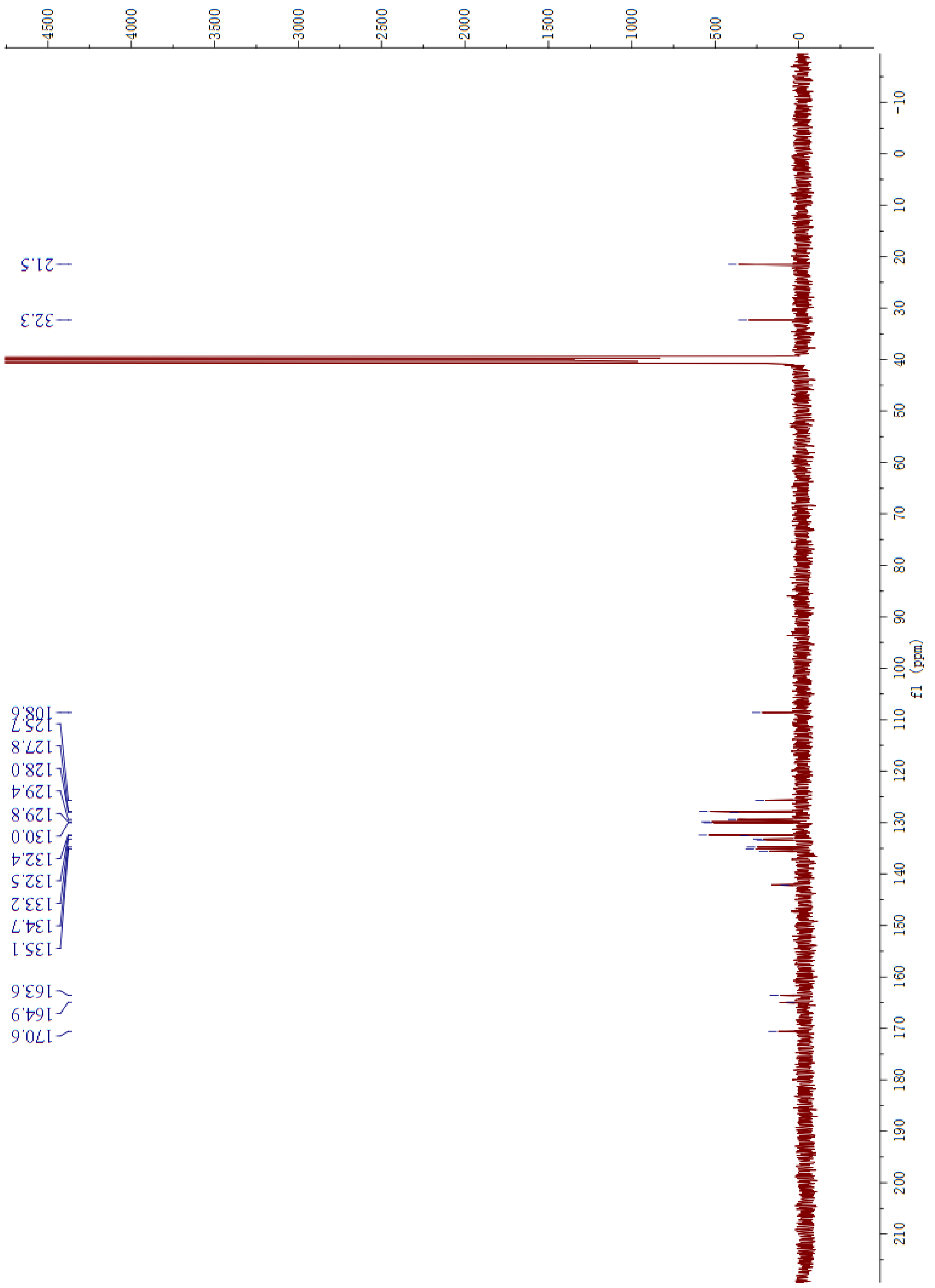

Table S32. ^{13}C NMR of compound 6p

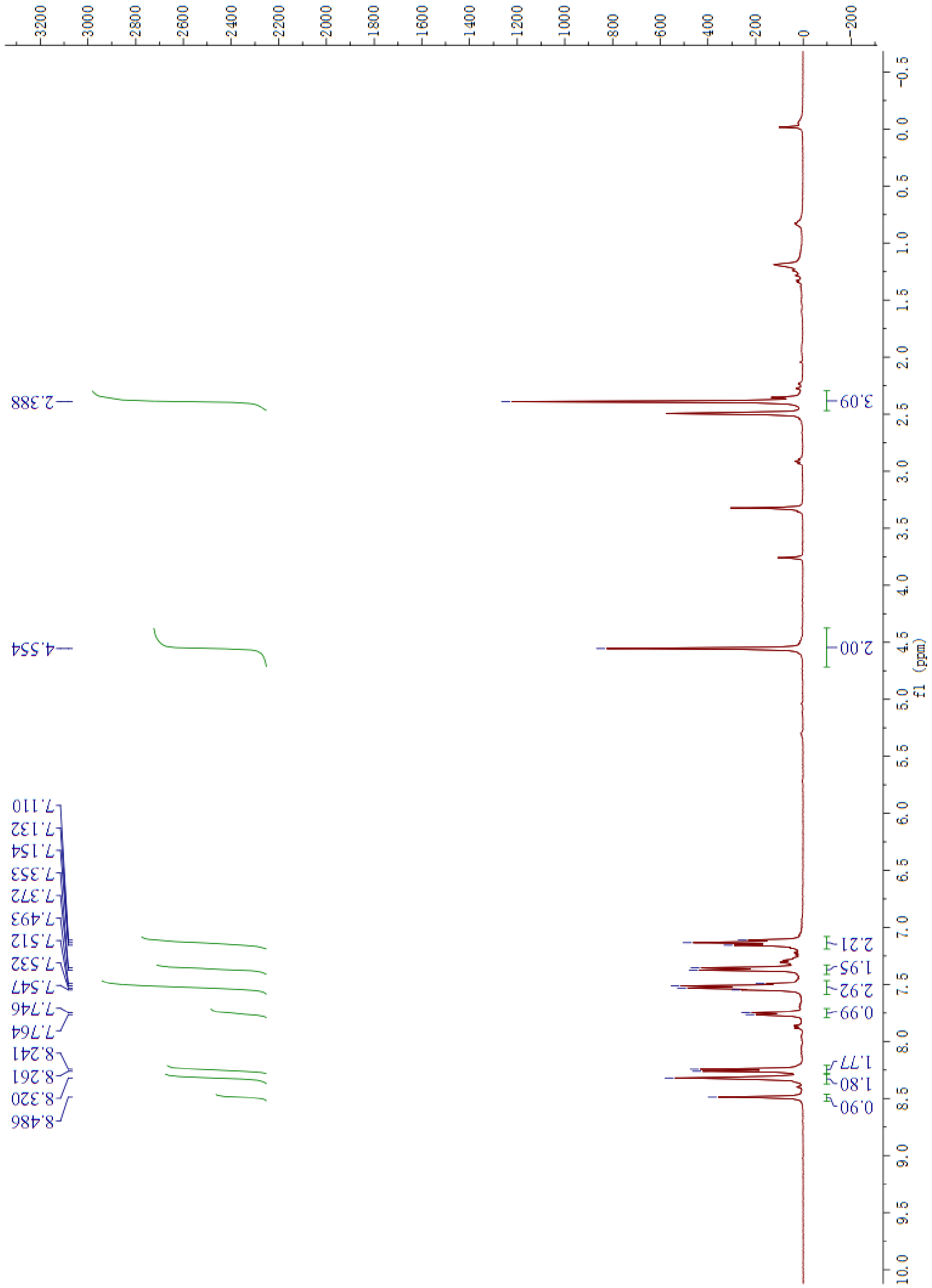

Table S33. ¹H NMR of compound 6q

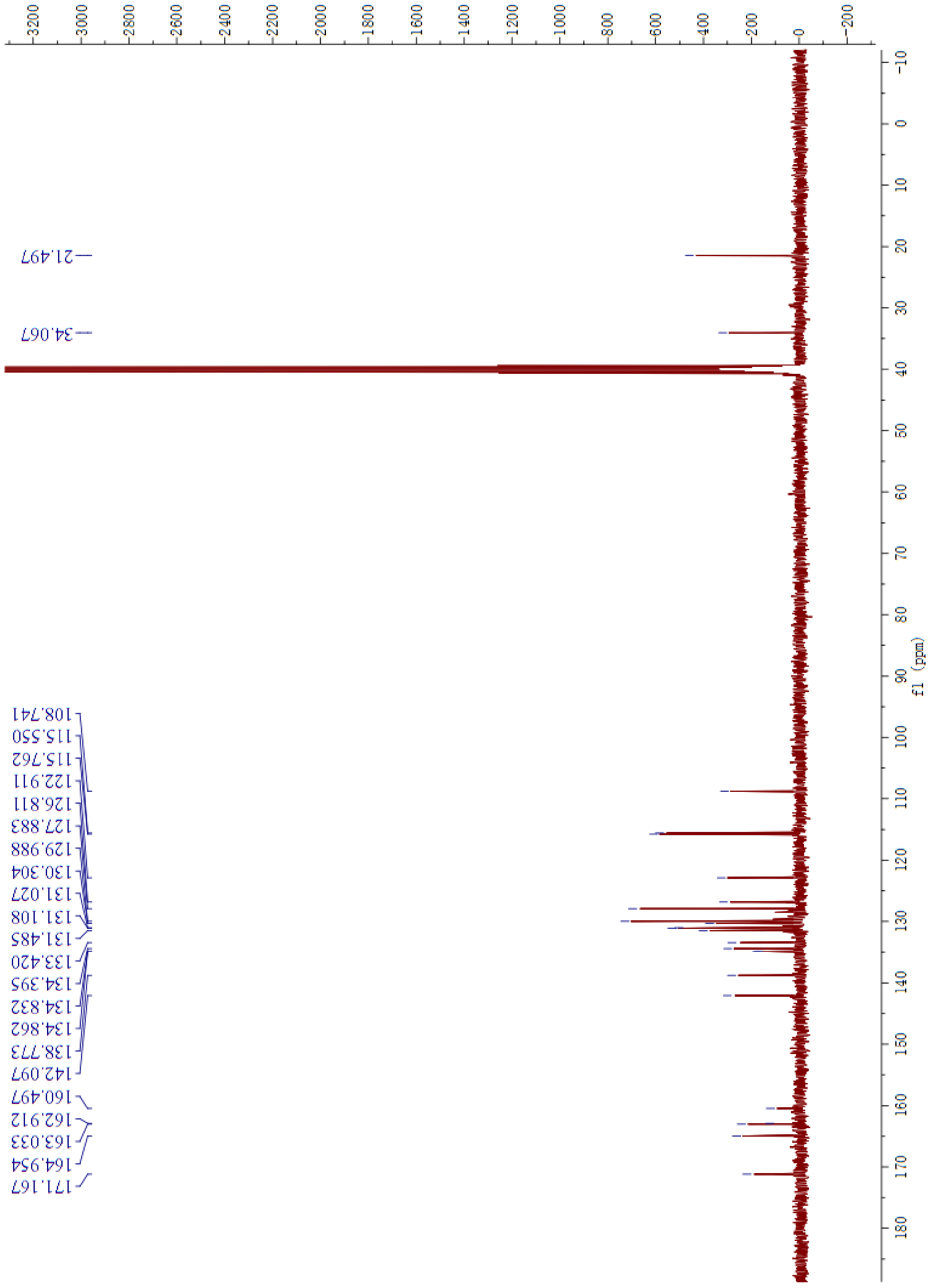

Table S34. ^{13}C NMR of compound 6q

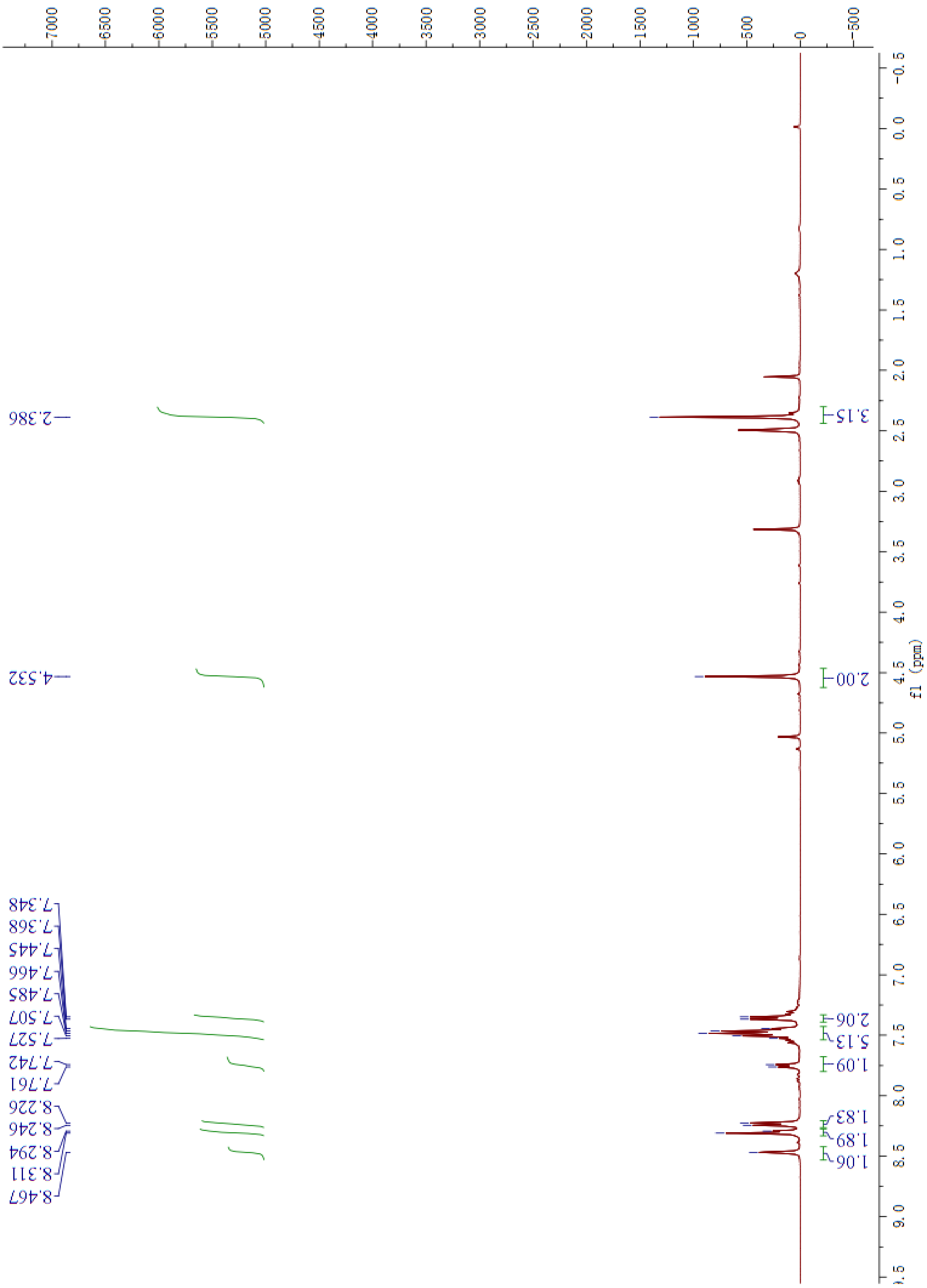

Table S35. ^{1}H NMR of compound 6r

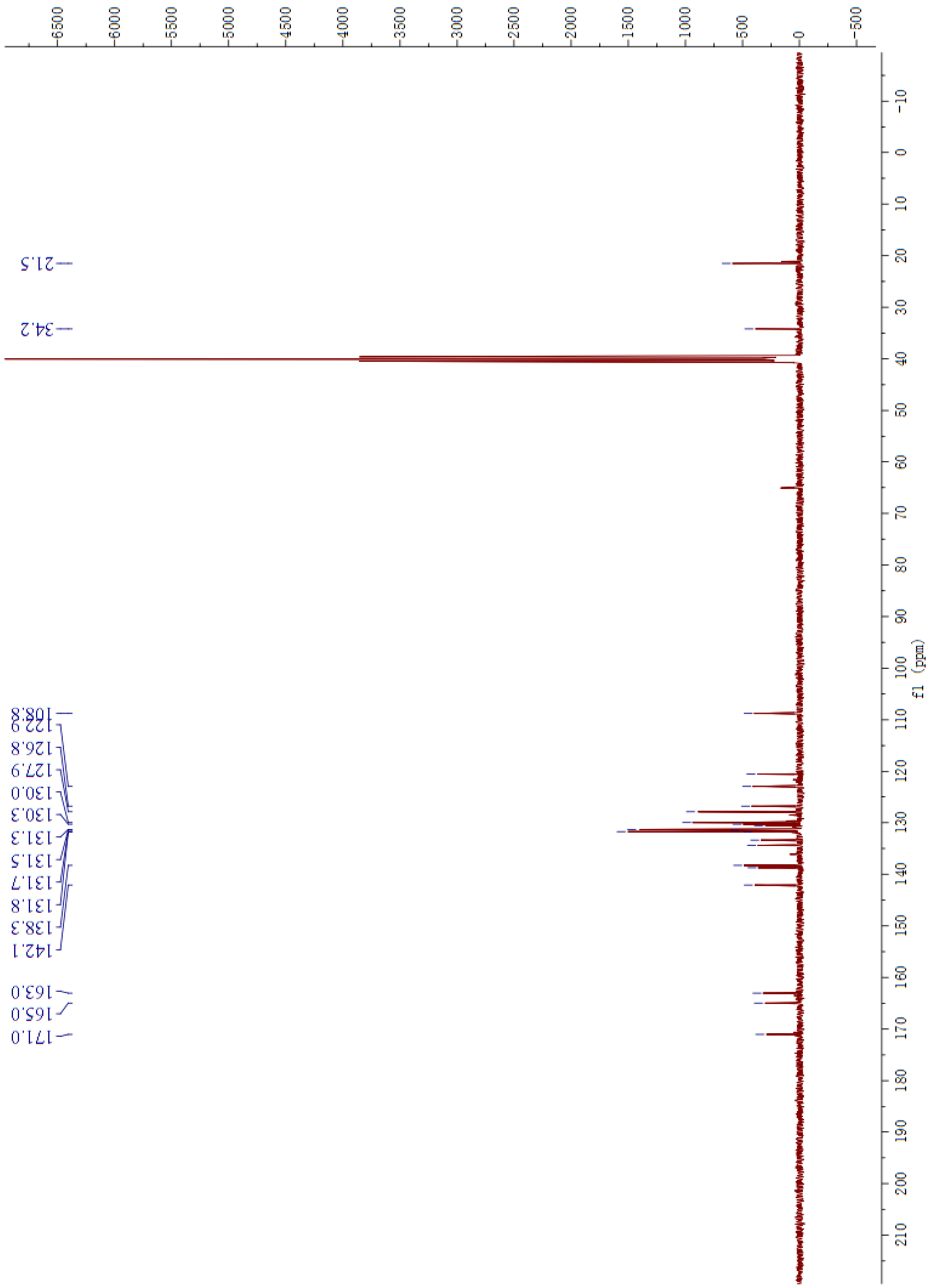

Table S36. ^{13}C NMR of compound 6r

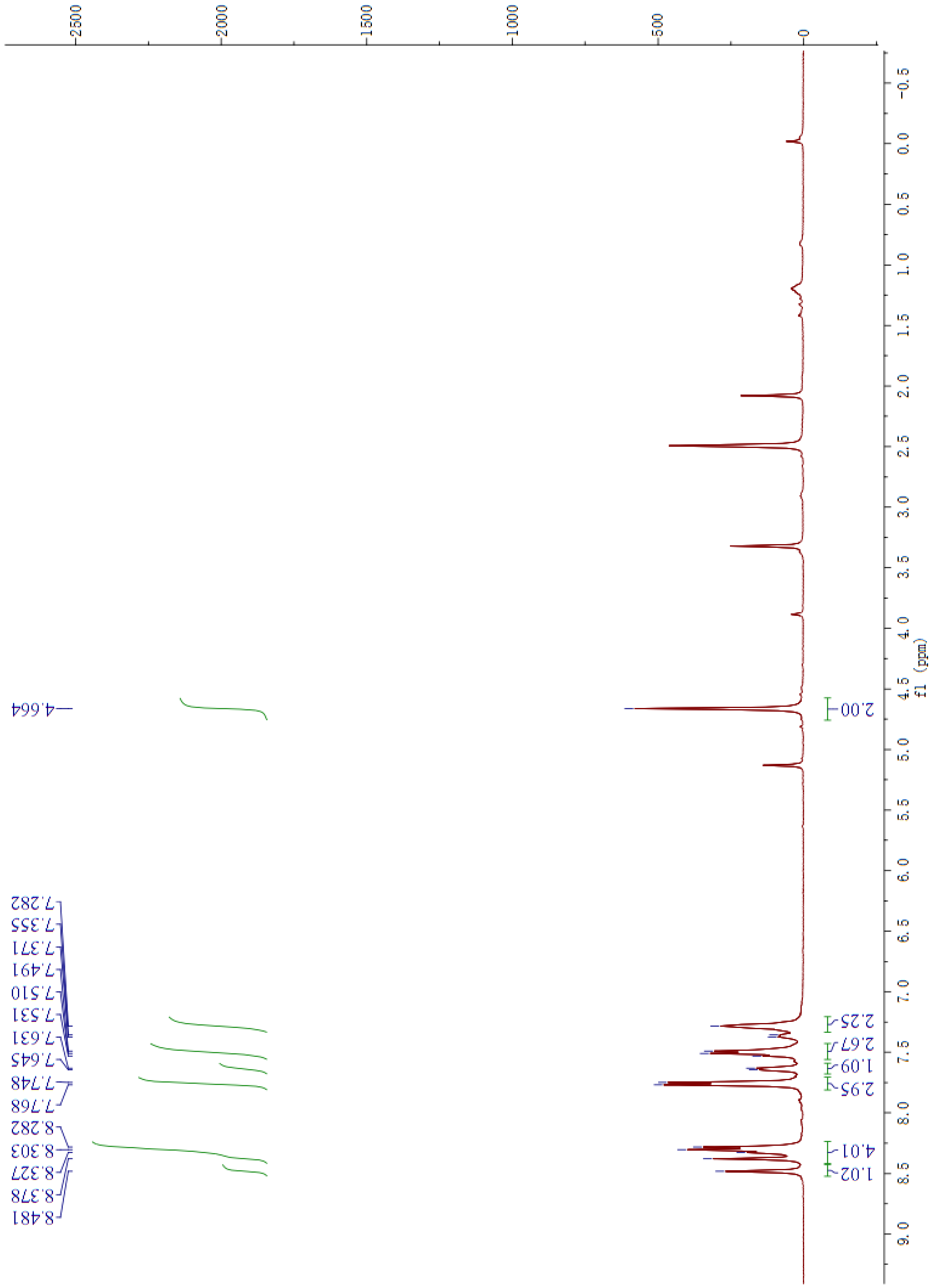

Table S37. ^{1}H NMR of compound 6s

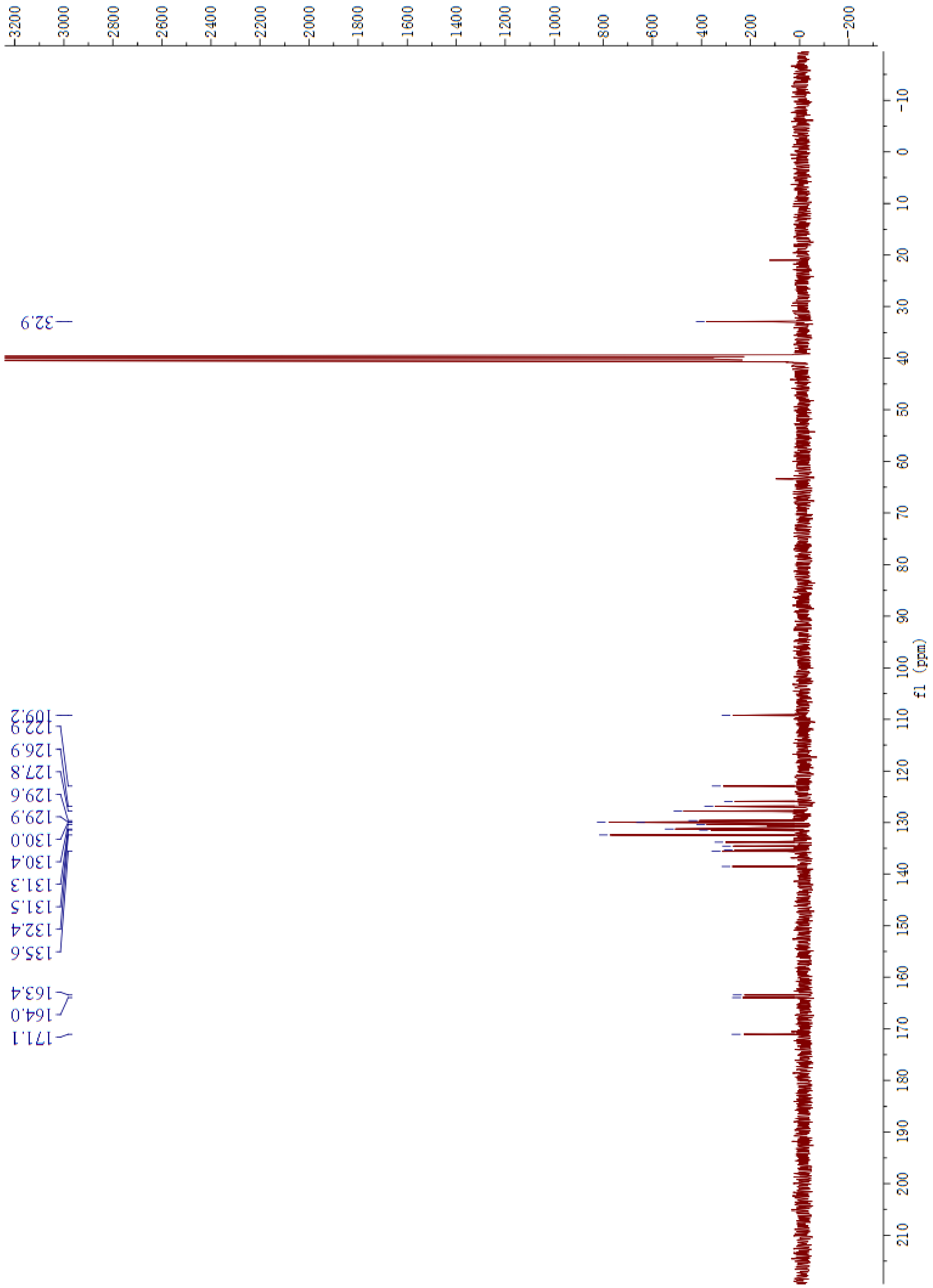

Table S38. ^{13}C NMR of compound 6s

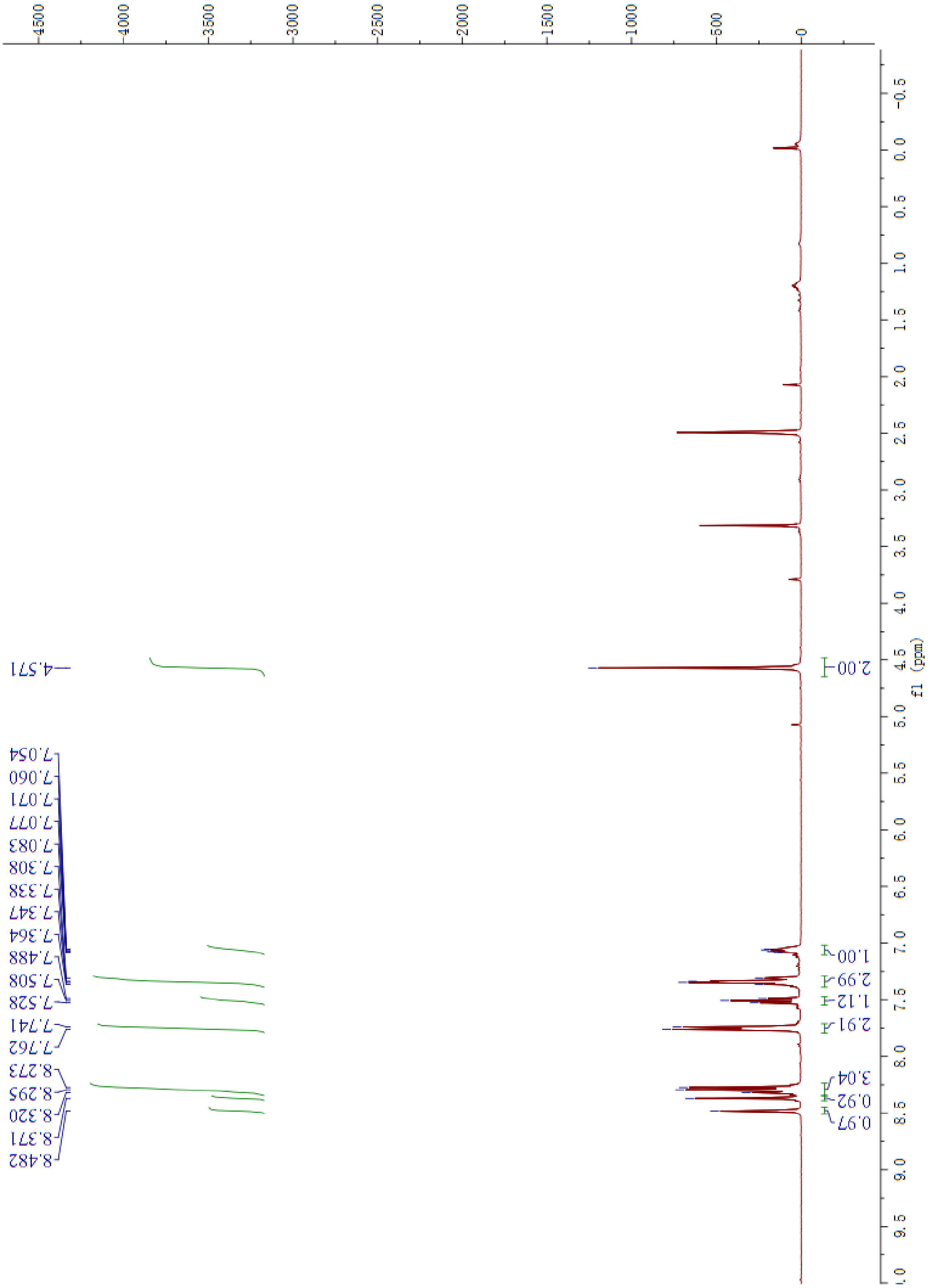

Table S39. ^{1}H NMR of compound 6t

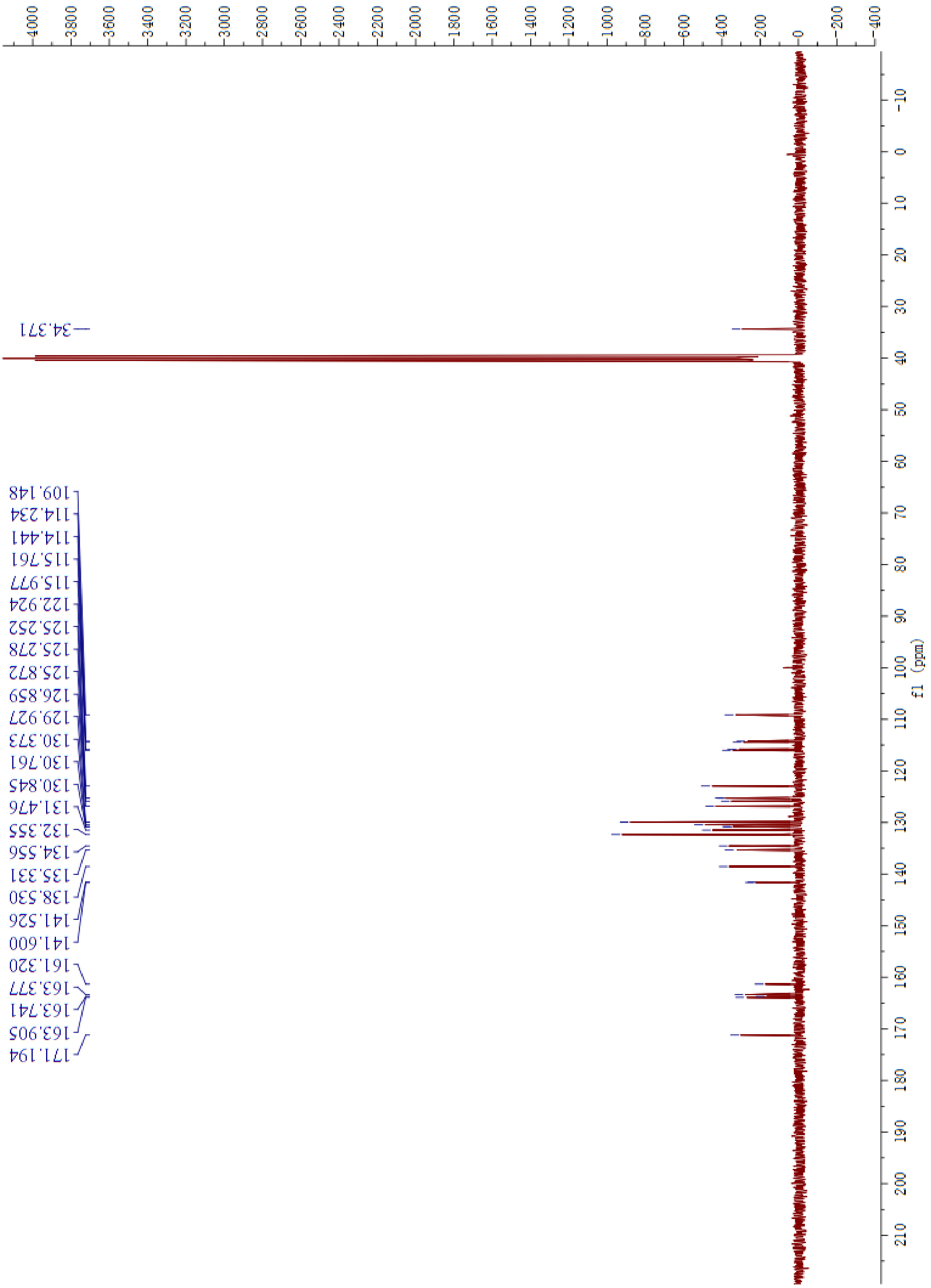

Table S40. ^{13}C NMR of compound 6t

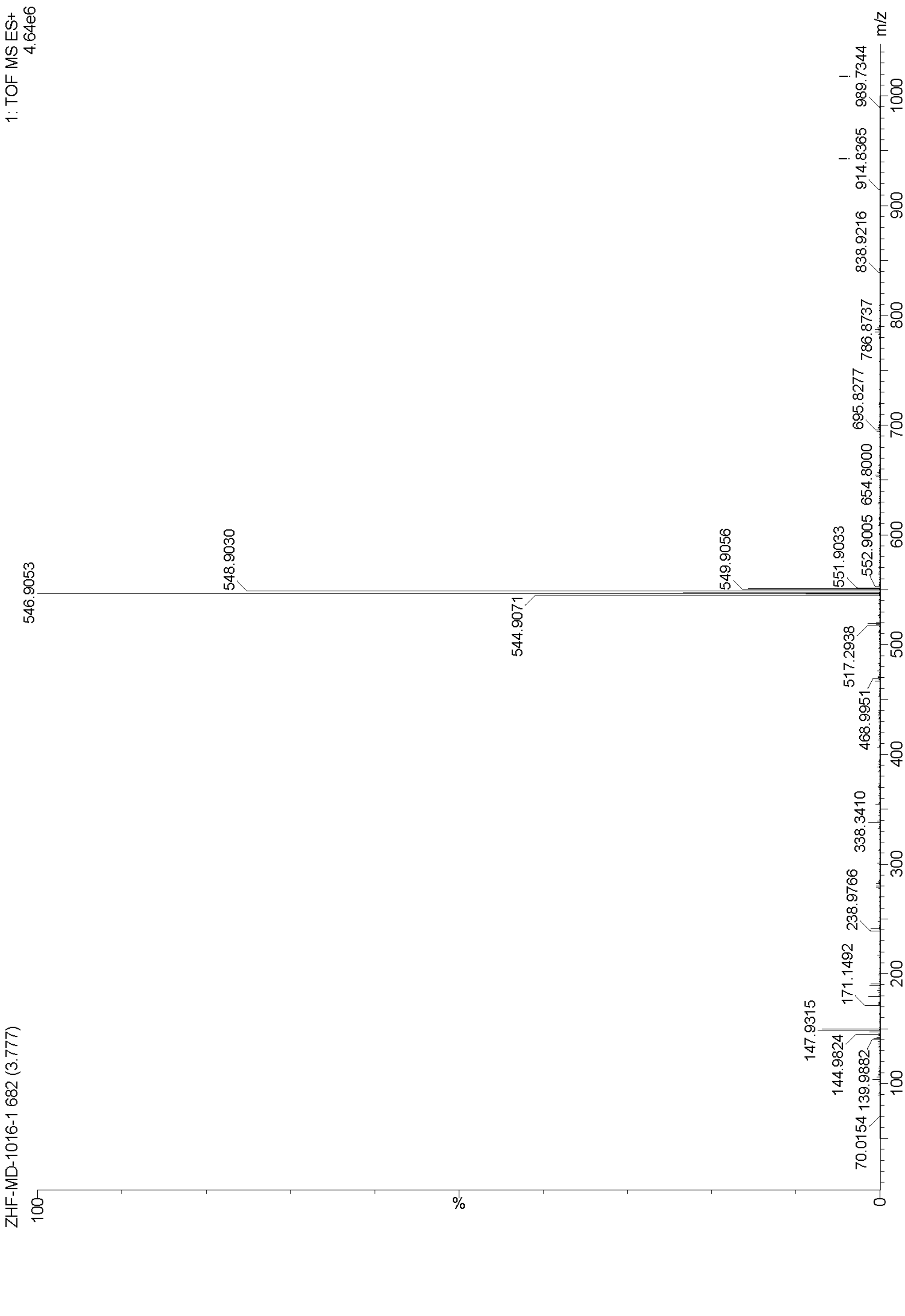

Table S41. HRMS of compound 6a

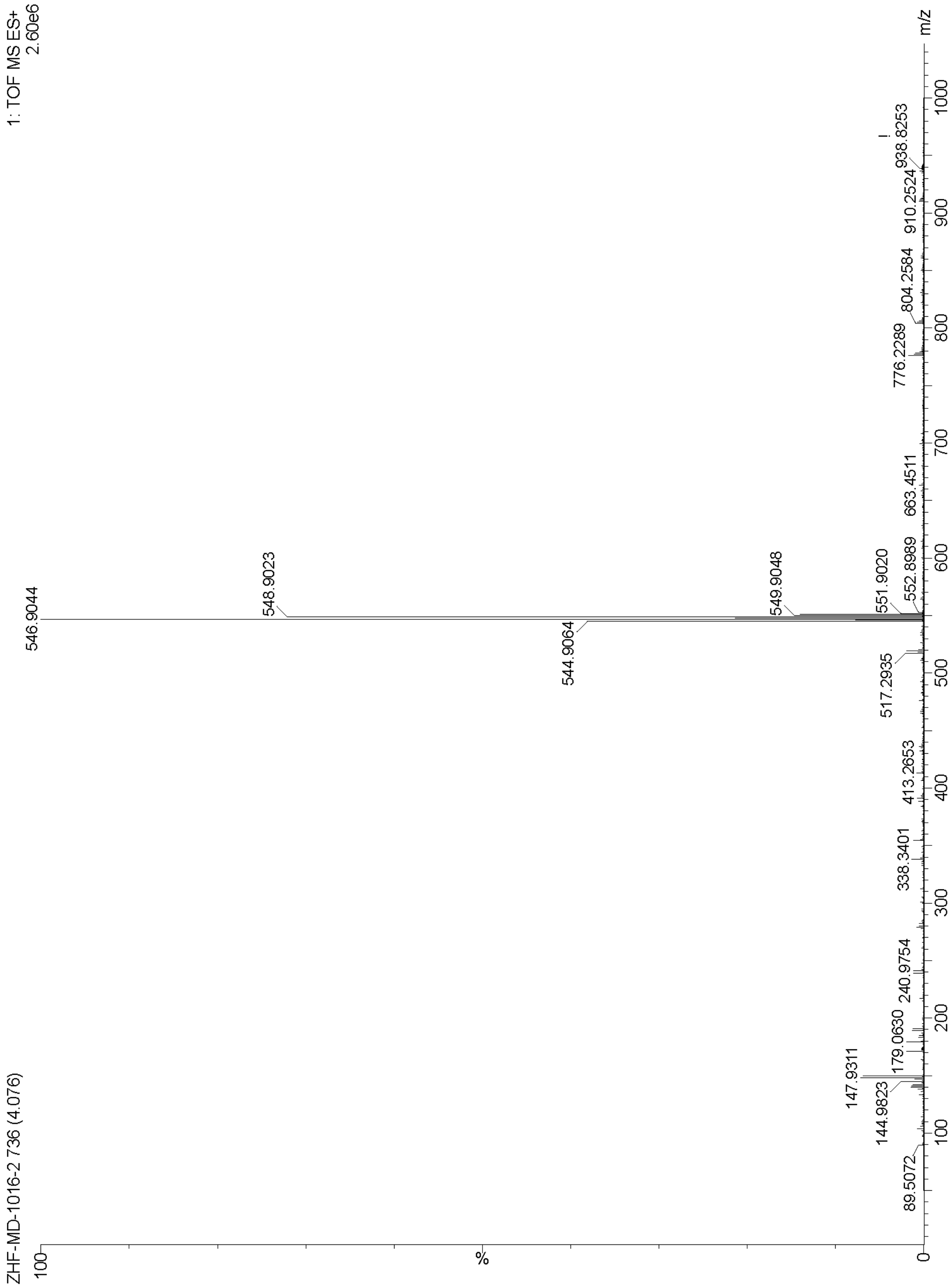

Table S42. HRMS of compound 6b

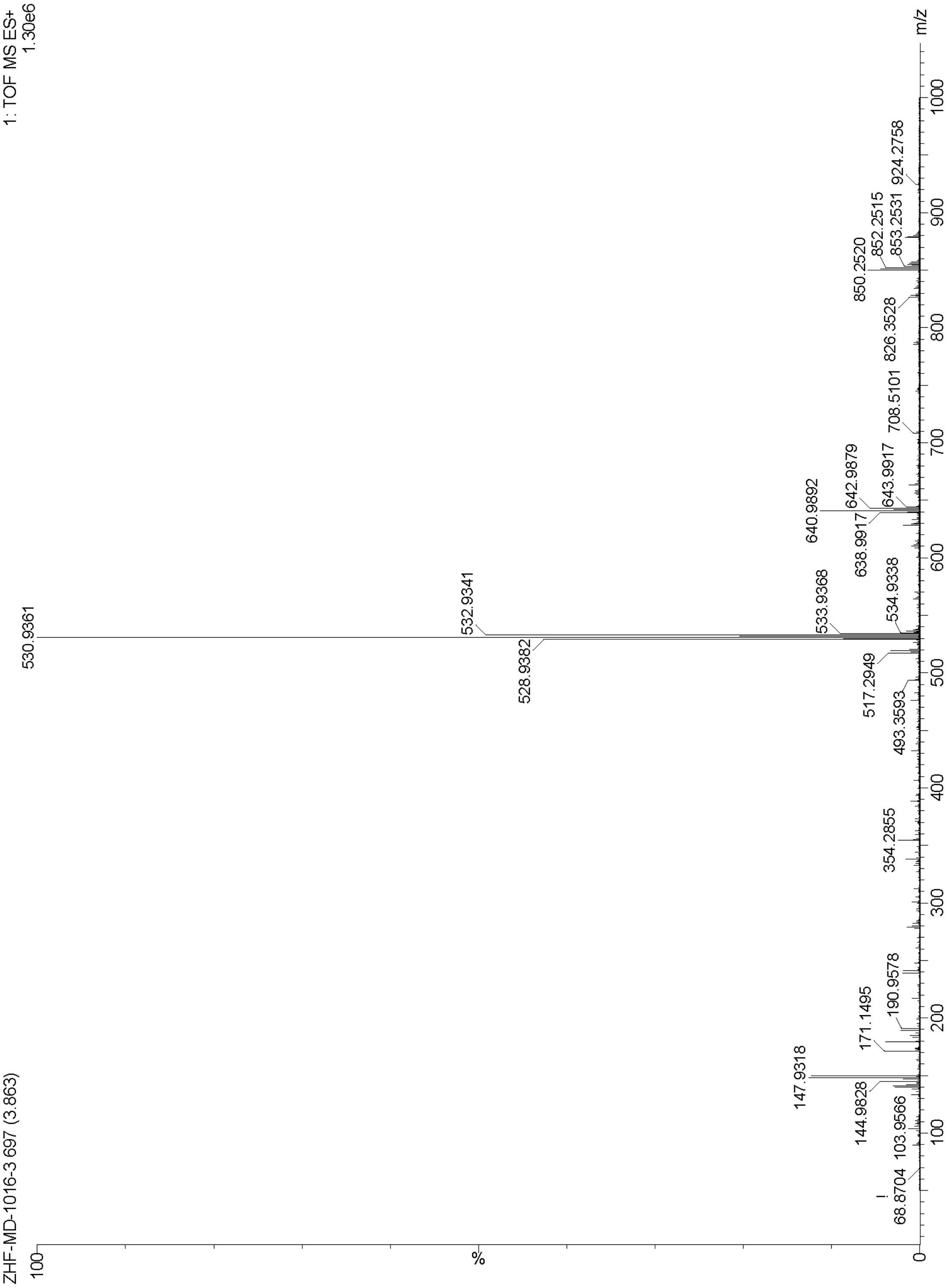

Table S43. HRMS of compound 6c

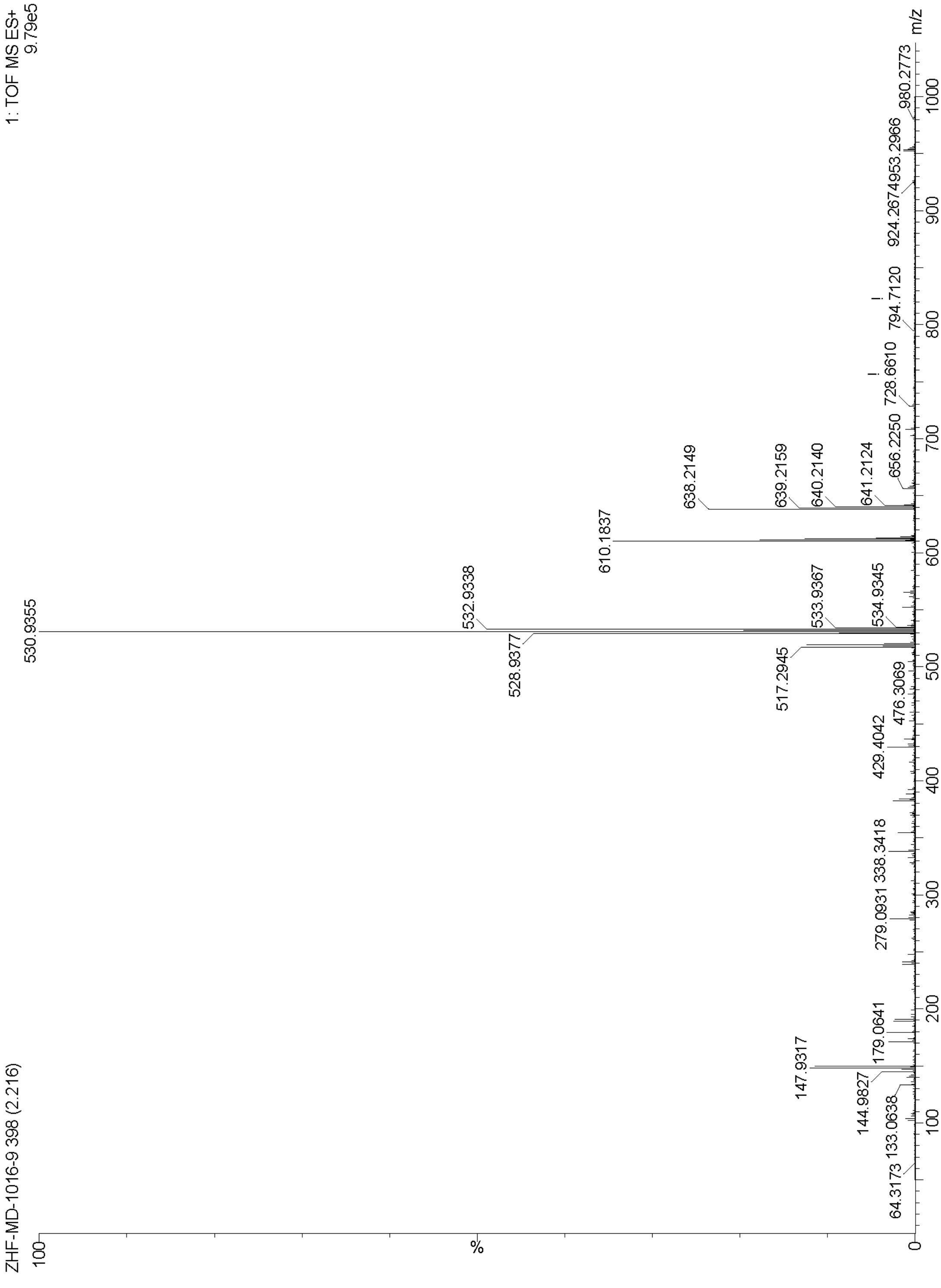

Table S44. HRMS of compound 6d

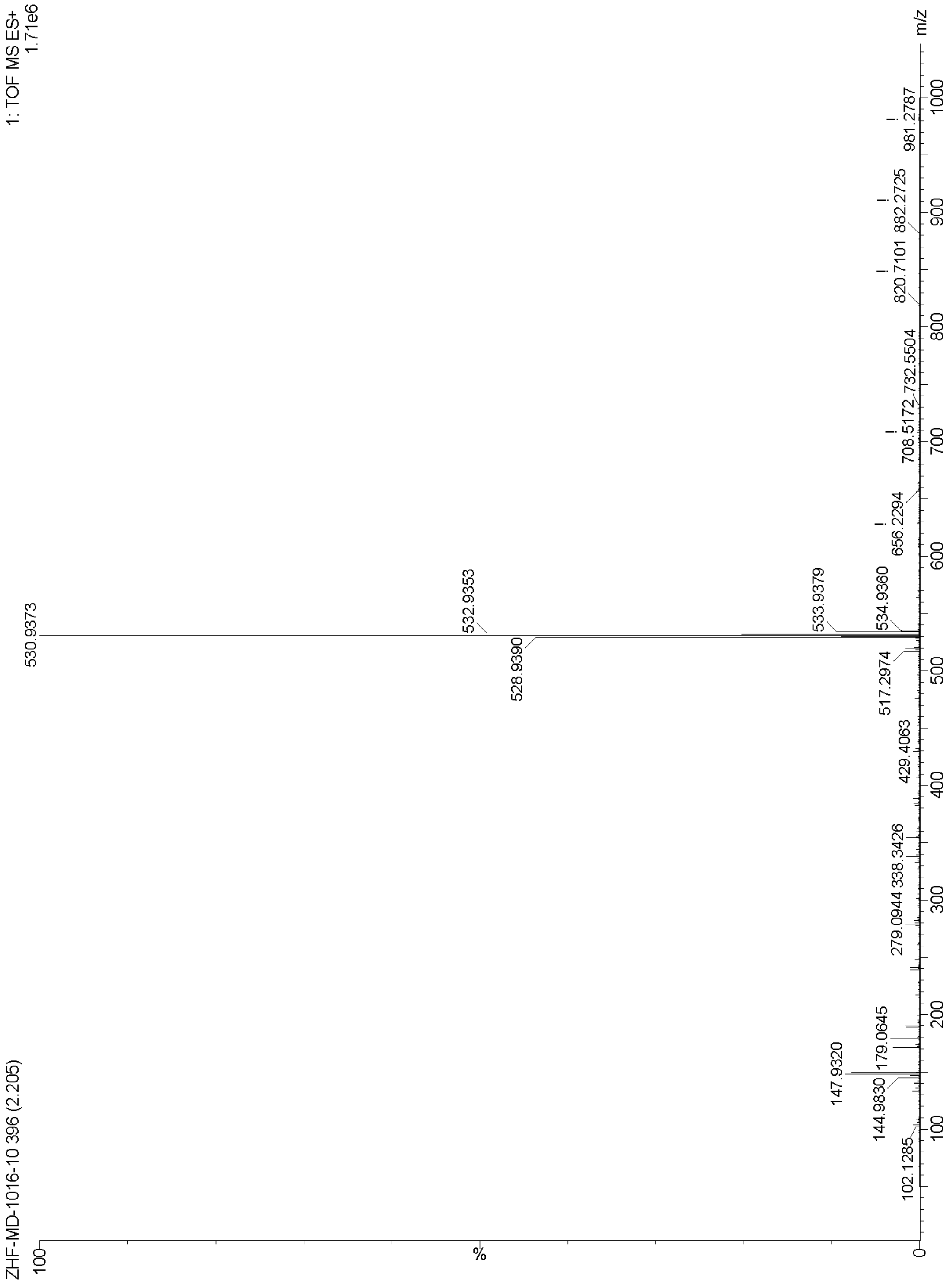

Table S45. HRMS of compound 6e

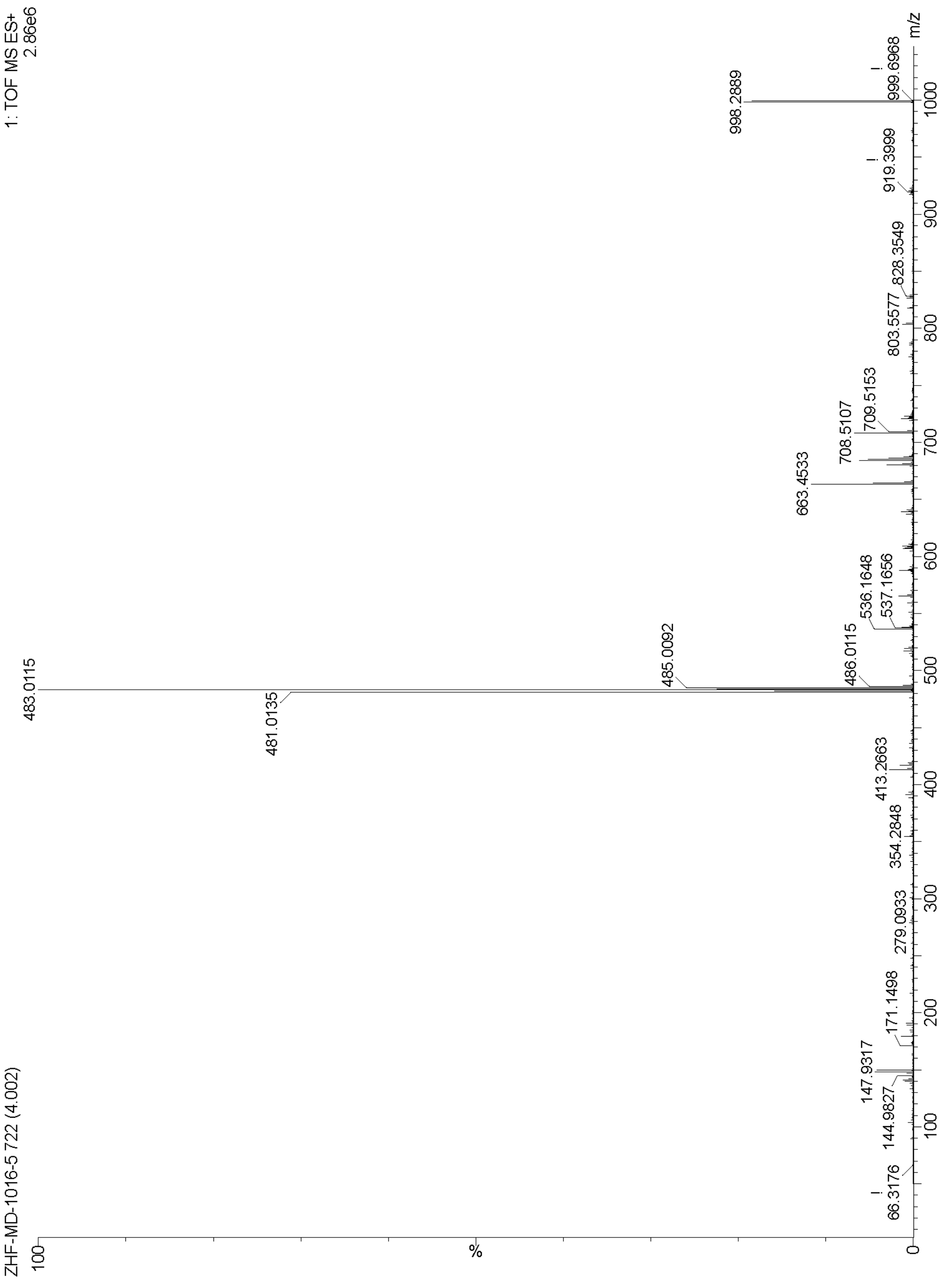

Table S46. HRMS of compound 6f

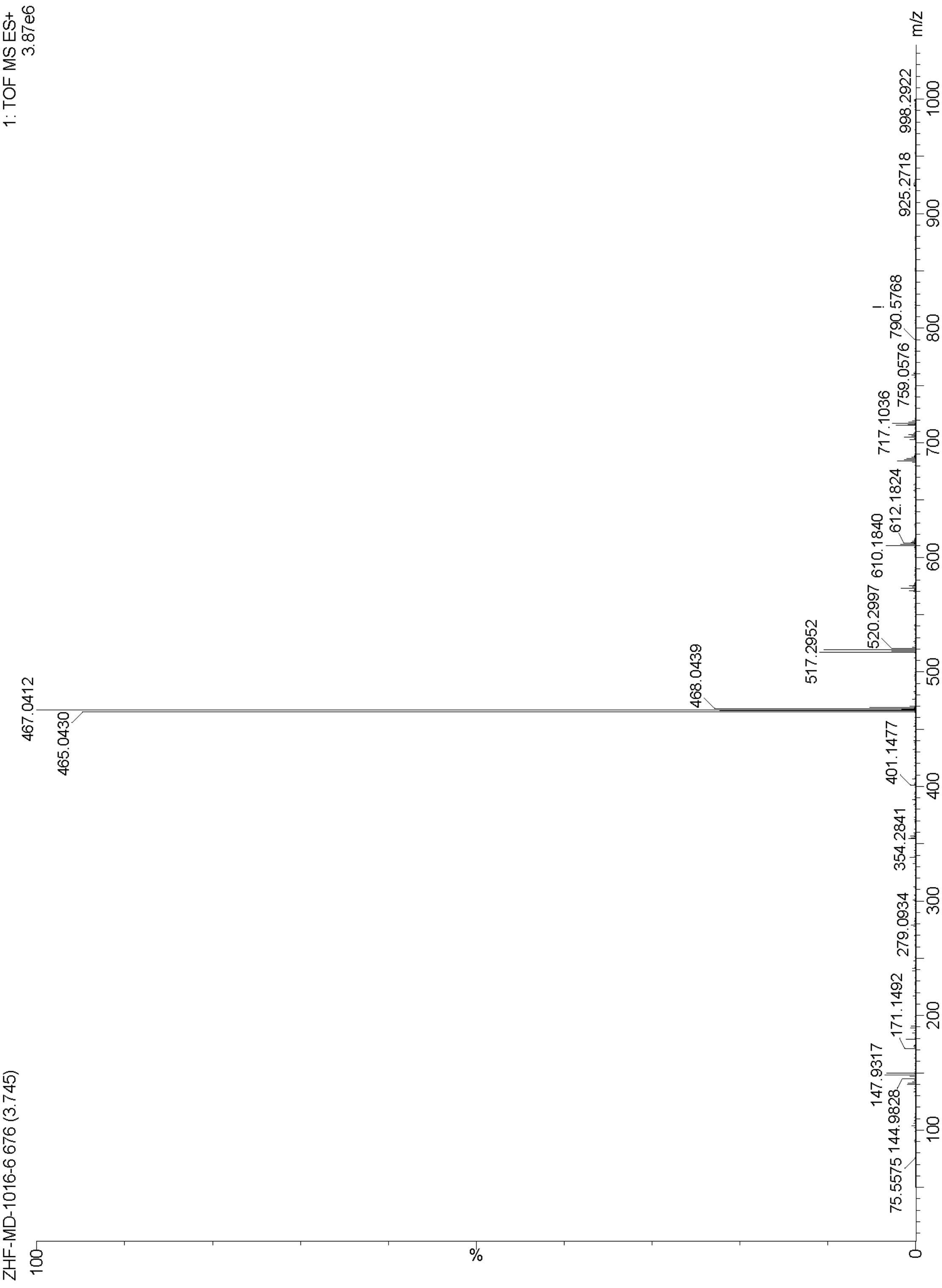

Table S47. HRMS of compound 6g

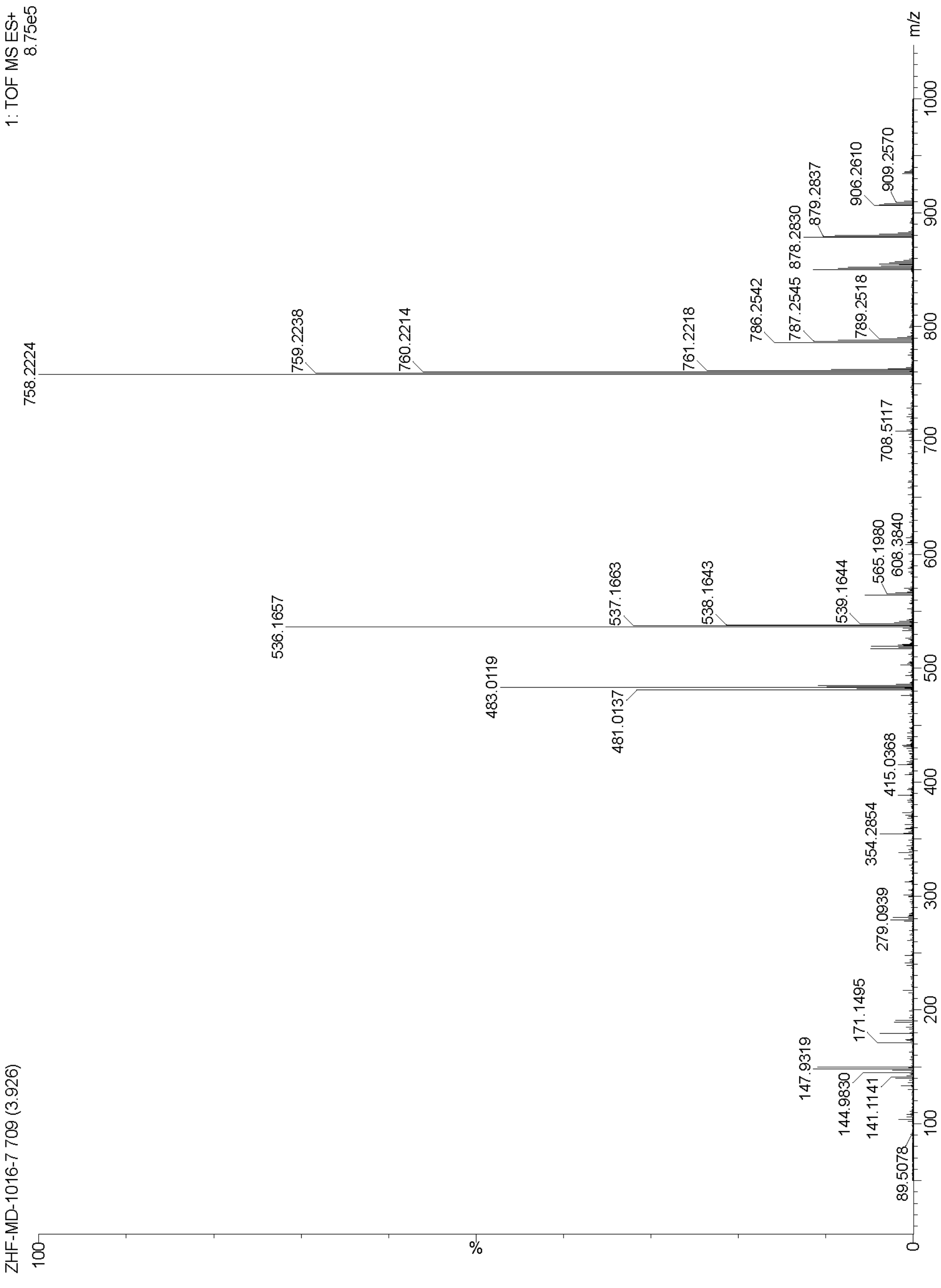

Table S48. HRMS of compound 6h

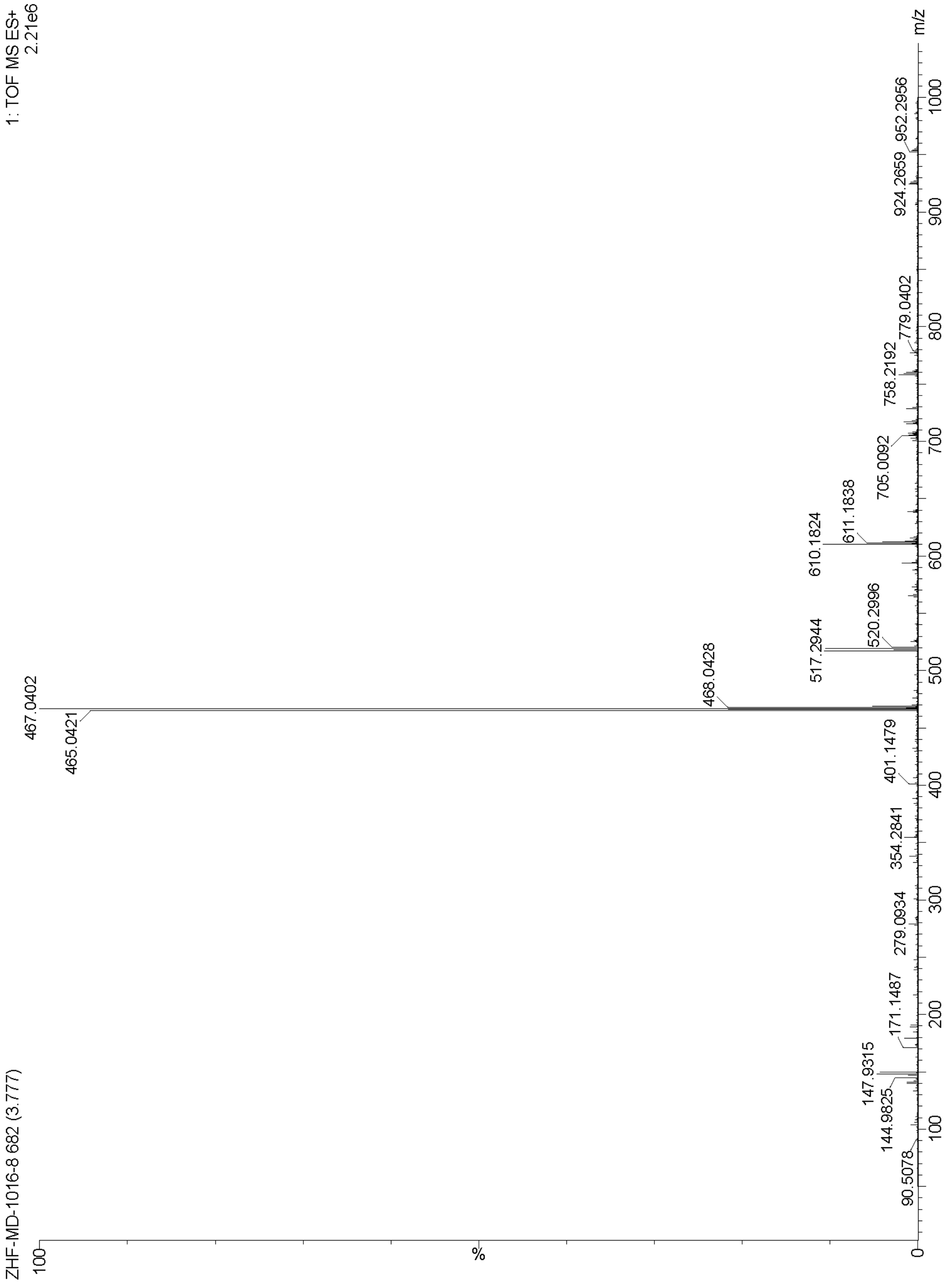

Table S49. HRMS of compound 6i

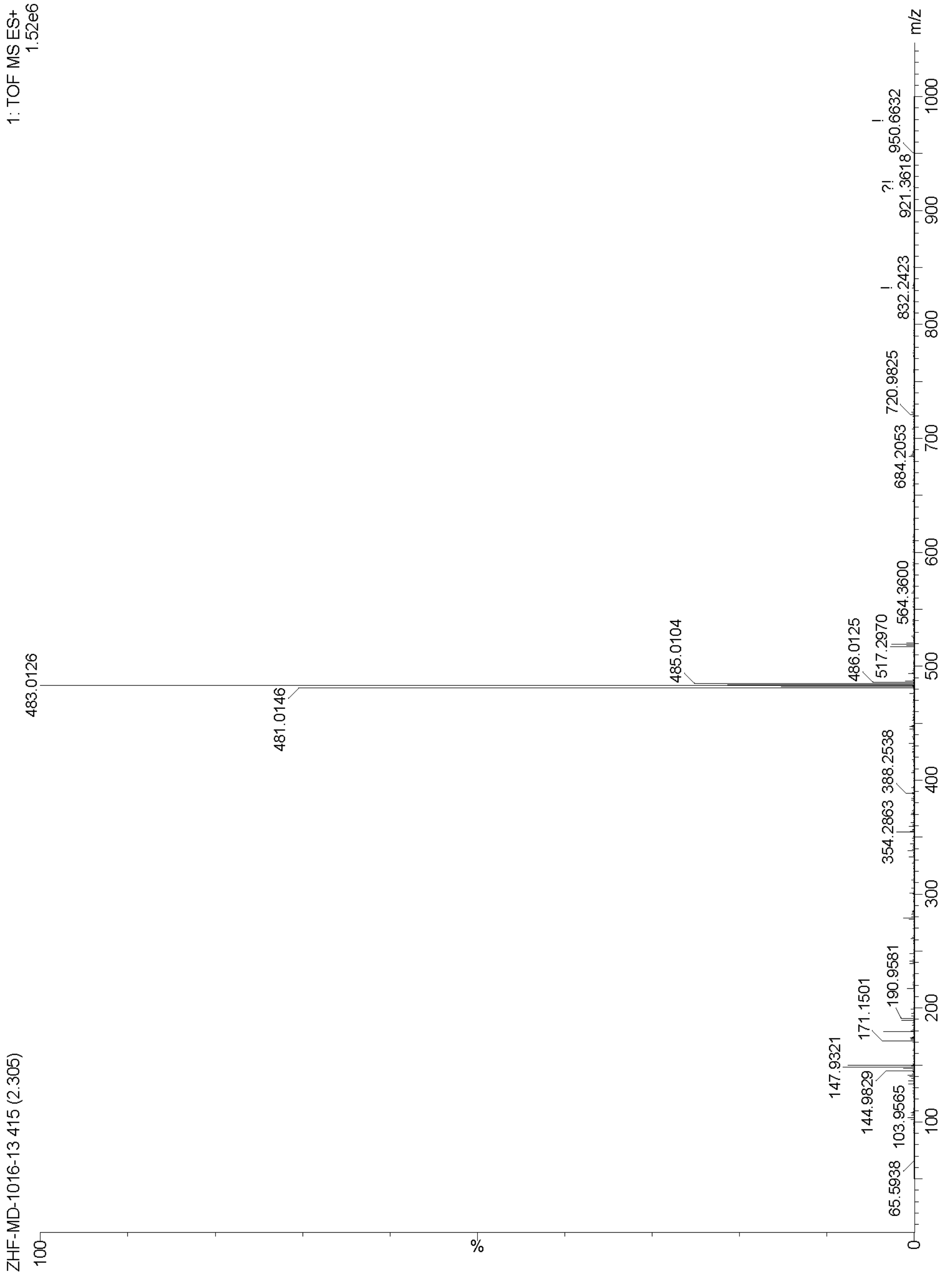

Table S50. HRMS of compound 6j

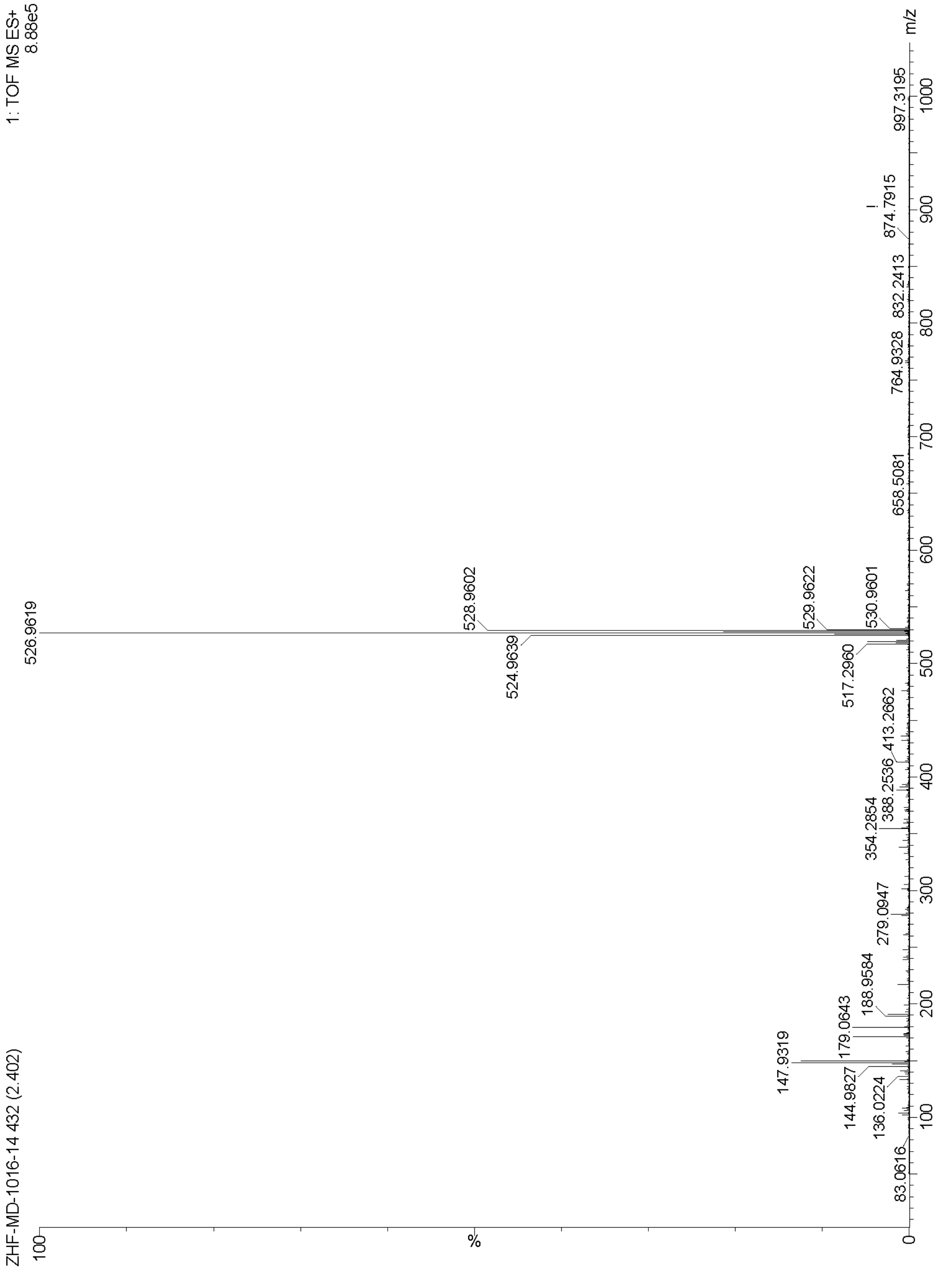

Table S51. HRMS of compound 6k

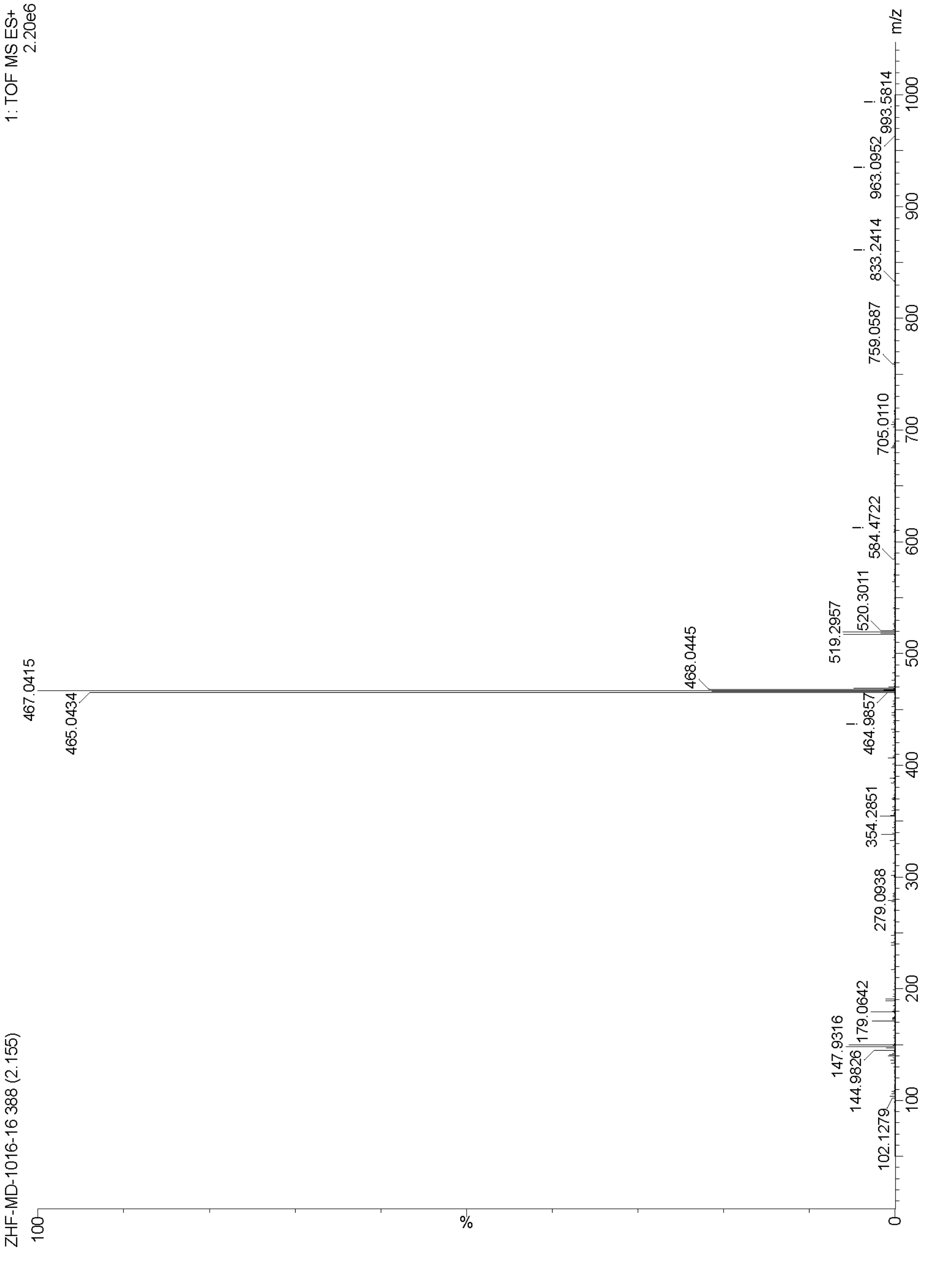

Table S52. HRMS of compound 6l

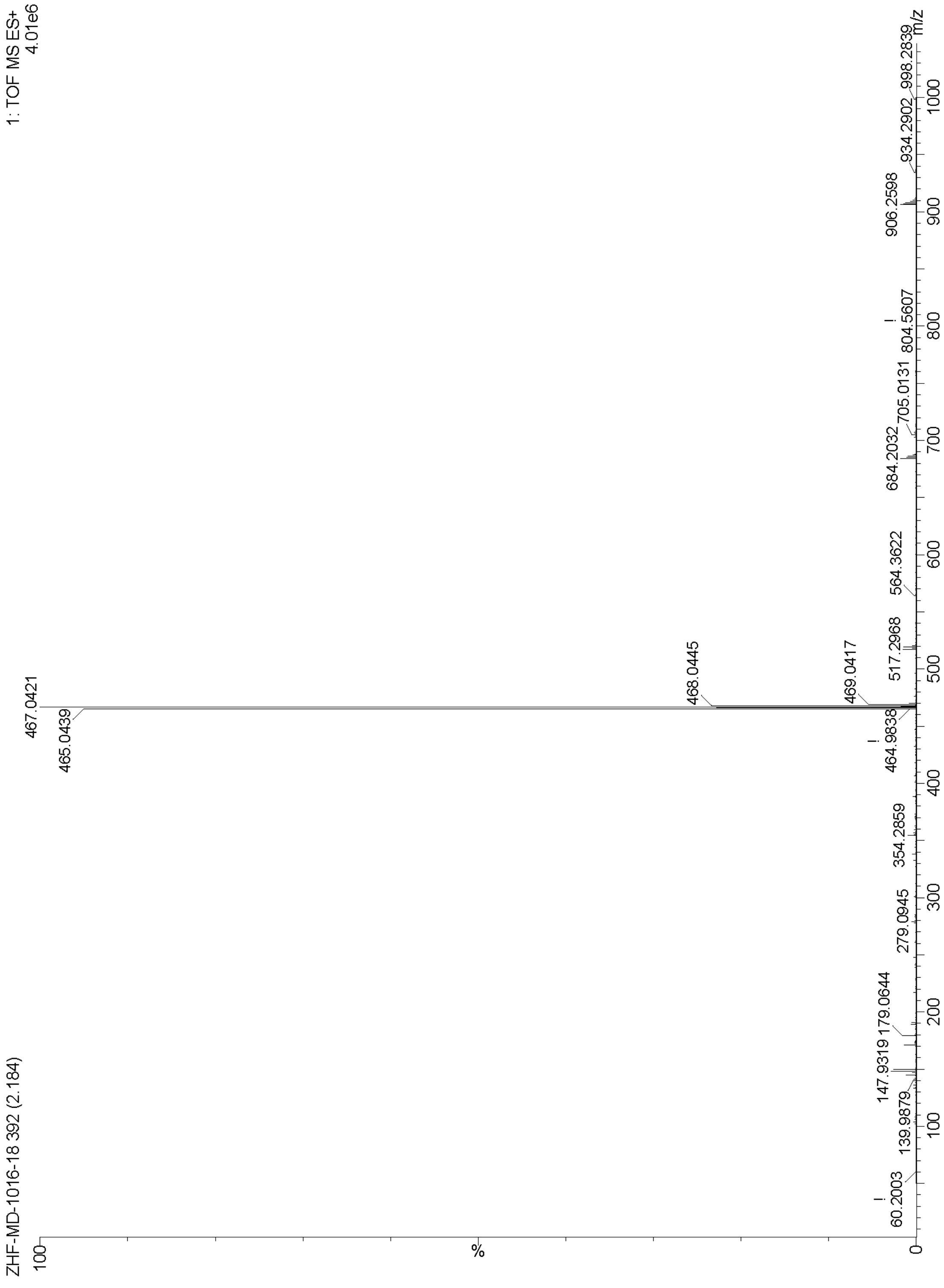

Table S53. HRMS of compound 6m

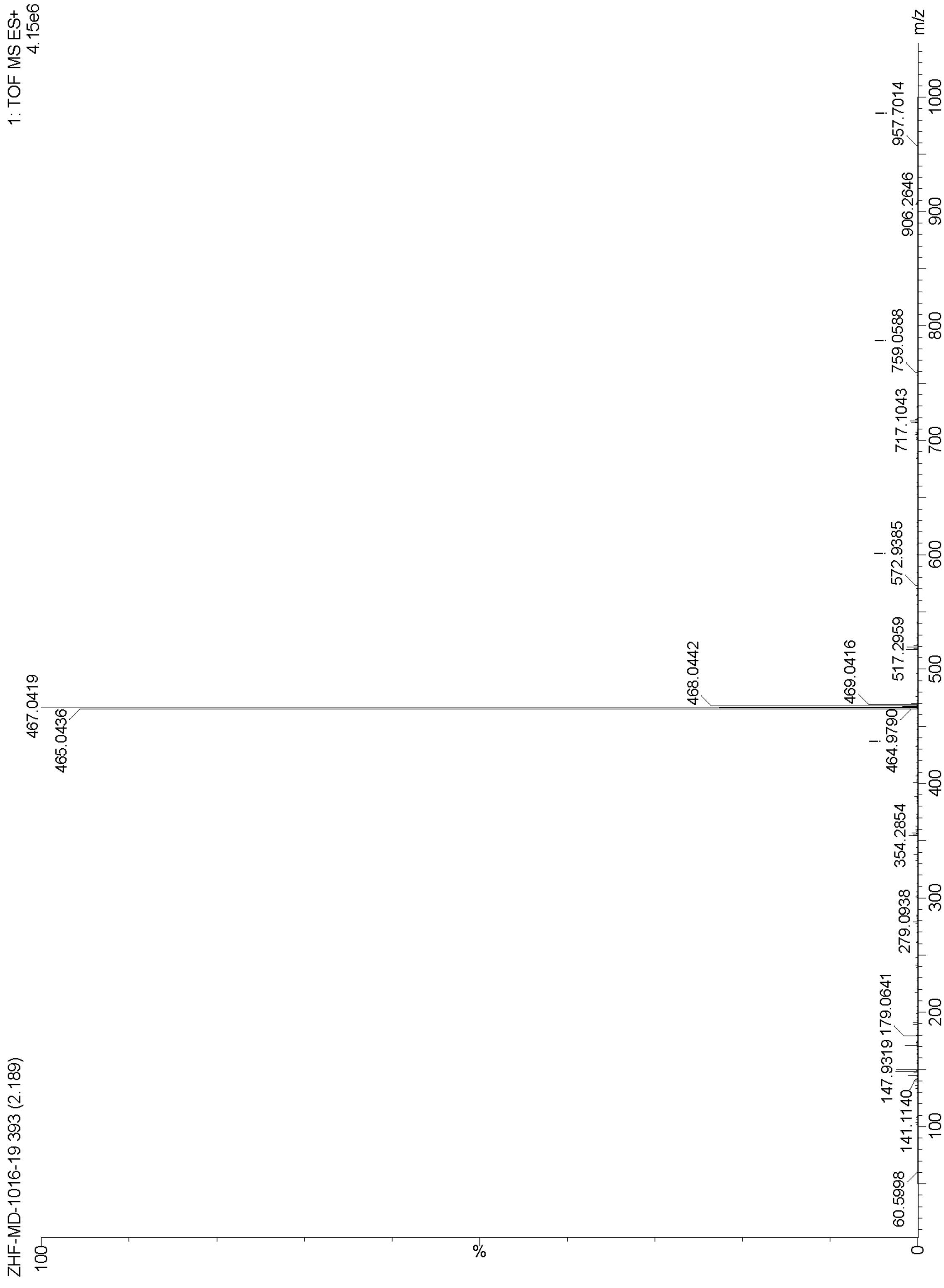

Table S54. HRMS of compound 6n

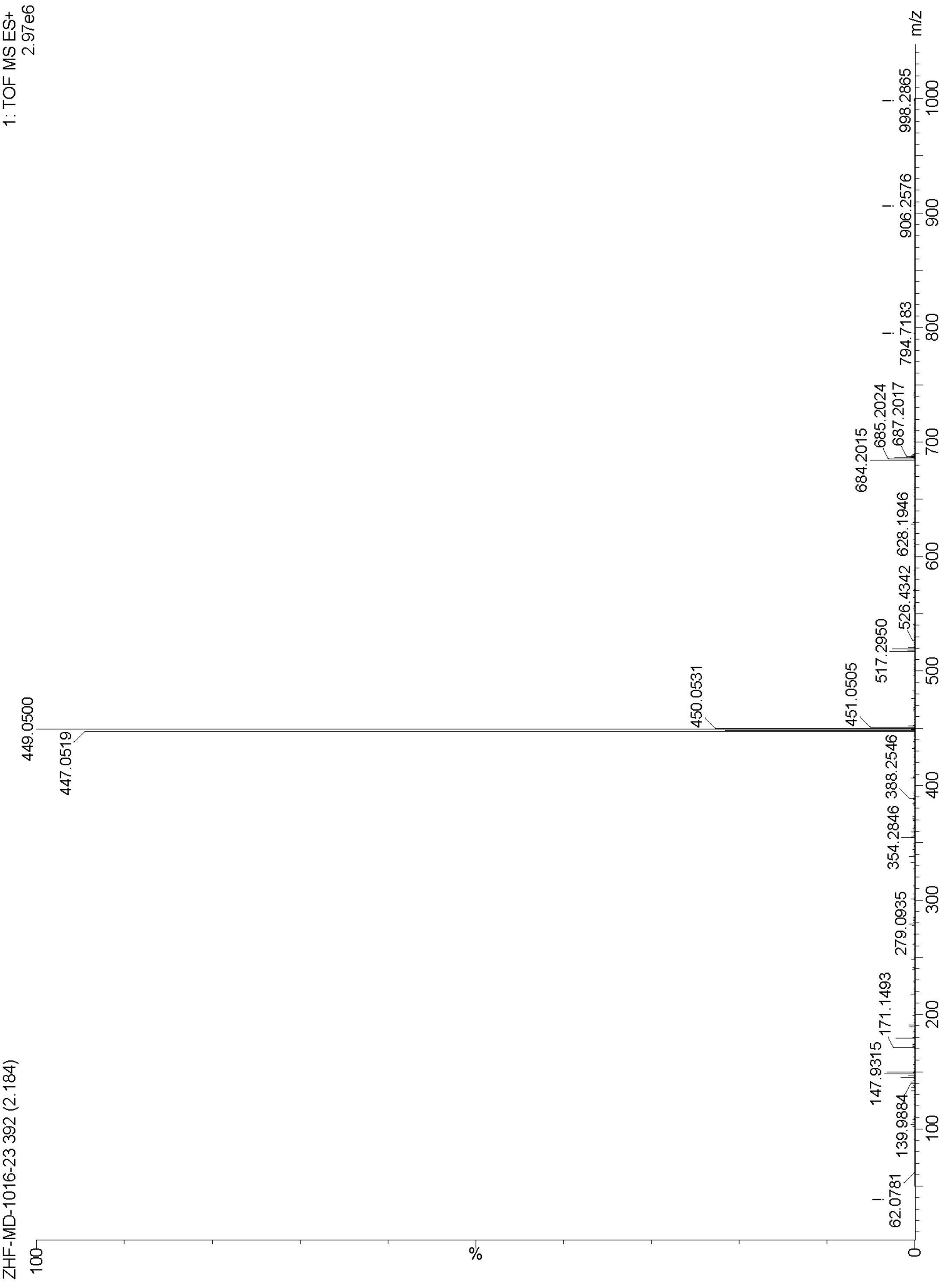

Table S55. HRMS of compound 6o

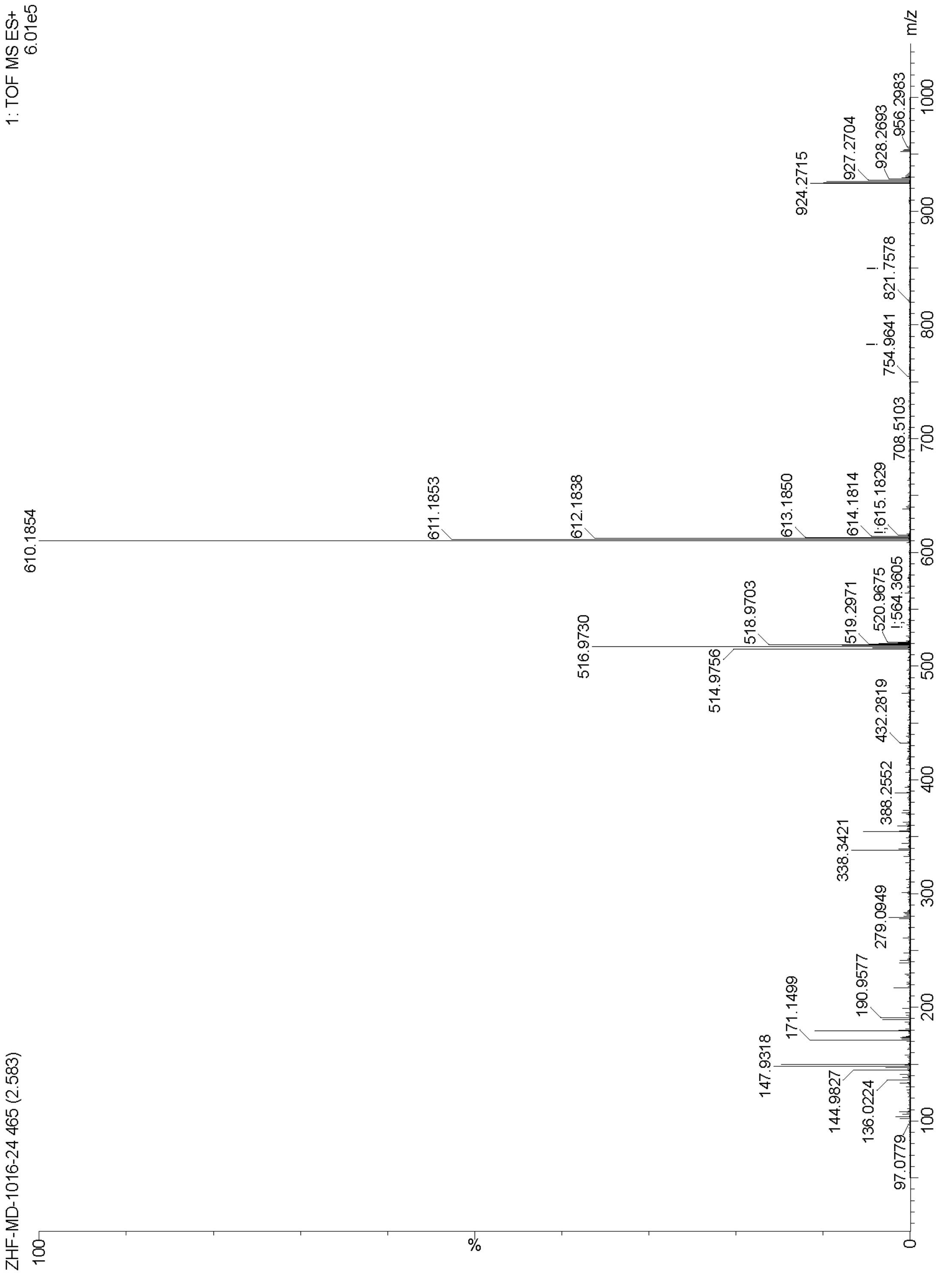

Table S56. HRMS of compound 6p

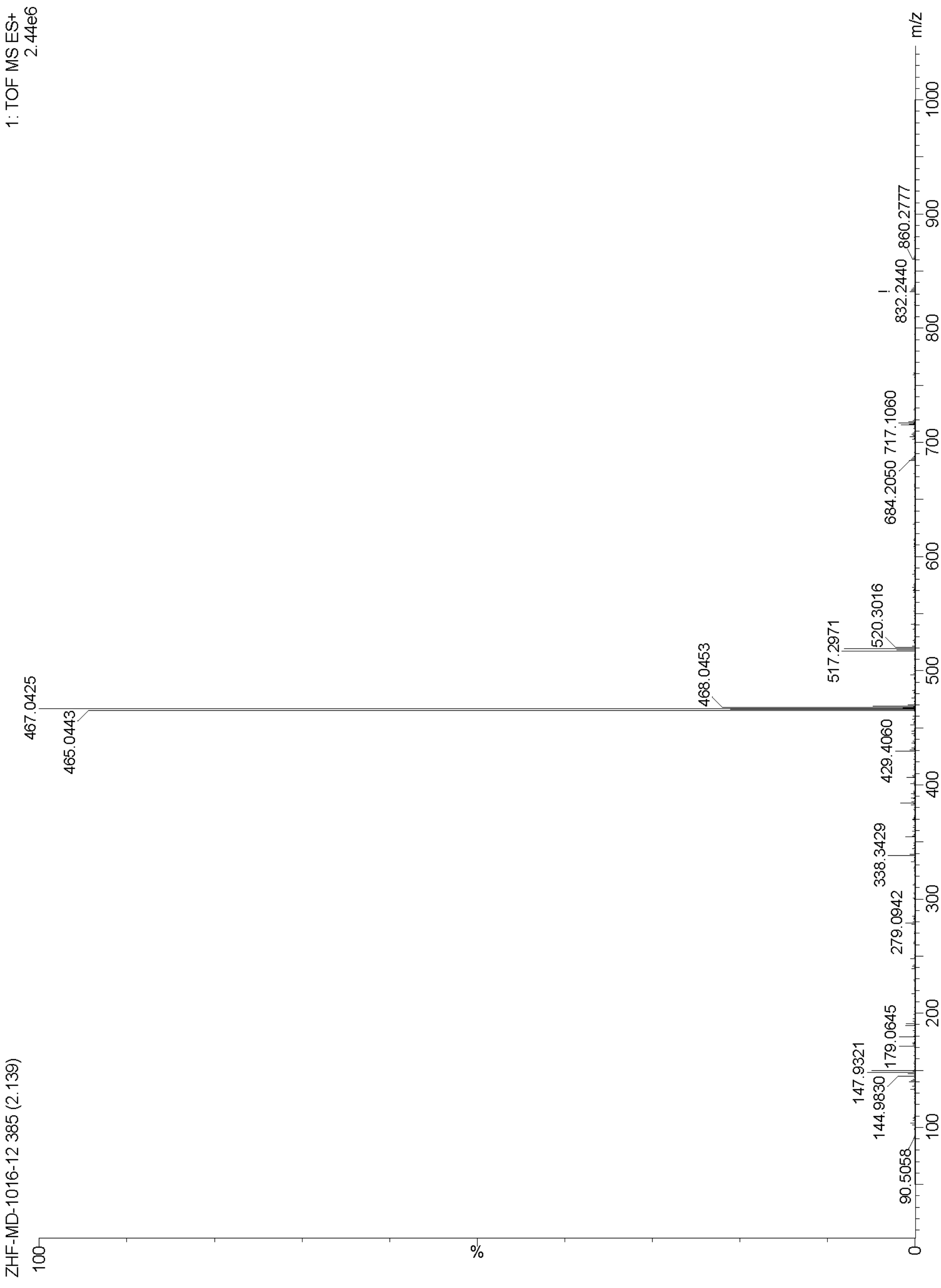

Table S57. HRMS of compound 6q

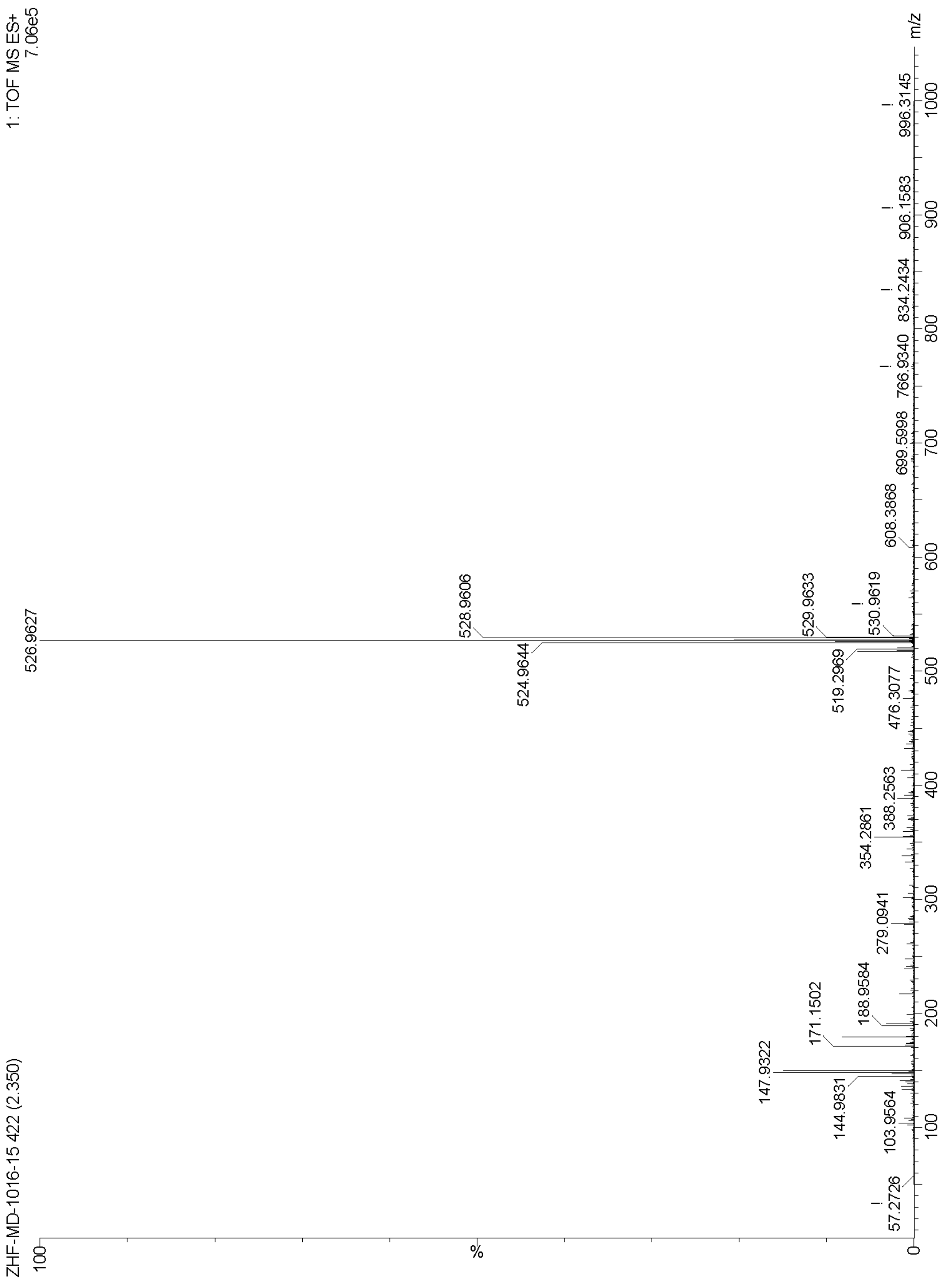

Table S58. HRMS of compound 6r

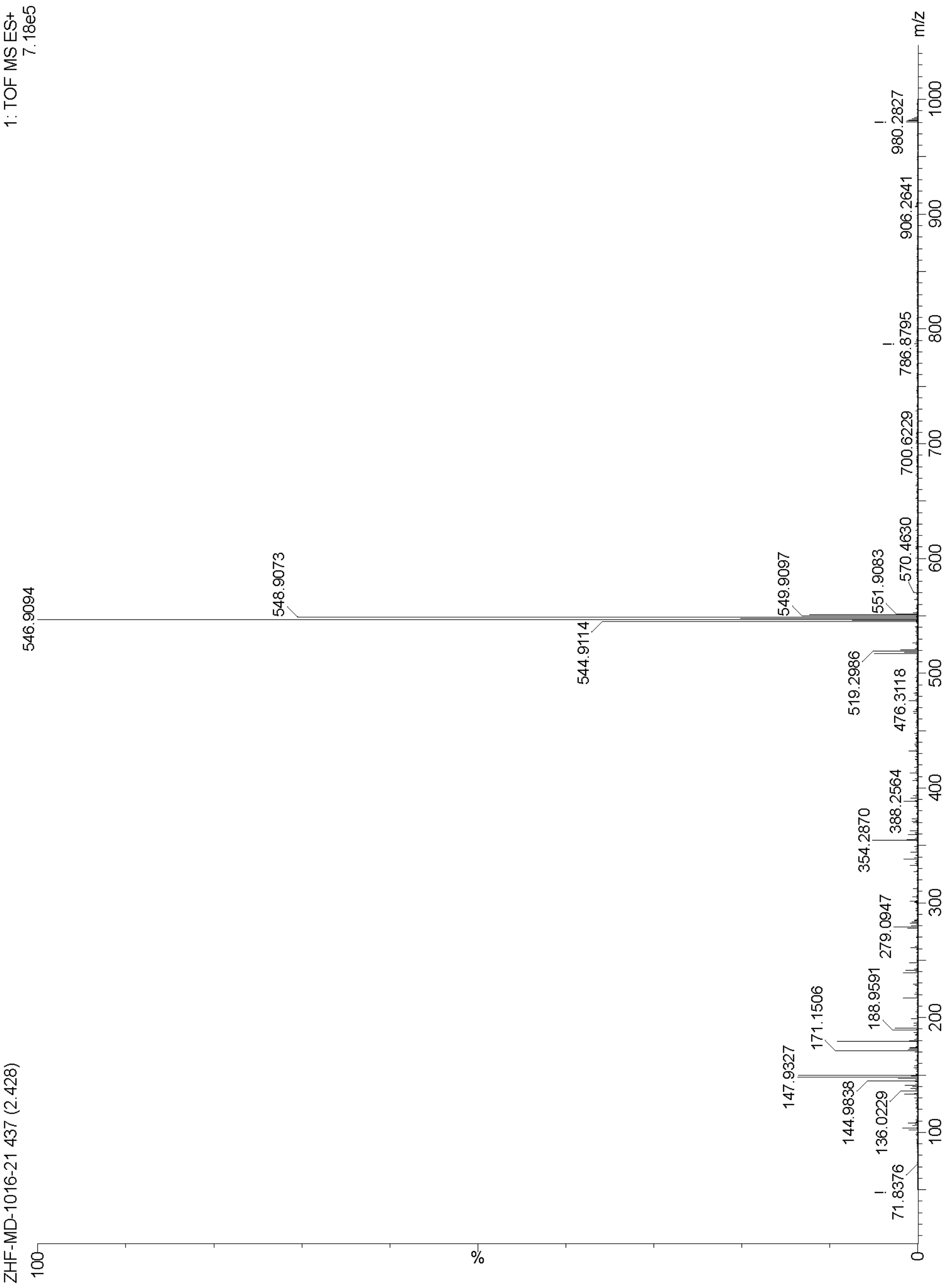

Table S59. HRMS of compound 6s

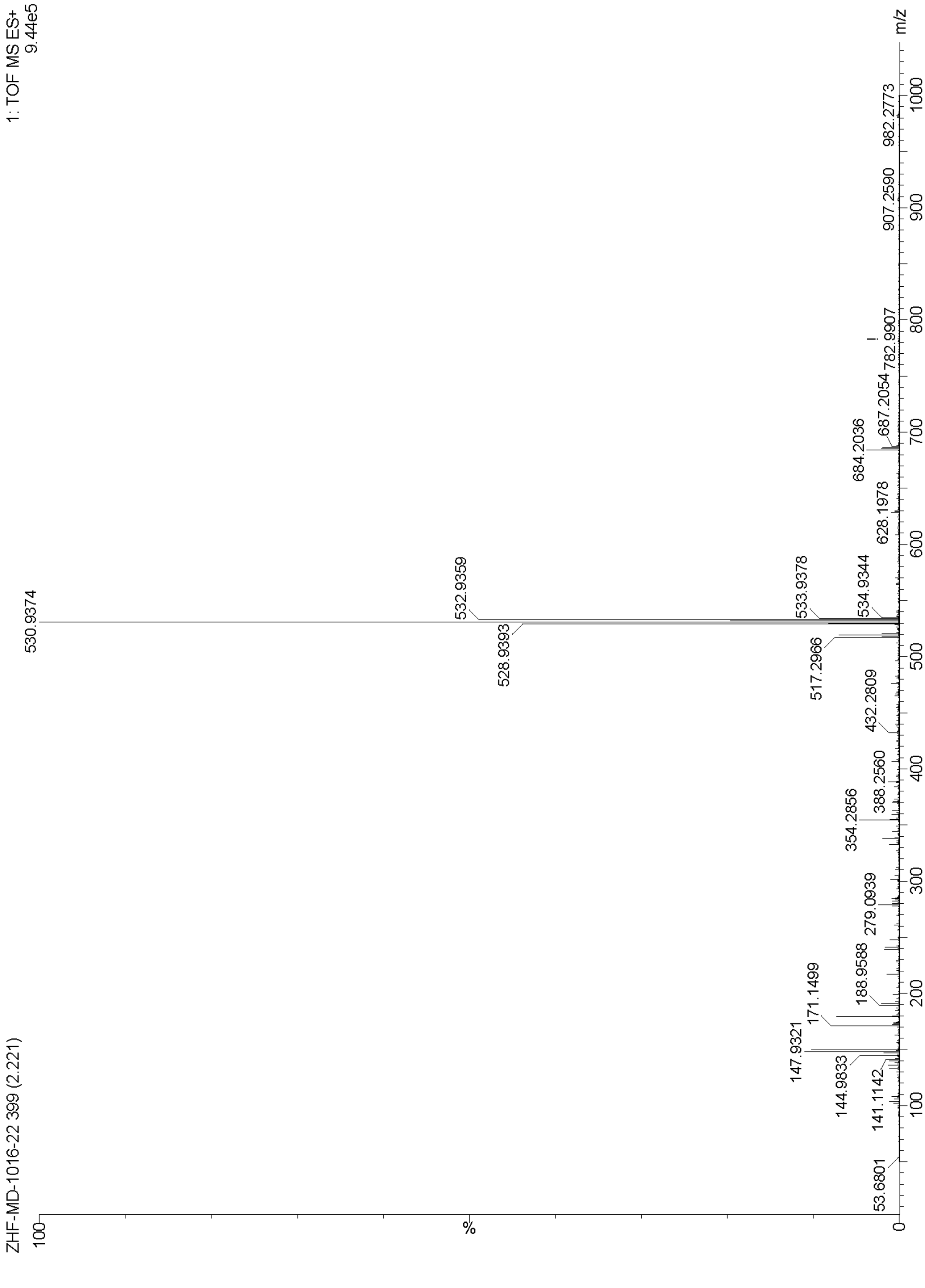

Table S60. HRMS of compound 6t
